# Combining cluster surveys to estimate vaccination coverage: Experiences from Nigeria’s multiple indicator cluster survey / national immunization coverage survey (MICS/NICS), 2016–17

**DOI:** 10.1016/j.vaccine.2020.05.058

**Published:** 2020-09-03

**Authors:** Dale A. Rhoda, John Ndegwa Wagai, Bo Robert Beshanski-Pedersen, Yusuf Yusafari, Jenny Sequeira, Kyla Hayford, David W. Brown, M. Carolina Danovaro-Holliday, Fiona Braka, Daniel Ali, Faisal Shuaib, Bassey Okposen, Eric Nwaze, Isiaka Olarewaju, Adeyemi Adeniran, Modibo Kassogue, Denis Jobin, Tove K. Ryman

**Affiliations:** a Biostat Global Consulting, Worthington, OH, USA; b Independent Consultant, Nairobi, Kenya; c DQ International Household Survey Consulting, Sofia, Bulgaria; d Bill & Melinda Gates Foundation, Abuja, Nigeria; e Johns Hopkins University, Baltimore, MD, USA; f Brown Consulting Group International, Cornelius, NC, USA; g World Health Organization, Geneva, Switzerland; h World Health Organization, Abuja, Nigeria; i National Primary Health Care Development Agency, Abuja, Nigeria; j National Bureau of Statistics, Abuja, Nigeria; k UNICEF, Abuja, Nigeria; l UNICEF, NY, USA; m Bill & Melinda Gates Foundation, Seattle, WA, USA

**Keywords:** Nigeria, Immunization, Vaccination coverage, Multiple Indicator Cluster Survey (MICS), Pooled data, Cluster survey

## Abstract

In 2015 immunization stakeholders in Nigeria were proceeding with plans that would have fielded two nationally representative surveys to estimate vaccination coverage at the same time. Rather than duplicate efforts and generate either conflicting or redundant results, the stakeholders collaborated to conduct a combined Multiple Indicator Cluster Survey (MICS) / National Immunization Coverage Survey (NICS) with MICS focusing on core sampling clusters and NICS adding supplementary clusters in 20 states, to improve precision of outcomes there. This paper describes the organizational and technical aspects of that collaboration, including details on design of the sample supplement and analysis of the pooled dataset. While complicated, the collaboration was successful; it yielded a unified set of relevant coverage estimates and fostered some novel sub-national results dissemination work.

## Highlights

• Nigeria's immunization stakeholders cooperated on the 2016–17 MICS/NICS survey.
• Extra survey clusters were added in 20 states to improve outcome precision.
• Data from MICS & supplementary clusters were pooled after passing a statistical test.
• Combined results were used to guide policy, but not as precise as originally hoped.
• We explore organizational aspects of cooperation & technical aspects of pooled data.

## 1 Introduction

Estimates of vaccination coverage are an important source of information for immunization program managers and periodic surveys are a key source of coverage information [1], [2], [3]. There are three primary surveys used for this purpose: USAID-supported Demographic and Health Surveys (DHS), UNICEF-supported Multiple Cluster Indicator Survey (MICS), and country- or donor-supported National Immunization Coverage Surveys (NICS). DHS and MICS are standardized across time and countries and capture data on topics far broader than vaccinations alone [4]. They are typically conducted every five years in a country, pending available funds. In countries with both DHS and MICS surveys, there is usually an effort to align so that one survey or the other happens every 2–3 years. NICS are less standardized although many are conducted using guidance from the World Health Organization’s (WHO) Expanded Programme on Immunization (EPI) and are widely known as *EPI surveys* [5]. In Nigeria (and in this paper) we use the phrase NICS. It is recommended that low and lower-middle income countries conduct at least one survey estimating vaccination coverage every 5 years. Country EPI programs usually prefer NICS because the program office is engaged in the design and implementation and additional program-relevant questions are often included such as reasons for non– and under-vaccination [6].

Nigeria conducted a combined MICS-NICS survey in 2016/17. The main survey methods and results are summarized in both a MICS report and a NICS report [7], [8]. This paper describes coordination and technical decisions that went into implementing this hybrid survey approach and presents some methodological findings, using Penta3 coverage as a key indicator. Lessons learned here may be valuable to future survey planners in Nigeria or other countries that would like to leverage upcoming MICS or DHS surveys for vaccination coverage estimates.

The objectives of this paper are to describe:

• the factors leading to the decision combining both surveys;
• technical aspects of sample size calculation and how data were pooled;
• some consequences of combining both surveys;
• lessons learned.

## 2 Factors leading to the decision to combine MICS and NICS

In Nigeria, DHS and MICS surveys are regularly conducted using alternating schedules. Since 2000, Nigeria conducted a NICS in 2003, 2007 and 2010, a DHS in 2003, 2008, 2013, and 2018 and a MICS in 2007, 2011, and 2016 [7], [9], [10], [11], [12], [13], [14], [28]. Nigeria's government, under leadership of the National Primary Health Care Development Agency (NPHCDA), planned a NICS in 2014 to follow the DHS but to precede the next MICS, which would be conducted under the leadership of the National Bureau of Statistics (NBS). However, the NICS was delayed, so in 2015 the country was faced with the potential of conducting both a NICS and a MICS in the same year, with risks of duplication, suboptimal use of resources, and survey fatigue. Between 2000 and 2015, 61 countries have conducted a NICS and DHS or MICS surveys within one year of each other; 17 of those in the same year, often leading to conflicting results. [6]

In early-2015 NPHCDA and immunization partners explored and excluded various options. Proceeding with separate MICS and NICS was determined to be a not only a poor use of time and resources, but would also threaten programmatic coherence, running the risk of having contradictory MICS and NICS vaccination coverage estimates. Canceling the NICS and proceeding with only the MICS would create challenges for the country EPI program and partners as the MICS survey does not include some immunization specific questions considered important and the MICS sample alone would likely yield state-level coverage estimates that would be considered imprecise (i.e., some states would estimate Penta3^1^ coverage with precision wider than ± 10%). Furthermore, a MICS-only solution would run the risk of leaving important federal partners feeling disengaged.

A key reason for estimating precise state-specific results was a requirement for baseline figures as some states prepared to transition away from Gavi support and toward paying for some of their vaccines^2^. Increasing the sample size of the MICS survey to improve vaccination coverage precision would mean substantial additional time and money for an already very large and complex household survey and there was justified concern from the MICS team about increasing the non-sampling error.

A hybrid approach, supplementing the MICS with *NICS clusters*, was identified as a viable option. Specifically, the standard MICS methodology would be used in Nigeria, but in 20 supplement states where the MICS sample was likely to yield Penta3 coverage estimates with confidence intervals wider than ± 10%, additional NICS clusters would also be sampled. In all states, the survey would include questions regarding barriers to vaccination and location of vaccination. In the supplementary clusters, all methodological and organizational aspects of the survey would be identical to the MICS, however, the fieldwork would be completed by a dedicated NICS-only team and only demographic and vaccination indicators would be collected, making data collection less burdensome. To assess comparability and proceed with pooling data from MICS and supplementary clusters, a statistical poolability test was to be performed. It was agreed that Nigeria would acknowledge the vaccination coverage based on a weighted analysis of MICS and supplementary clusters pooled together.

## 3 Methods

The Nigeria MICS survey planned to produce representative coverage estimates in each of the 36 states plus the Federal Capital Territory of Abuja; from here forward we will refer to these as *the 37 states*. Sixty enumeration areas (EA) were sampled from each state except the most populous states of Kano and Lagos, where 120 EAs were sampled in each for additional independent reporting. We refer to these EAs as *MICS clusters* to differentiate them from the *supplementary clusters*. Within each EA, 16 households were sampled from an updated list of households, according to a standard MICS protocol^3^ [15]. The fertility rate in Nigeria does vary substantially, especially from north to south, but the number of target households per EA was fixed at 16 for the entire country. The number of children surveyed in each EA depended on the number of children found in the 16 sampled households. Vaccination surveys typically sample and ascertain coverage for children 12–23 months of age to estimate coverage.

### 3.1 Governance of the MICS-NICS

Aligned with MICS protocols, the MICS-NICS survey was led by the NBS, and its governance mechanism included both a Steering Committee and a Technical Committee, to which all national and international stakeholders and partners were invited as members. The National Steering Committee for the MICS 2016 was constituted and inaugurated in November 2014 with responsibility to provide oversight, promote ownership and encourage financial contributions from development and national partners to the MICS-NICS survey. The Technical Committee reviewed the data gaps, advised on the list of indicators and questionnaire content, and advised on the sampling plan and sample design. The option of combining both surveys was presented to both committees for advice and endorsement.

Detailed plans for reporting vaccination coverage outcomes from the combined surveys were discussed in at least five steering committee meetings. MICS, by design, has stakes that goes beyond immunization and includes education, water and sanitation, protection, HIV and health, and poverty calculations that reflect an array of important issues for children and women. One key lesson from this experience is the importance for all discussions and decisions to be taken within the official governance mechanisms the country has established and to resist to the temptation to act outside these official structures. Vaccination-related decisions were duly informed with proper consultations in both of the committees and communicated in a transparent manner.

### 3.2 Supplementary sample size

Penta3 coverage is a globally recognized indicator of vaccination program performance and is included as part of the immunization indicators for the Sustainable Development Goals [16], Secs. 3.8.1 & 3.b.1], [17]. The MICS-NICS working group decided that each state should have a supplementary sample if it seemed likely by conservative projection that the state-level 95% confidence interval for Penta3 using MICS data alone might be wider than ± 10%. For simplicity, and to keep the supplement small compared to the MICS sample, the number of supplementary clusters was fixed to be either 10 or 20 or 30 per state. On a state-by-state basis, the number of supplementary clusters was selected to narrow the anticipated CI to no wider than ± 10%, if possible.

To project estimates of Penta3 CI widths it was necessary to forecast values of sample size, design effect and Penta3 coverage. The average number of children per household is known to be higher in the north than south due to higher fertility rates and higher prevalence of polygamy. The MICS 2011 dataset and information about subsequent changes in MICS methods were used to calculate likely number of respondents aged 12–23 months per cluster in each state. The intra-cluster correlation coefficient (ICC) was assumed to be 0.33^4^ [5, Sec. Annex B1]. The state-specific design effect was estimated as ≈ 1 + (state-specific average respondents per cluster – 1) × ICC [5, Sec. Annex B1]. A very conservative estimate for Penta3 coverage would be 50% for each state (the estimate that would yield the widest CI), but some states were considered very unlikely to yield 50% coverage, so conservative projections were made using the following rules applied to MICS 2011 and DHS 2013 coverage estimates:

a) If the ± 2 standard error CI for DTP3^5^ for either MICS 2011 or DHS 2013 contained 50%, then 50% was selected as the conservative coverage estimate.
b) If the CIs from both surveys excluded 50%, then the conservative estimate was taken to be the ± 1 standard error CI bound that fell closest to 50%. (i.e., If the MICS ± 2 standard error CI was 60–75% and the DHS ± 2 standard error CI was 55–70% then 50% will not be selected as the conservative value. Calculate ± 1 standard error intervals for each survey and select the endpoint closest to 50%. If the ± 1 standard error intervals were 63.5–71.5% for MICS and 59.0–65.5% for DHS, then 59.0% was selected as the conservative coverage forecast.)

Therefore, states with DHS 2013 or MICS 2011 coverage very near 50% had 50% as the conservative projected coverage (N = 15) and states with substantially lower (N = 11) or higher (N = 11) coverage had projected estimates that were not 50% but were closer to 50% than either the DHS or MICS estimates.

**Fig. 1:**
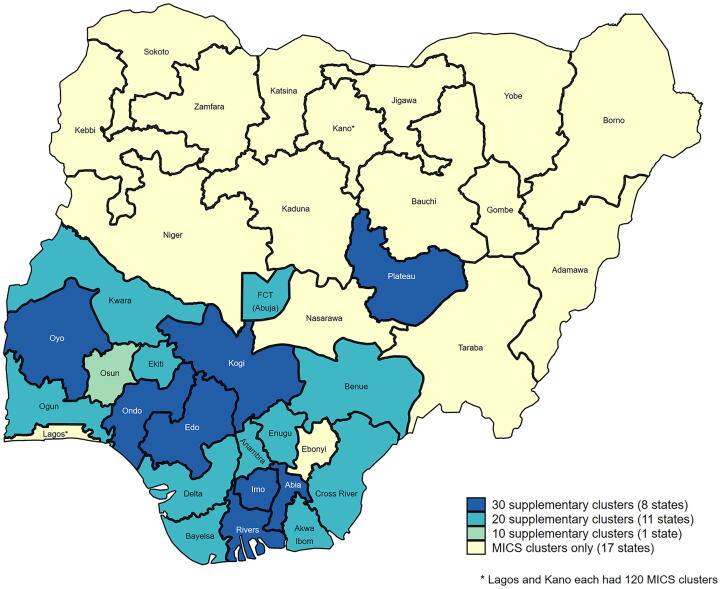
The twenty states with supplementary EAs were mostly in southern Nigeria.

**Fig. 2:**
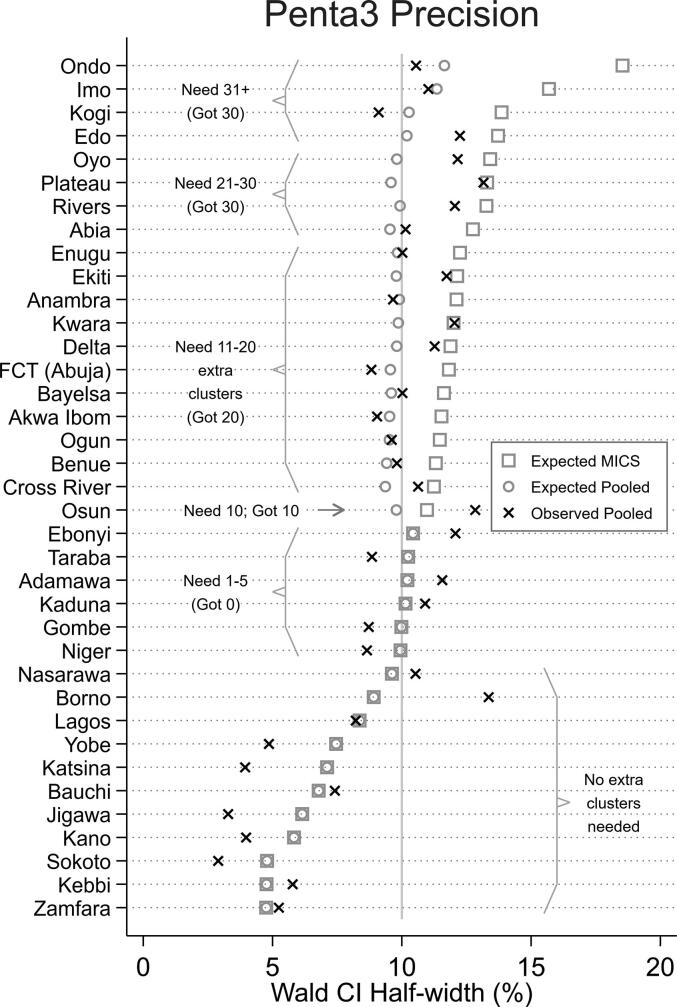
Nigeria’s states listed in increasing order of expected Penta3 confidence interval (CI) half-width considering the MICS sample only. Extra clusters in 20 states were intended to yield pooled sample CIs with half-widths below (or very near) 10% but for several reasons, the observed CI half-widths exceeded 10% in 19 of 37 states.

Supplementary clusters were employed in 20 of the 37 states (Fig. 1, Fig. 2 and the electronic supplements). Broadly speaking, coverage in southern states was anticipated to have wide confidence intervals due to lower numbers of children per household and having coverage near 50% whereas northern states were anticipated to have narrow confidence intervals due to higher numbers of children per household and lower coverage (Figure GS-5 in the supplement on CI widths). Supplementary clusters were added primarily in southern states to address the NICS precision goal for Penta3.

### 3.3 Sample selection

For the first stage of sampling, the primary sampling unit (PSU) sampling frame, probabilities and methods were identical for MICS and supplementary clusters. The MICS sampling expert supported Nigeria National Bureau of Statistics (NBS) in the design of both samples from the National Integrated Survey of Households round 2 (NISH2) master sample, based on a list of EAs prepared for the 2006 Population Census. Likewise, the second stage household listing and selection methods were identical in all clusters and were accomplished by the same teams. In every cluster, 16 households were randomly selected by a central office (not by field teams) to participate in the survey. MICS surveys ascertain coverage for children < 3 years of age and report coverage for children aged 12–23 months and 24–35 months at the time of survey. In MICS clusters, vaccination data were collected for all children under 3 years of age; in supplementary clusters, vaccination data were collected only for children aged 12–23 months, to contain costs and focus attention on very recent performance of the immunization program.

### 3.4 Questionnaire development, training & data collection

NBS supported questionnaire design and implemented data collection for both the MICS and supplementary clusters. Having a single organization in charge helped ensure that design and implementation approaches were as comparable as possible. The standard MICS questionnaire was revised (as it is the practice to tailor MICS questionnaires to the national context) to include questions on barriers to immunization and site of services; these revisions resulted in a restructuring of the order and flow of the MICS standard questions. The questionnaire for supplementary clusters was a pared-down version of the MICS questionnaire. Although the work was conducted by a single organization, different teams of interviewers were used for MICS vs. supplementary clusters. In supplementary clusters, interviewers administered only the household characteristics and vaccination modules from the MICS questionnaire so interviewers for those clusters completed only the subset of MICS training that was relevant for those two modules. MICS teams each included 4 interviewers and a supervisor; each team covered 30 clusters. Supplementary teams included 3 interviewers and a supervisor, including a mix of female and male representation; each team covered 10 clusters.

For both the MICS and supplementary clusters, households with no eligible respondents were not replaced in the sample. The questions in the household and vaccination modules and the hardware and software used for data collection were identical in MICS and supplementary clusters.

Staff from NPHCDA participated in interviewer training to teach interviewers how to read home-based vaccination records and field supervisors were trained on how to conduct spot-checks and overall daily reviews of interviewer performance. In both MICS and supplementary clusters, fieldwork was monitored by the NBS survey team and international MICS experts according to the standards recommended by the MICS Programme through a combination of field monitoring visits and central office reviews of customized field check tables in near real time. Cluster revisits for the purpose of quality control were not included, as this is not part of the standard field monitoring practices of MICS.

### 3.5 Methods to assess poolability of MICS & supplementary data

Before pooling data from the two sources, the datasets were assessed for substantial evidence of differential bias. Table 1 lists potential sources of bias that were identical and those that differed between the MICS and supplementary data collection efforts.

**Table 1:** Factors that could differentially bias results and whether they differed in MICS vs supplementary clusters.

Factor	Identical	Different
Eligibility criteria for respondents and children 12–23 m old	X	
Questions for children 0–11 and 24–35 m old		X (Not included in questionnaire for supplementary clusters)
Time of year of field work	X	
Period over which caregivers had to recall vaccination history	X	
Length of questionnaire or interview		X (MICS much longer)
Survey questions	X	
Data collection hardware (tablet model)	X	
Data collection software (program & version)	X	
Implementing agency	X	
Organizations who monitored field work	X	
Field team training		X (NICS was similar but shorter)
Field teams		X
Time that field teams spent in each cluster		X (3 days for MICS vs. 1.5 for NICS)
Data cleaning procedures	X	

Most factors were identical from the design perspective, but the NICS working group was mindful of setting a precedent and wanted all interested parties to know that the data would not be pooled if there was compelling evidence of differential bias, so an *a priori* plan was established to look for differences using a statistical test. Detailed methods description and poolability results are available in the NICS report and an electronic supplement to this manuscript [8].

Briefly, the test evaluated three survey data quantities in MICS vs. supplementary clusters in each of the 20 supplementary states: 1) % of households with a child 12–23 m; 2) % of children 12–23 m who produced a home-based record (card) that had one or more vaccination dates on it; and 3) % of children 12–23 m who had evidence (from either card or recall) of receiving Penta3. Coverage differences between MICS and supplementary clusters were evaluated using a permutation test and adjusted for 60 multiple comparisons [18], [19], [20]. To declare the data from a state *not poolable* the method required that at least one outcome difference between MICS-only and supplementary clusters be statistically significant with an adjusted p-value smaller than 0.01. Recall that the common procedure is to declare a difference to be significant if the p-value is smaller than 0.05. In this work, the planners knowingly adopted a more liberal rule to make it very likely that the expensive supplementary-NICS data would be incorporated into combined coverage estimates in every state unless there was unambiguous evidence of egregious differential bias.

The NICS working group was wary of the possibility of widespread confusion if different sets of official results were estimated using: MICS clusters only, supplementary clusters only, and pooled data from both sorts of clusters. So while the survey was still in the planning stages, commitments were secured in writing to say that the government would recognize only one set of coverage estimates: in states that passed the poolability test, coverage would be estimated using pooled data from MICS and supplementary clusters and in any state that failed the poolability test, coverage would be estimated from MICS clusters alone.

### 3.6 Survey weights

The Nigeria 2016/17 sample is not self-weighting; different sampling fractions were used in each state so weights were calculated using the sample design and selection probabilities and then adjusted for non-response using respondents aged 12–23 months from MICS and supplementary clusters [8]. In accordance with standard MICS practice, the weights were not post-stratified; the implicit assumption is that the sums of weights in the sample are at least as likely to reflect the current relative population sums across states than would a list of figures projected forward from the 2006 Nigeria National Census.

### 3.7 Indicators & analytic decisions

Vaccination coverage indicators in MICS reports are usually calculated using UNICEF’s freely available and customizable SPSS syntax [21]. In this survey, the NICS steering committee wanted to report additional indicators to assess timeliness of vaccination and prevalence of missed opportunities for simultaneous vaccination. The pooled dataset was analyzed using the World Health Organization (WHO) Vaccination Coverage Quality Indicators (VCQI) tool which is a set of freely available and customizable Stata programs that implement vaccination coverage analyses in accordance with recent WHO guidance [5], [22]. The coverage results reported in both the NICS and MICS reports were calculated using VCQI.

### 3.8 Dissemination

Seven sets of fact sheets were developed to summarize national results and results in each of Nigeria’s six geographic zones^6^ [23]. The sheets presented key messages in a visually appealing and statistically robust format targeting national and zonal policymakers and immunization program leaders. The double-sided colour A4-size info sheets were shared with state and zone level decision makers and public health leaders at a series of six briefing meetings between August and November 2017 [23]. Each meeting reviewed key NICS findings and featured group exercises to develop state-specific plans of action. Meeting participants completed a brief feedback questionnaire describing which messages, figures and graphics were most impactful. Questionnaire respondents (n = 74) reported focusing on the key messages highlighted on the first two pages and comparing/benchmarking performance of a state with other states and with national goals. They planned to use the fact sheets primarily for routine immunization planning, advocacy with partners and policymakers, and social mobilization.

Although the format of each fact sheet was standardized across zones, survey results and key messages were tailored to each zone and aimed to highlight vaccination coverage results across states within the zone, at the national level and in Sub-Saharan Africa broadly. See electronic supplement for a template. National or internationally recognized vaccination coverage goals (e.g., 90% crude coverage for Penta3) were presented as benchmarks. Reasons for non-vaccination, risk factors for missing vaccinations, and gaps in vaccination delivery were also presented. A set of informational graphics were also developed for social media messaging by the Nigeria National Emergency Routine Immunization Coordination Centre, National Public Health Care Development Agency and local partners.

## 4 Results

### 4.1 Implementation

Table 2 is a timeline of survey milestones. Data collection commenced at roughly the same time for the MICS and NICS teams. Supplementary interviewers finished before the MICS interviewers because they visited fewer clusters and administered a shorter questionnaire; this also meant that supplementary teams spent less time in each cluster than MICS teams.

**Table 2:** Timeline of Survey Milestones.

Milestone	Year/Month
Planning commences for 2014 NICS – delayed for lack of funding	2013
Planning commences for MICS	2014
	01/2015
NPHCDA & BMGF consider combined approach	02/2015
	03/2015
	04/2015
	05/2015
	06/2015
Approach UNICEF & NBS with the idea	07/2015
WHO involvement begins	08/2015
Agree on organizational roles	09/2015
Finalize immunization questionnaire & supplementary sample size	10/2015
	11/2015
	12/2015
	01/2016
	02/2016
	03/2016
	04/2016
	05/2016
Agree on poolability criteria	06/2016
	07/2016
	08/2016
MICS data collection begins	09/2016
Data collected in NICS clusters	10/2016
	11/2016
	12/2016
MICS data collection ends	01/2017
Poolability results available	02/2017
	03/2017
Clean MICS & NICS datasets available	04/2017
MICS & NICS weights available	05/2017
Weighted results available	06/2017
NICS report published	07/2017
	08/2017
	09/2017
MICS report published	10/2017
	11/2017
MICS-NICS dissemination meetings in the 6 zones - one report	12/2017
MICS data posted on NBS website	2018
MICS + supplementary data posted on NBS website	2019
BMGF: Bill & Melinda Gates Foundation	Acronyms:
MICS: Multiple Indicator Cluster Survey	
NBS: National Bureau of Statistics	
NICS: National Immunization Coverage Survey	
NPHCDA: National Primary Health Care Development Agency	
UNICEF: United Nations Children's Fund	
Items listed beside a month mean that the milestone was achieved in that month.	

### 4.2 States with supplementary clusters

Twenty states had supplementary clusters as part of the MICS/NICS (Fig. 1). Twenty-six states were identified as likely to have a CI wider than ± 10%, however six of those would have needed fewer than five additional clusters so it was decided not to sample any additional clusters there and instead hope the CIs would turn out to be narrower than the conservative projection (Fig. 2). This left 20 states in which supplementary clusters were sampled. Additional clusters were drawn in increments of 10 with a max of 30. Four states (at the top of Fig. 2) would have required >30 additional clusters to achieve a half-width ≤ 10%, but the sampling experts wanted the supplement to comprise no more than one-third of the total sample in any state, so those states received the maximum allowable count of 30 extra clusters.

### 4.3 Penta3 confidence intervals in supplementary states

While every state’s Penta3 half-width was < 14%, 14 of the 20 supplement states had CI half-widths wider-than-expected despite having used what were thought to be conservative assumptions in the sample size calculation (Fig. 2). Two factors appear to be primarily responsible for the surprisingly wide CIs (Table 3). In 15 states with supplementary clusters, MICS/NICS survey teams found substantially fewer children aged 12–23 m than were expected based on responses from the 2011 MICS. In 18 supplement states, MICS/NICS survey weights had notable variability. Those two factors work together to diminish the effective sample size and yield a wide CI; 10 of the 20 supplement states yielded an effective sample size for Penta3 that was smaller than 90% of the target figure. An electronic supplement quantifies the factors from Table 3 in more detail.

**Table 3:** Factors that yield wider confidence intervals than expected and recommendations for conservative sample size calculations for coverage surveys with cluster sampling.

Factor	Number of the 20 Supplement States Adversely Affected	Recommendation for future consideration(more conservative)
Observed coverage closer to 50% than expected	4	Either use 50% as the conservative projection or use the endpoint of the ± 2 standard error CI closest to 50%.
Effective sample size smaller than expected	10 had effective sample size < 90% of expected	Effective sample size is a function of number of respondents and design effect; see below.
Number of respondents with completed interviews smaller than expected	15 had total < 90% of expected	Assume the proportion of households that will yield an eligible respondent will be smaller than the proportion observed in the most recent survey; consider screening households for eligible respondents during the listing exercise and oversampling households known to have eligible respondents; because MICS was paired with NICS, consider oversampling households known to have children aged 12–23 m.
Design effect (DEFF) greater than expected	3 had DEFF > 110% of expected	Design effect has two components: a clustering term and a weighting term. [25] The clustering term is a function of average number of respondents per cluster and intracluster correlation coefficient. The weighting term is a function of heterogeneity in survey weights. The conservative projections for this project ignored the second term, but both terms should be considered in order to be very conservative.
Number of respondents per cluster greater than expected	0	Note that it is conservative to assume the total number of respondents in the stratum will be lower than expected but for DEFF clustering term purposes, it may be conservative to assume that respondents per cluster will be somewhat higher than the figure observed in the most recent survey.
Intracluster correlation coefficient (ICC) greater than expected	2 had ICC > 1/3	Oversample households with children 12–23 m to yield a larger sample and therefore a more numerically stable estimate of ICC.
Survey weights more heterogeneous than expected	18 of 20 had (1 + CV^2^_Wt_) > 1.1 (where CV_Wt_ is the coefficient of variation of the survey weights in the stratum); the expected DEFFs should have been inflated by an additional 10–40%	Incorporate a weight term in conservative DEFF calculations using the weights from a similar past survey.

### 4.4 Poolability

**Fig. 3:**
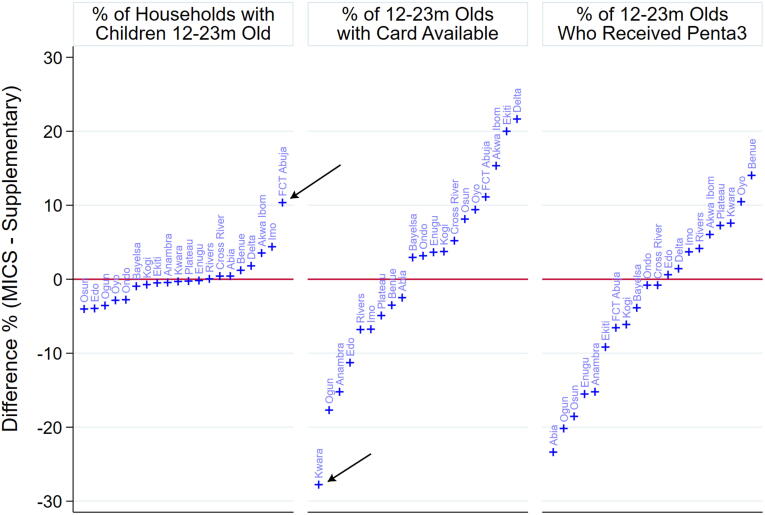
Observed differences between poolability criteria outcomes in MICS and supplementary clusters for three notable survey quantities. Although none of the differences was found to be significant in the permutation test, two differences that stand out from the rest are indicated here with black arrows.

The permutation test judged the data to be *poolable* in all 20 states with supplementary samples. The NICS report and an electronic supplement document detail results and p-values. Fig. 3 shows (MICS cluster mean – Supplementary cluster mean) differences for 20 states for the three outcomes in the permutation test. Note that for each outcome the differences are distributed in a roughly symmetric fashion around the value 0. Fig. 3 uses arrows to annotate two differences with noticeably larger magnitude than the rest, but after adjustment for multiple comparisons, all 60 differences were found to not be statistically significant, which led us to conclude that there was no strong evidence of differential bias.

If the study had used the traditional threshold for statistically significant differences, (p < 0.05) then the data would have been pooled in every state except FCT Abuja. The supplementary clusters there yielded a child 12–23 m in only 8% of households visited versus 18% for the MICS clusters; the adjusted p-value is 0.025. All other adjusted p-values were much higher than 0.05 (See the electronic supplement on poolability).

## 5 Discussion

We are happy to report that the National Bureau of Statistics (NBS) in coordination with the NICS steering committees successfully conducted contemporaneous vaccination coverage data collection in hundreds of supplementary clusters using the same procedures, questions and equipment as a full MICS survey. The data were similar enough to warrant pooled analysis and the coverage results were made available in a timely manner. To successfully implement this combined survey, several design and implementation decisions had to be made, some even as the survey was in progress, but all within the governance structures established by the lead implementing agency, NBS. Furthermore, dissemination meetings used customized materials to highlight notable region-specific results. The results from this survey were immediately useful, prompting establishment of a National Emergency Routine Immunization Coordination Centre (NERICC) to allocate special attention to 18 states to accelerate and monitor coverage improvements [24].

In the early planning stages, the steering committee was concerned that coordination challenges might be insurmountable or biases in the MICS and NICS supplementary clusters might be demonstrably different. When confronted with implementation decisions, the committee strove to ensure comparability between the two surveys. In retrospect some partners are satisfied with the level of coordination that occurred and feel that the inter-organization governance committees resolved the important and complicated issues, while others point out that even more effort could have been made to ensure all decisions were reviewed from both a policy and technical perspective.

By the time the poolability analysis revealed interesting differences in FCT Abuja and Kwara, the data collection teams had been dismissed. In retrospect, it might have been helpful to assess poolability in near-real-time and talk with those teams right away to understand possible sources of almost-significant differences. While there was no evidence of egregious differential bias or outright data tampering, it would have been useful to follow-up and understand the most notable differences. Future surveys may wish to simulate alarming levels of differential bias and data tampering to inform what p-value should serve as the threshold for statistically significant differences.

If the survey had been designed primarily for vaccination coverage, the sampling plan would likely have included more than 16 homes per cluster because many homes do not yield a resident aged 12–23 months. The proportion of clusters that yielded 0, 1 and 2 respondents aged 12–23 m were 8%, 15% and 18% respectively in northern states and 24%, 30% and 23% in southern states. This represents a trade-off for NICS stakeholders when deciding to forego a separate survey. When joining efforts, the vaccination study may benefit from all the rigorous, well-run aspects of a MICS survey but may need to accept that a notable portion of clusters will not contribute any respondents for vaccination coverage analysis. If supplementary samples are collected in future surveys it might make sense to over-sample households with children in those clusters, though, as this complicates management and implementation, a simpler method would be to draw a larger number of supplementary clusters or households per cluster.

In retrospect, the supplementary sample size assumptions should have incorporated even more conservative estimates of expected number of respondents aged 12–23 m and of design effect. The calculations assumed that each visited household would yield the same average number of vaccination respondents as MICS 2011, but more than half the states yielded fewer in 2016 than 2011. Fifteen of the twenty supplementary states yielded a vaccination coverage sample that was <90% of the expected size.

Design effect (DEFF) calculations for the sample size work omitted an important term in the conservative calculation. Kish describes the DEFF as a multiplicative product of two terms: the clustering term and the unequal weight term [25], [26], [27]. The clustering term is [1 + (*m*-1) × *ICC*], where *m* is the average number of respondents per cluster and *ICC* is the intracluster correlation coefficient; this term was used to estimate the DEFF for the MICS-NICS. The weighting term is [1 + CV^2^_Wt_] where CV_Wt_ is the coefficient of variation of the survey weights in the stratum; this term was omitted. For MICS sample size calculations, it is traditional to examine values of DEFF from an earlier survey and select a conservative value, but not to estimate the terms separately. For WHO sample size calculations, it is recommended to calculate the clustering term, but the weighting term has usually been ignored due largely to the fact that the weights in EPI surveys were typically all equal before the 2015 draft of the 2018 Cluster Survey Reference Manual [5].

Beyond the technical considerations, this supplementary sample approach required substantial coordination at many levels of the health system. Typically, vaccination coverage surveys are coordinated by the routine immunization (RI) working group with the National Primary Health Care Development Agency (NPHCDA) as the lead and WHO as the technical support. That was similar in this survey but added to that was that NBS was the implementing agency and UNICEF was the lead technical support agency for MICS. So, the number of core partners doubled and the potential for essential reviews to be missed greater (e.g., restructuring of the questionnaire flow). And as noted above, with emphasis on exchangeability and mitigating differential bias in these surveys, some of the decisions made were different than what the EPI program would have made if they were doing a standalone NICS (especially number of vaccination respondents per cluster).

The survey steering committee learned the value of close regular coordination and *a priori* written agreements. One key element of success here is that before the survey began, partners and stakeholders agreed to use the eventual pooled MICS/NICS estimate of coverage as the official survey estimate—including a written and signed letter of endorsement of the pooled estimates by the Executive Director of NPHCDA. This turned out to be particularly important because there was turnover in programmatic staff during the long period of survey planning and implementation, so without the written agreement, new staff might not have felt ownership or obligation to use the pooled results. Furthermore, in some states the MICS-only Penta3 coverage estimate was higher than the pooled estimate so without a high-level commitment to use pooled estimates, it could have been very tempting to use the MICS-only estimate in those states.

A benefit of the combined approach was that a broad set of perspectives were considered in the design, interpretation and communication of results. Several innovations in the dissemination strategy helped to highlight the survey results and reach a broad audience. Survey results were made available rapidly and shared at national and subnational briefing meetings in multiple formats. The national and zonal information sheets were developed to concisely summarize key findings from the survey report in a visually appealing format that could be read quickly and understood by technical and non-technical audiences. The subnational briefing meetings enabled immunization managers to discuss the survey results, assess the implication of the survey findings on their work, and develop state-specific action plans to improve vaccination coverage. Ongoing efforts are needed to support immunization managers to implement the action plans.

The survey showed that in many low-coverage states, most vaccinated children received services in secondary or tertiary public sector health facilities, not in primary health facilities. And even in urban areas with high numbers of private facilities (e.g. Lagos), the majority of children were being vaccinated in public, not private facilities. These findings led NERICC to recommend larger public health facilities who were not already offering daily RI sessions to start doing so, and for an added focus on missed opportunities in vaccination (e.g. linking a facility’s outpatient department and maternity ward with its immunization unit).

Nigeria’s declaration of a state of emergency for RI, spurred by the survey findings, also included a deep analysis of equity indicators such as coverage differences between urban and rural areas, wealth quintiles, and maternal education. Results from this analysis led to the selection of 18 State Emergency RI Coordination Centers (SERICCs). In these 18 SERICC states, NERICC understood the critical role that leadership and management play and required a competitive application process for program managers. NERICC also set tight deadlines for developing an ambitious array of innovative interventions. During both the setup of NERICC and the 18 SERICCs, a high level of continuous commitment and engagement by the Executive Director NPHCDA was critical to keeping momentum going. At the onset, NPHCDA aimed for the NERICC/SERICC structure to last 18–24 months, at which point a determination would be made for any follow up plans.

It can be challenging to find funding for standalone high-quality NICS surveys – especially those that report precise results for dozens of sub-national strata, so combined surveys may be an attractive solution in other settings. Given the experience in Nigeria we would encourage countries to think about ways to better leverage regular DHS/MICS surveys for their vaccination estimates with the aim of obtaining necessary data in the most efficient manner possible. However, one should not underestimate the level of agreements, coordination and technical expertise required.

## CRediT authorship contribution statement

**Dale Rhoda:** Methodology, Software, Formal analysis, Visualization, Writing - original draft. **John Ndegwa Wagai:** Methodology, Formal analysis, Data curation, Writing - original draft. **Bo Robert Beshanski-Pedersen:** Conceptualization, Methodology, Investigation, Writing - original draft. **Yusuf Yusafari:** Resources, Supervision, Writing - review & editing. **Jenny Sequeira:** Conceptualization, Methodology, Resources, Writing - review & editing. **Kyla Hayford:** Conceptualization, Methodology, Investigation, Visualization, Writing - original draft. **David W. Brown:** Conceptualization, Visualization. **M. Carolina Danovaro-Holliday:** Resources, Supervision, Writing - review & editing. **Fiona Braka:** Project administration, Investigation, Writing - review & editing. **Daniel Ali:** Investigation, Writing - review & editing. **Faisal Shuaib:** Supervision, Writing - review & editing. **Bassey Okposen:** Project administration, Writing - review & editing. **Eric Nwaze:** Supervision, Writing - review & editing. **Isiaka Olarewaju:** Project administration, Writing - review & editing. **Adeyemi Adeniran:** Formal analysis, Writing - review & editing. **Modibo Kassogue:** Project administration, Supervision, Writing - review & editing. **Denis Jobin:** Conceptualization, Funding acquisition, Project administration, Supervision, Writing - review & editing. **Tove K. Ryman:** Conceptualization, Methodology, Visualization, Project administration, Funding acquisition, Writing - original draft.

## Declaration of Competing Interest

The authors declare that they have no known competing financial interests or personal relationships that could have appeared to influence the work reported in this paper.

## Footnotes

1 Third dose diphtheria-tetanus-pertussis-*Haemophilus influenzae* type b (Hib)-Hepatitis B or Pentavalent vaccine.

2 At that time, transition was to start in 2017 and end in 2022; but in June 2018 the Gavi Board approved a Nigeria proposal to extend the transition to end in 2028.

3 The 2016–17 planned sample of 60 EAs and 16 households per selected EA represents a 20% increase in sample size over the MICS 2011 which sampled 40 EAs per state and 20 households per selected EA and a 28% increase over the MICS 2006 which sampled 30 EAs per state and 25 households per selected EA.

4 31 of 37 states had a DPT3 ICC < 0.33 in MICS 2011 as did 32 of 37 states in DHS 2013. The only state with ICC > 0.33 in both MICS & DHS was Ondo, and in the 2016-17 MICS/NICS survey, Ondo received 30 supplementary clusters, which was the maximum number allowed.

6 A zone in Nigeria consists of a group of 7–10 states, and a state can have 20–44 *districts* or LGAs — local government areas.

## Footnotes

5 The current formulation of Penta3 was introduced in Nigeria in 2012, so in this analysis, three doses of diphtheria-tetanus-pertussis (DTP3) was used as a proxy for Penta3 in earlier surveys.
