# Supplementary material for: Combining cluster surveys to estimate vaccination coverage: Experiences from Nigeria’s multiple indicator cluster survey / national immunization coverage survey (MICS/NICS), 2016–17
Source: Vaccine. 2020 Sep 3;38(39):6174–83. doi: 10.1016/j.vaccine.2020.05.058 (PMC7450266; doi:10.1016/j.vaccine.2020.05.058)
Supplement: Supplementary data 1 [file mmc1.pdf]

Supplement to

Combining Cluster Surveys to Estimate Vaccination Coverage: Experiences from Nigeria's Multiple Indicator Cluster Survey / National Immunization Coverage Survey (MICS/NICS), 2016-17

Vaccine (2020) doi:10.1016/j.vaccine.2020.05.058

Overview of Nigeria MICS/NICS 2016-17 Poolability Assessment Methods & Results

Dale Rhoda

BIOSTAT GLOBAL CONSULTING

Electronic Supplement

- This document is an electronic supplement to the manuscript

Combining Cluster Surveys to Estimate Vaccination Coverage: Experiences from Nigeria's Multiple Indicator Cluster Survey / National Immunization Coverage Survey (MICS/NICS), 2016-17

Vaccine (2020) doi:10.1016/j.vaccine.2020.05.058

- It describes the methods and results of the poolability analysis that is described in that manuscript and in the report named

  Nigeria National Immunization Coverage Survey 2016/17 (available here).

Goal

- The idea of the poolability test is to look for evidence of substantially different biases among data collected in the MICS clusters and data collected in the supplementary clusters
- If the processes that yielded these two datasets differ so much that differential biases are evident in the data, then we want to declare the data to be “not poolable”
- On the other hand, if we do not find striking evidence of different biases, we want to be able to pool the data to estimate state-level vaccination coverage outcomes with a larger sample, that will hopefully yield results that are precise enough to help manage the immunization programme

Methods

- The method that was adopted was to do three unweighted randomization tests in each of the 20 states that had supplementary clusters
- Three outcomes of interest were identified using the logic that these outcomes might differ substantially if there were different biases in the MICS vs. supplementary clusters:
    - % of households with a child 12-23m
    - % of respondents 12-23m who showed a card with 1+ vaccination dates
    - % of respondents 12-23m who received Penta3
- Calculate the observed values of each unweighted outcome for the group of MICS clusters in a state and for the group of supplementary clusters in that same state
- Then, in each state, calculate the difference between each outcome in MICS and supplementary clusters

Methods

- Each cluster is labeled as either “a MICS cluster” or “a supplementary cluster”
- For each iteration of the randomization test, we randomly jumble the labels on the clusters within the state; we re-assign the MICS versus supplementary labels at the cluster level, not the individual level, to maintain the within-cluster correlation structure of the data as (at least a partial) guard against inflated Type I errors that can result when correlated data are analyzed as if they were independent
- Recalculate the outcomes for the newly labeled MICS clusters and supplementary clusters; calculate the new difference
- Repeat 500,000 times per outcome per state

Notional Example

- In this slide we see an abbreviated dataset to demonstrate the method
- The state has seven MICS clusters and two supplementary clusters; clusters 8 and 9 are truly the supplementary clusters
- One outcome is shown for each cluster
- It is easy to calculate the MICS mean and the Supplementary mean, and their Difference

Cluster ID	Observed Cluster Label	% of kids 12-23m with cards
1	MICS	50
2	MICS	40
3	MICS	35
4	MICS	55
5	MICS	45
6	MICS	20
7	MICS	80
8	Supplementary	40
9	Supplementary	60

MICS mean = 46.4%
Supplementary mean = 50%
Difference = – 3.6%

Notional Example

- Now we list the clusters and outcomes in the same order but jumble the labels; we consider clusters 3 and 6 to be the supplementary clusters in this iteration
- Recalculate the MICS mean, the Supplementary mean, and their Difference
- Repeat 500,000 times, storing the difference each time

Cluster ID	Jumbled Cluster Label	% of kids 12-23m with cards
1	MICS	50
2	MICS	40
3	Supplementary	35
4	MICS	55
5	MICS	45
6	Supplementary	20
7	MICS	80
8	MICS	40
9	MICS	60

Jumbled MICS mean = 52.9%
Jumbled Supplementary mean = 27.5%
Difference = 25.4%

Methods

- Each state with supplementary sample was targeted for 60 MICS clusters and either 10 or 20 or 30 supplementary clusters
- In practice, some clusters were not accessible, and some clusters were reached but did not yield any children aged 12-23m
- Clusters that did not yield respondents are ignored in this analysis

Methods

- If the processes that generated the two datasets are identical, or free from differential bias, then we do not expect the cluster labels (MICS vs. Supplementary) to carry meaningful information, and so when we jumble the labels, the differences we observed from the true labels should not be extreme compared with the sets of differences observed with jumbled labels
- Calculate an unadjusted p-value for each of the 60 comparisons (3 outcomes of interest per state X 20 states) thus:

$$unadjusted\ pvalue = \frac{\begin{array}{c} Number\ of\ comparisons\ where \\ |jumbled\ difference| \geq |observed\ difference| \end{array}}{500{,}000}$$

Methods

- We want to adjust the rule for rejecting the null hypothesis to preserve an overall family-wise error rate for all 60 comparisons
- We use the Holm-Bonferroni method, described in later slides after we present some histograms of differences
- We also want to err on the side of pooling the data unless we see egregious evidence of differential bias, so before the data were collected, we decided to use a family-wise error rate of 1% across the 60 tests instead of the default traditional value of 5%
- That is to say that we want the probability that we will mistakenly declare one or more states to be “not poolable” to be smaller than 1%

MICS-only vs. Supplementary Distributions

- The next three slides show how the observed outcomes are distributed across MICS and supplementary clusters
- At first examination, the distributions look quite similar
- After seeing the later slides, you may come back to these and notice some differences

- Note that in this presentation, we use sometimes the term *core clusters* to refer to the MICS-only clusters

Households with Children 12-23m

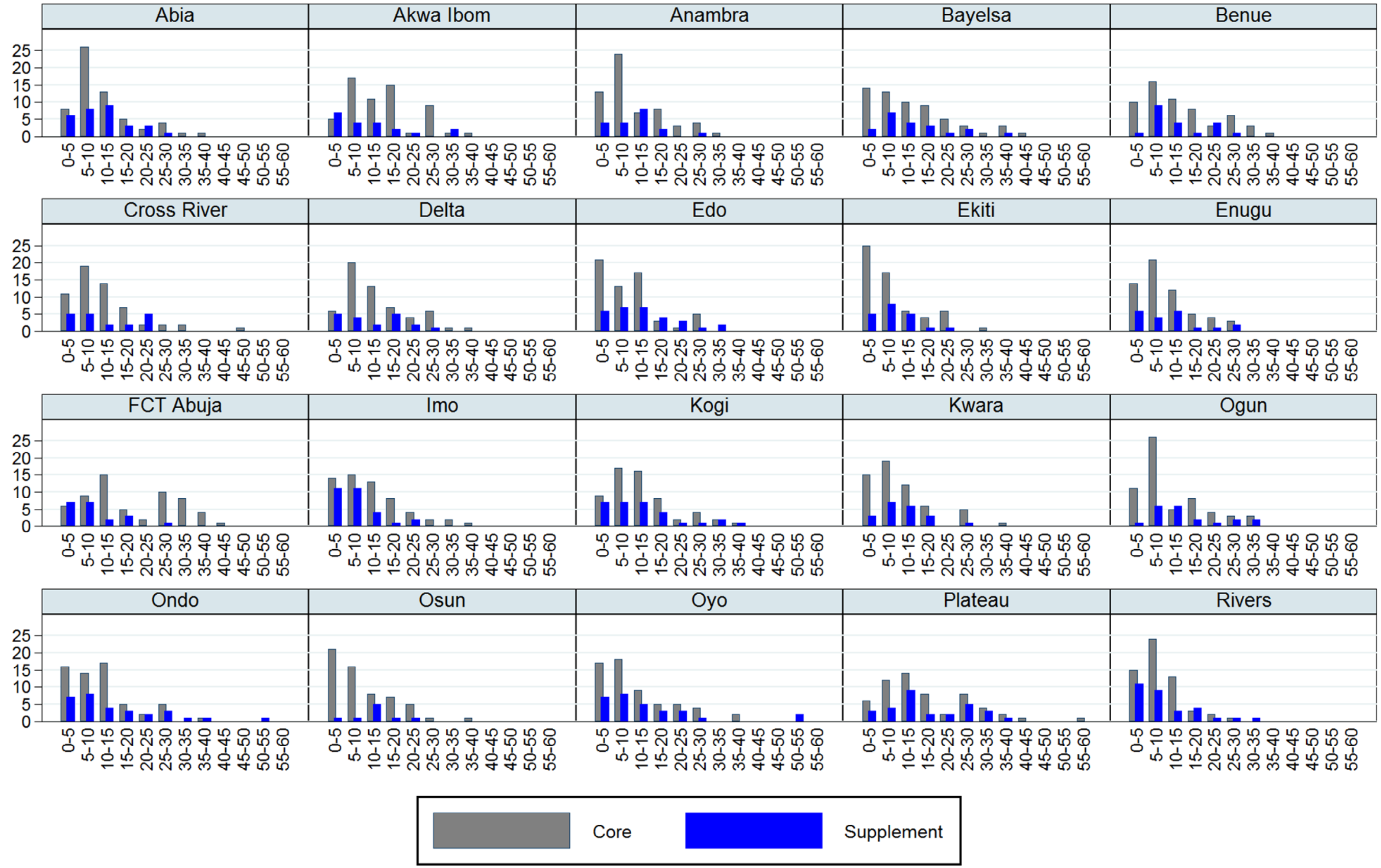

Y-axis is number of clusters and x-axis is the outcome (% of Households with Children 12-23m)

Children 12-23m with Card Available

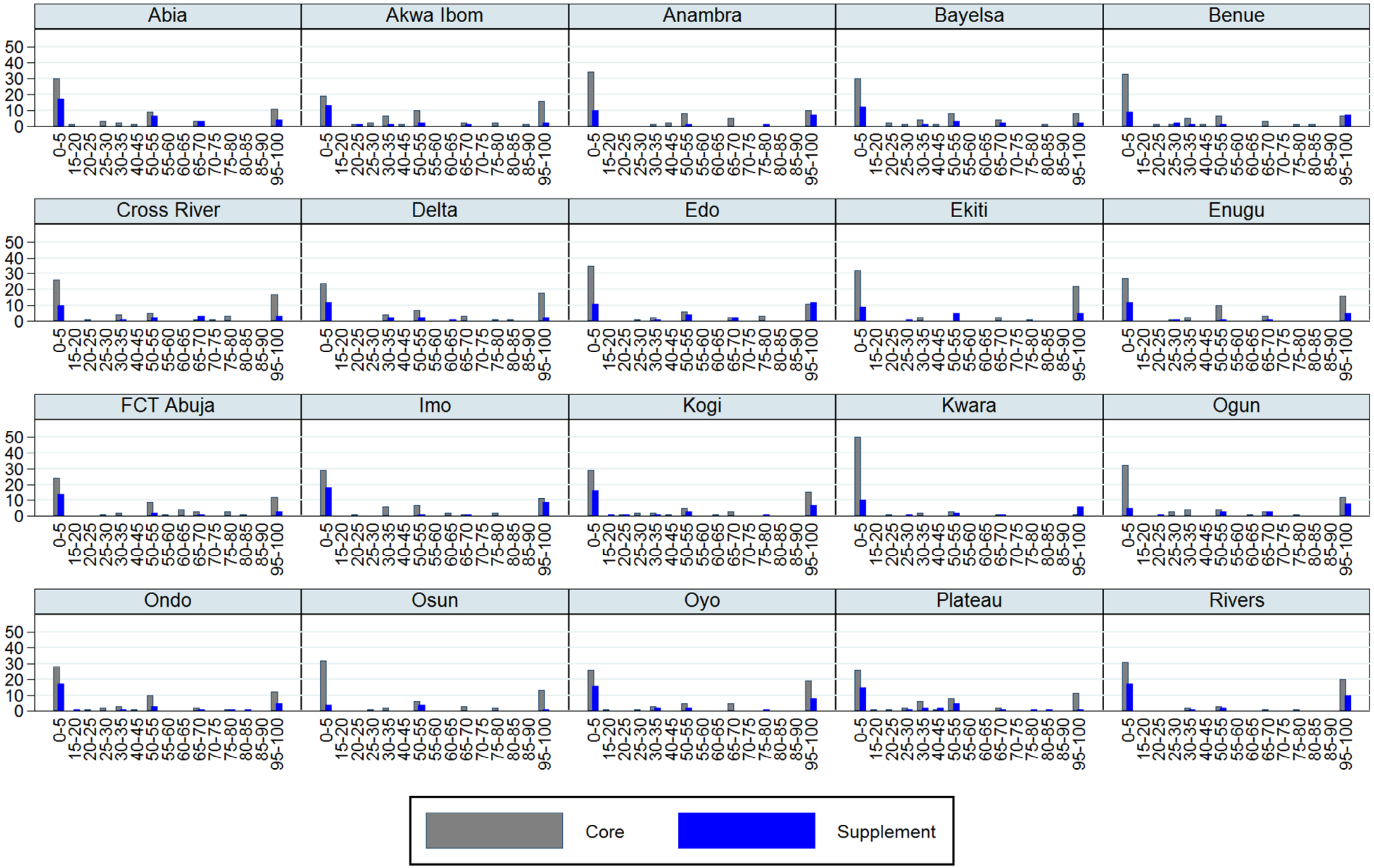

Y-axis is number of clusters and x-axis is the outcome (% of Children 12-23m with Card Available)

Children 12-23m who Received Penta3

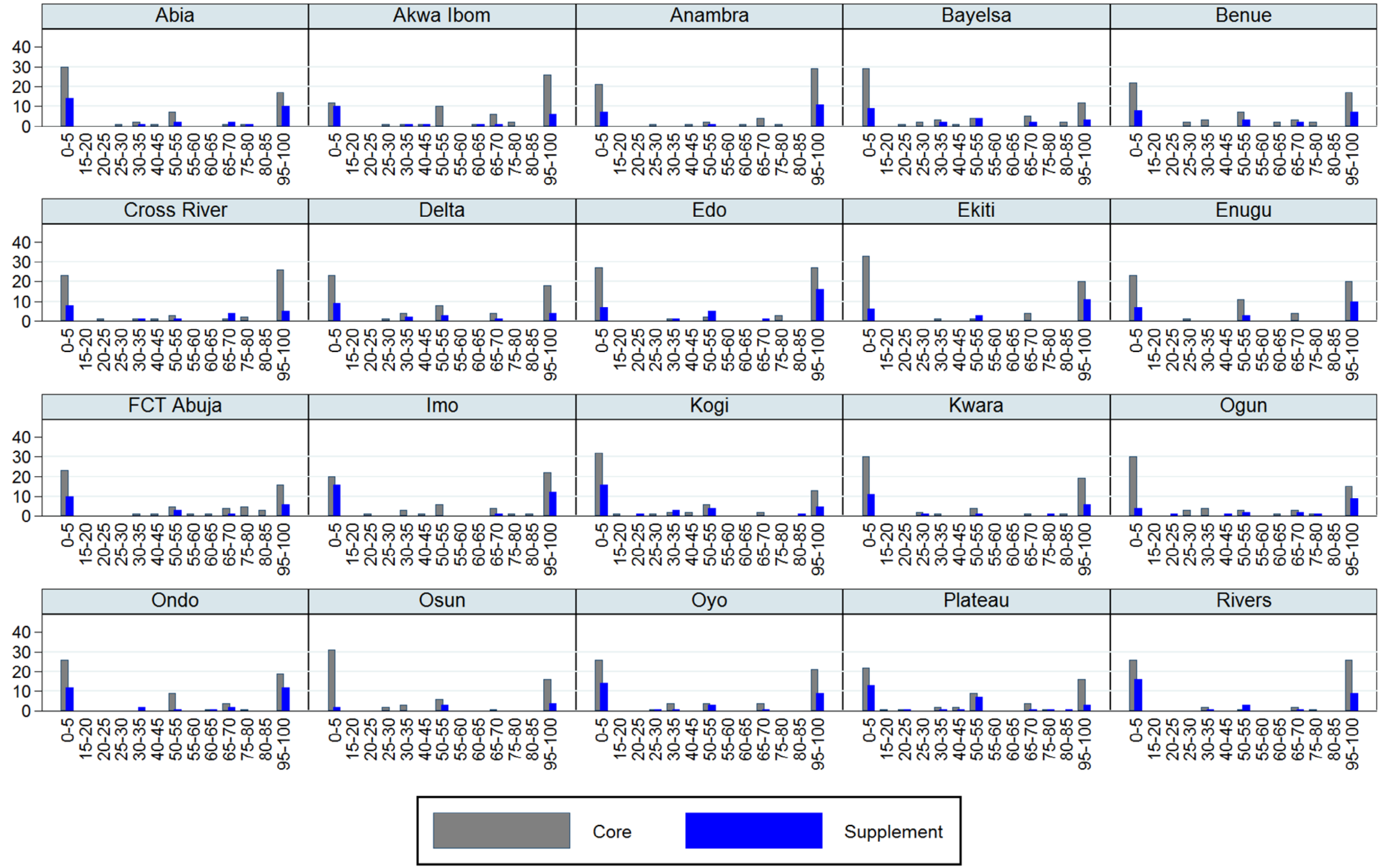

Y-axis is number of clusters and x-axis is the outcome (% of Children 12-23m who Received Penta3)

Differences

- The next figure shows the observed differences (core % - supplementary %) sorted from smallest to largest
- Under the null hypothesis of no systematic difference in bias, we would expect to see the differences distributed fairly equally on both sides of zero, but this is a random process so we may not see precisely 10 differences that are > zero and ten that are < zero
- All three outcomes are distributed quite evenly around zero
- The most notable outliers are in the first panel for FCT Abuja and the second panel for Kwara

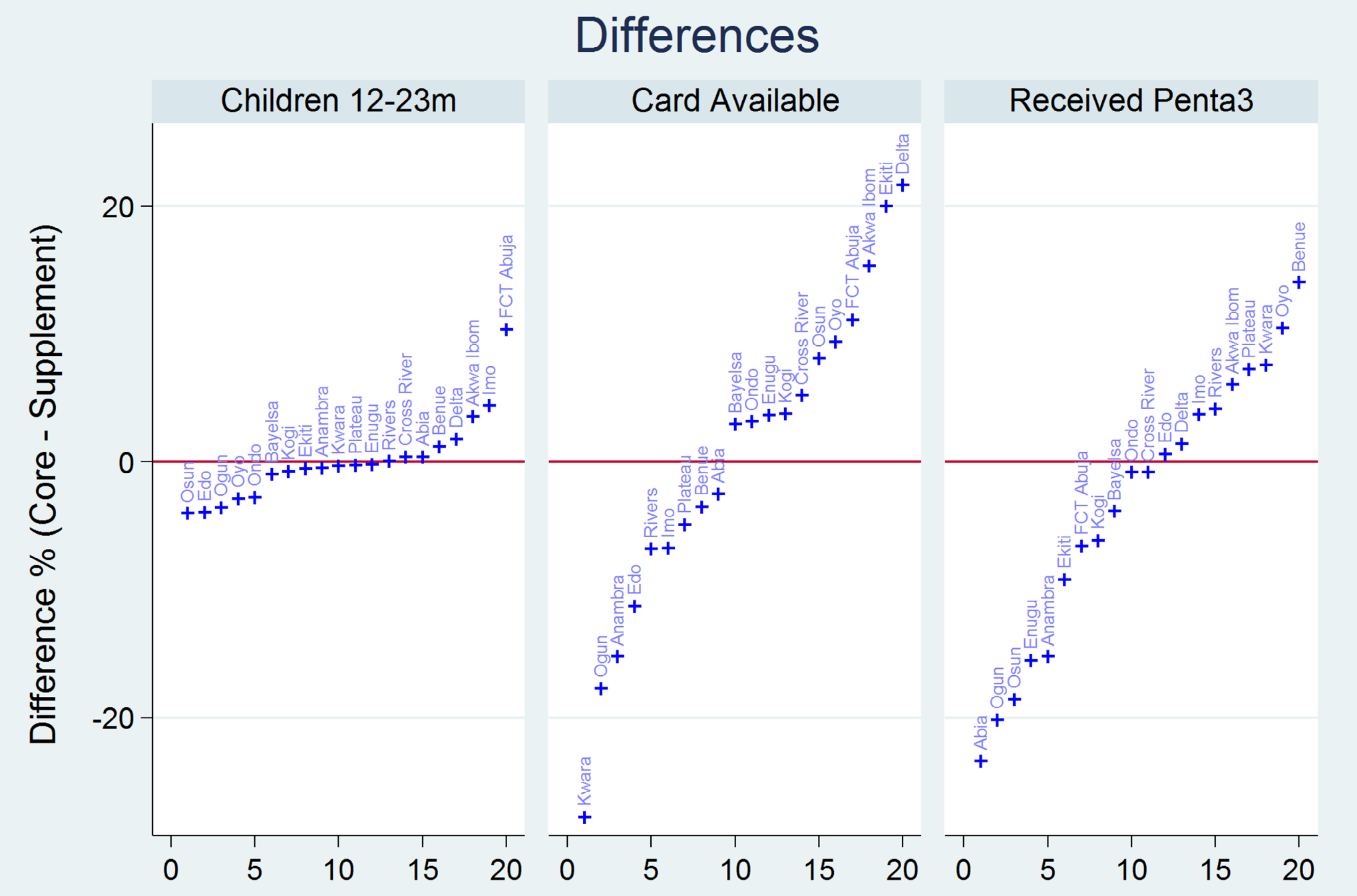

Differences
Children 12-23m
Card Available
Received Penta3
Difference % (Core - Supplement)
20
0
-20
0
5
10
15
20
Osun
Edo
Ogun
Oyo
Ondo
Bayelsa
Kogi
Ekiti
Anambra
Kwara
Plateau
Enugu
Rivers
Cross River
Abia
Benue
Delta
Akwa Ibom
Imo
FCT Abuja
Kwara
Ogun
Anambra
Edo
Rivers
Imo
Plateau
Benue
Abia
Bayelsa
Ondo
Enugu
Kogi
Cross River
Osun
Oyo
FCT Abuja
Akwa Ibom
Ekiti
Delta
Abia
Ogun
Osun
Enugu
Anambra
Ekiti
FCT Abuja
Kogi
Bayelsa
Ondo
Cross River
Edo
Delta
Imo
Rivers
Akwa Ibom
Plateau
Kwara
Oyo
Benue

P-values

- The next slide shows three panels of unadjusted p-values, sorted from smallest to largest
- States with the largest observed differences on the earlier slide will have the smallest p-values on the next slide
- Under the null hypothesis we expect these to be quite evenly distributed between 0 and 1

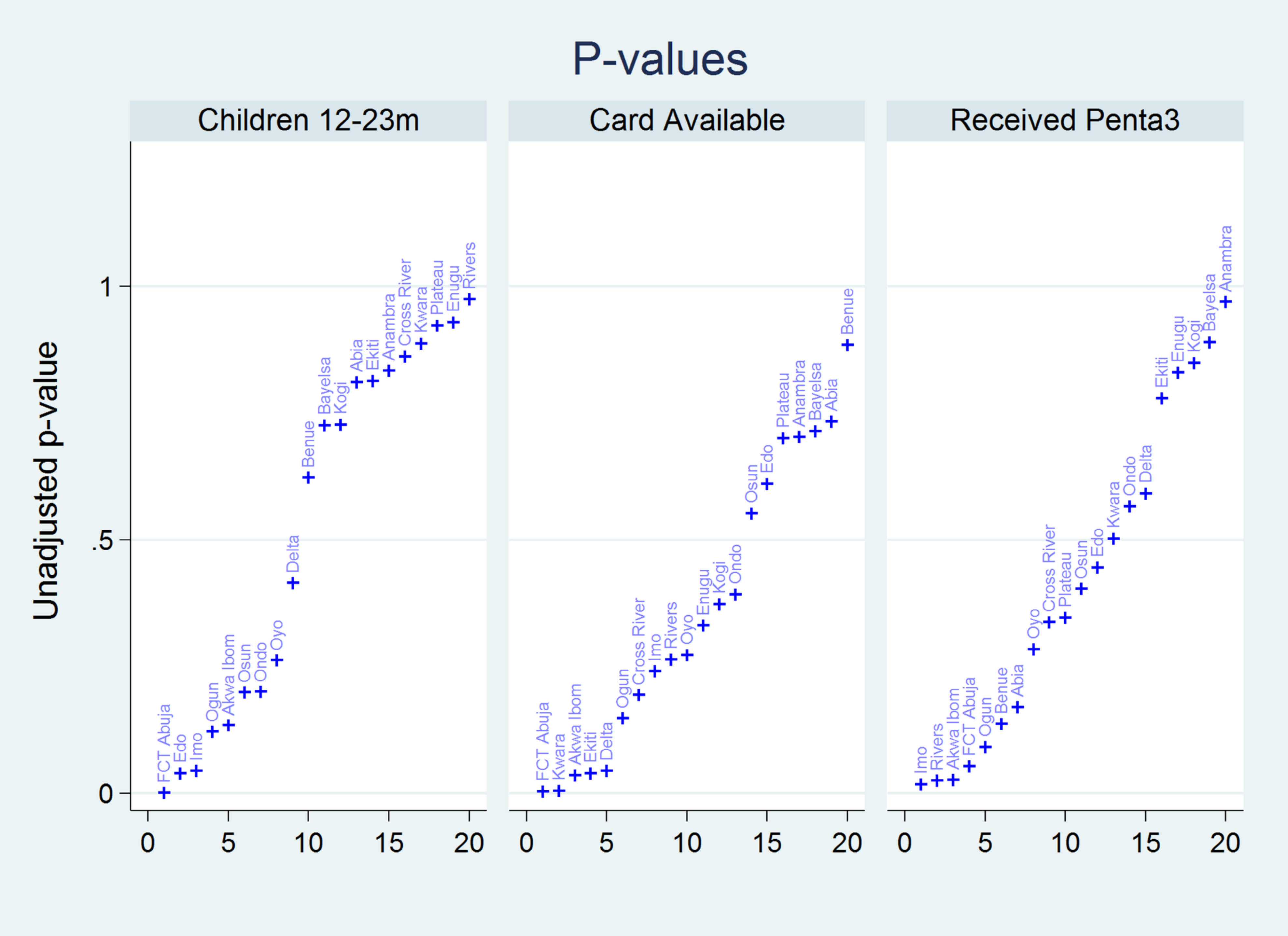
P-values
Children 12-23m
Card Available
Received Penta3
Unadjusted p-value
0
.5
1
0
5
10
15
20
FCT Abuja
Edo
Imo
Ogun
Akwa Ibom
Osun
Ondo
Oyo
Delta
Benue
Bayelsa
Kogi
Abia
Ekiti
Anambra
Cross River
Kwara
Plateau
Enugu
Rivers

Random Permutation Test Histograms

- Methods:
  - Within a single state, let the number of core MICS clusters be denoted N_{core} and the number of supplementary clusters be denoted N_{supp}
  - The null hypothesis is that the observed difference (core – supplementary) is a random draw from the distribution of all possible differences when clusters are split into two groups of N_{core} and N_{supp} clusters
  - The number of possible permutations is too high here to calculate all possible differences, so we take a sample
  - The cluster labels 'core' and 'supplement' were re-assigned randomly 500,000 times and each time a new difference was calculated for each outcome (e.g., In a state with 60 core clusters and 30 supplementary clusters, the clusters were pooled and 30 were randomly selected and labeled 'supplement' while the remainder were labeled 'core' and then the differences were calculated as core – supplementary)
  - If the null is true, the observed difference is unlikely to fall in the extreme tails of the distribution of differences

Random Permutation Test Histograms

- Figures
  - The thin red vertical line appears at the observed difference
  - If the observed difference is positive, then the thin red line appears to the right of 0 and the core proportion is > the supplementary proportion
  - If negative, the core proportion is < the supplementary proportion
  - Green portions of the graph represent permutations where the absolute value of the permutation difference is > the absolute value of the observed difference
  - The more green ink in the figure, the more support for the null hypothesis
  - **The unadjusted p-value is the proportion of permutations that are green**
  - Red portions represent permutations where the absolute value of the difference is < the absolute value of the observed difference
  - Each figure lists the observed core & supplementary coverage at the top of the histogram

Abia

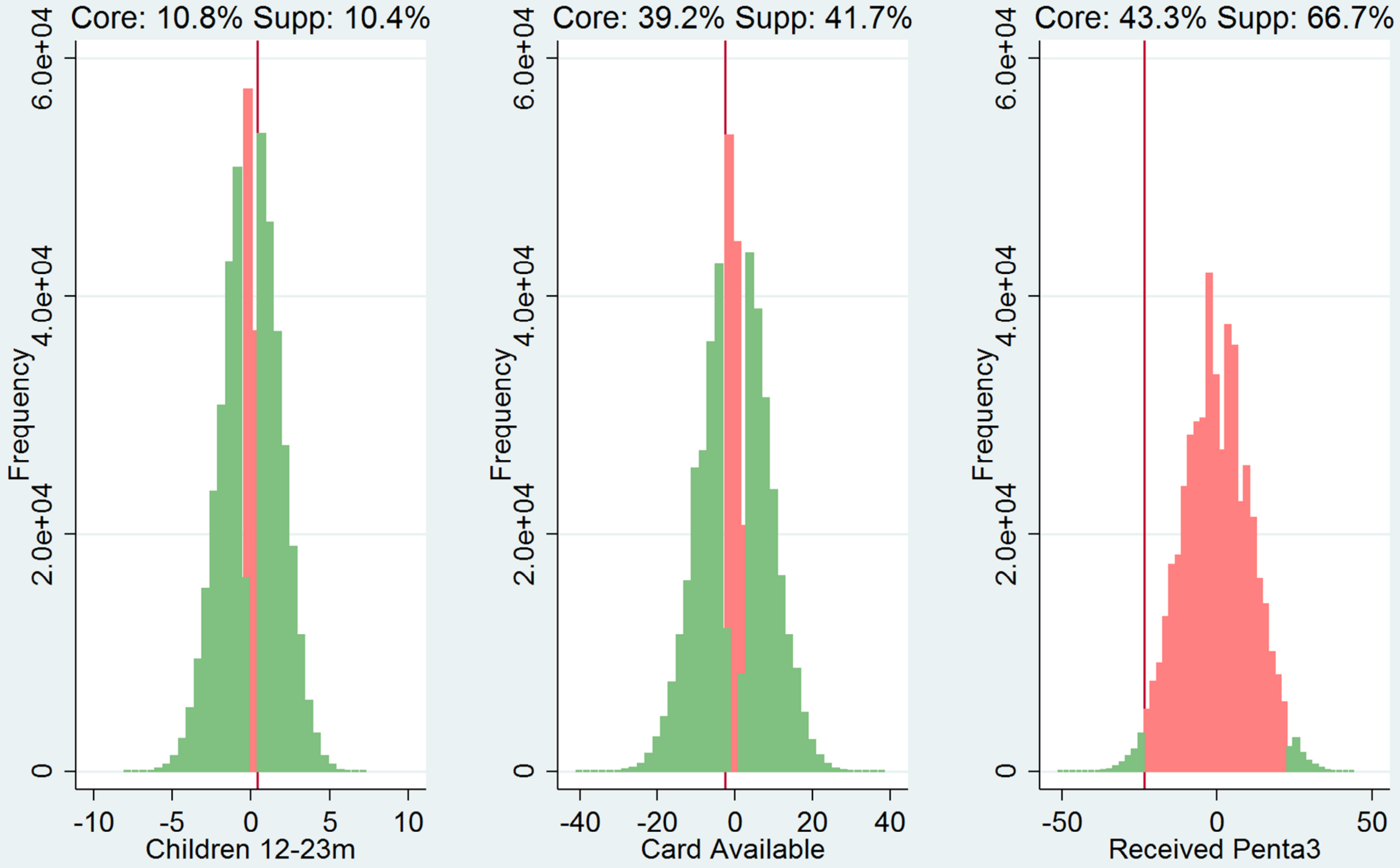

Histograms of all combinations of differences

Akwa Ibom

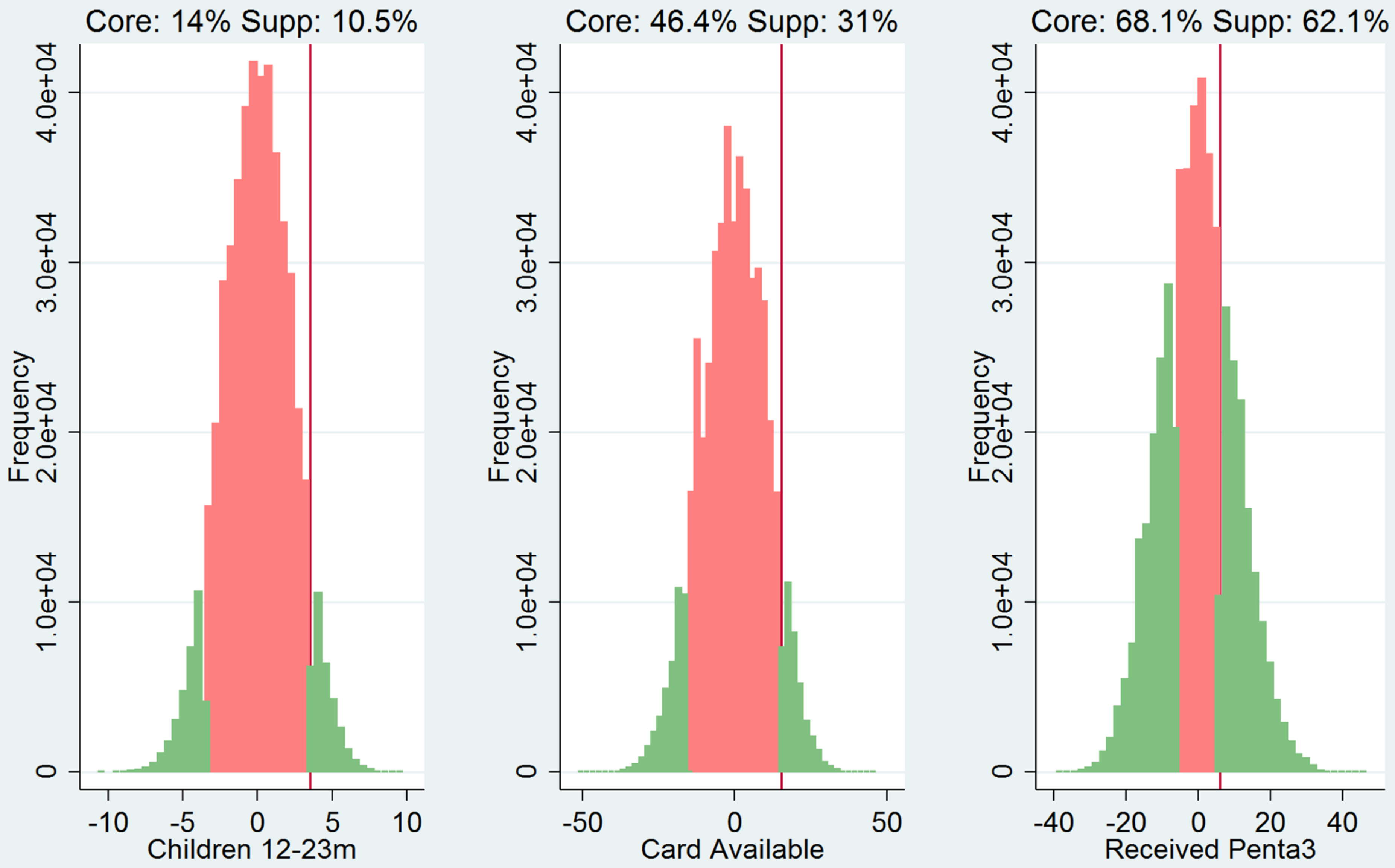

Histograms of all combinations of differences

Anambra

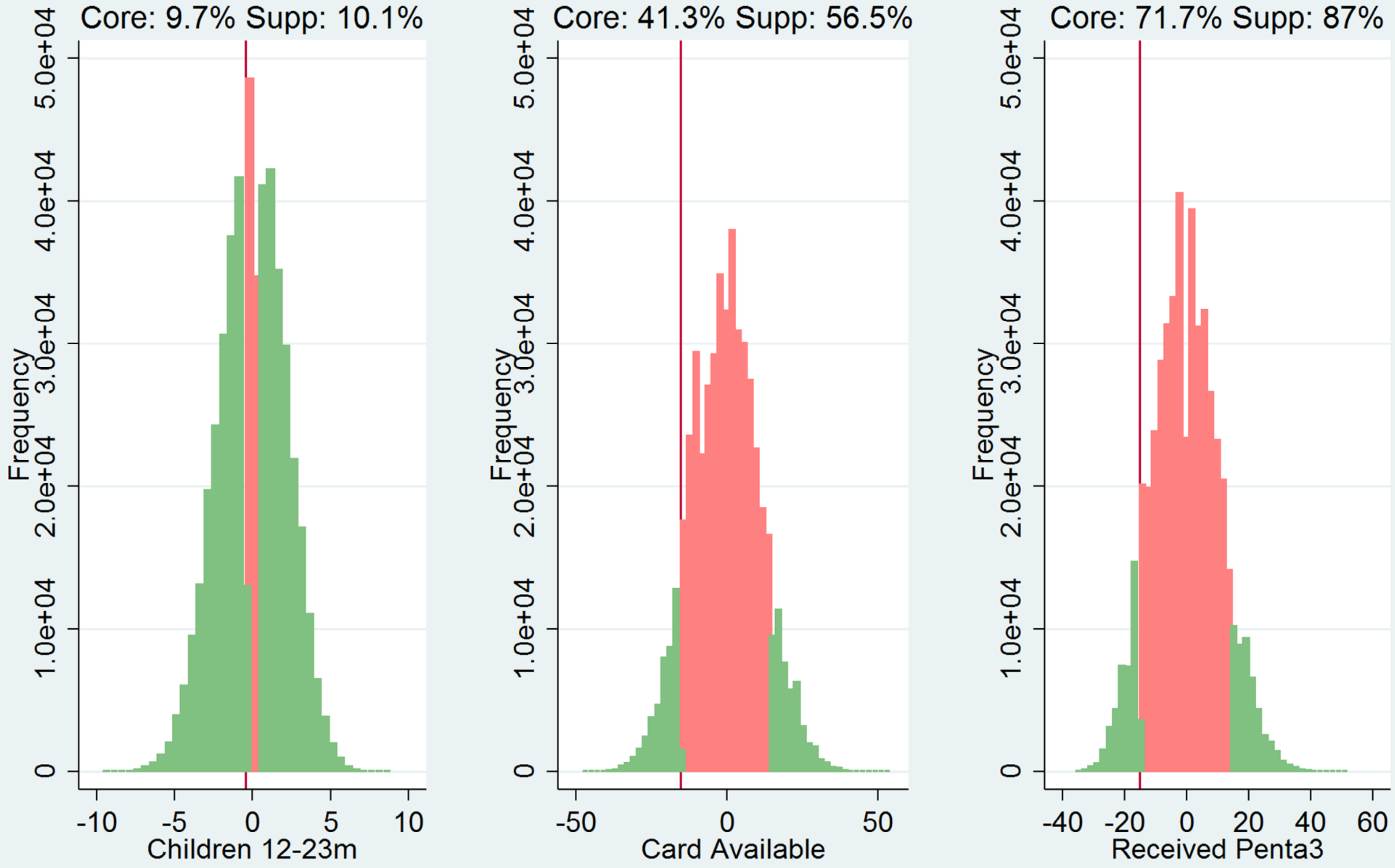

Histograms of all combinations of differences

Bayelsa

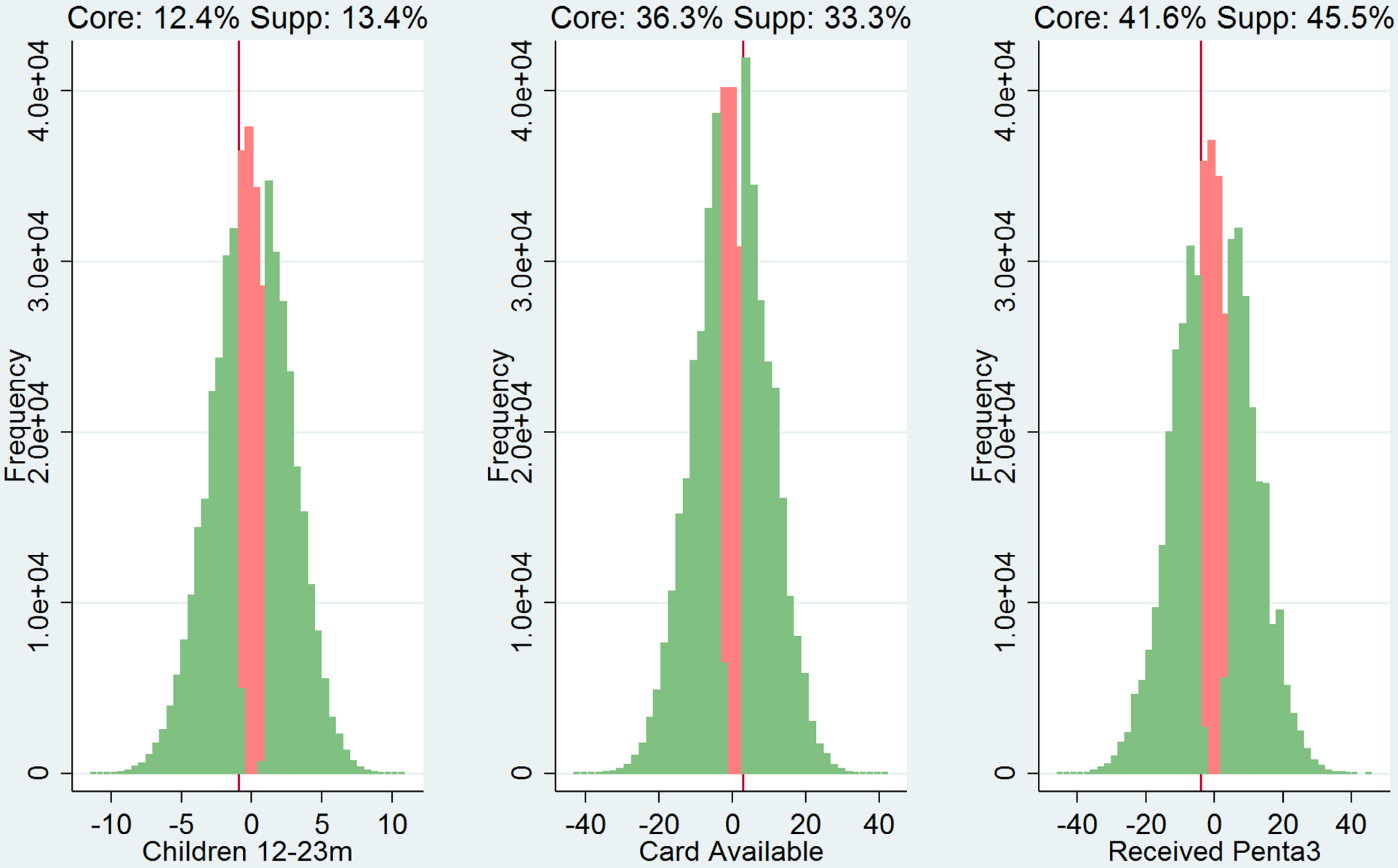

Histograms of all combinations of differences

Benue

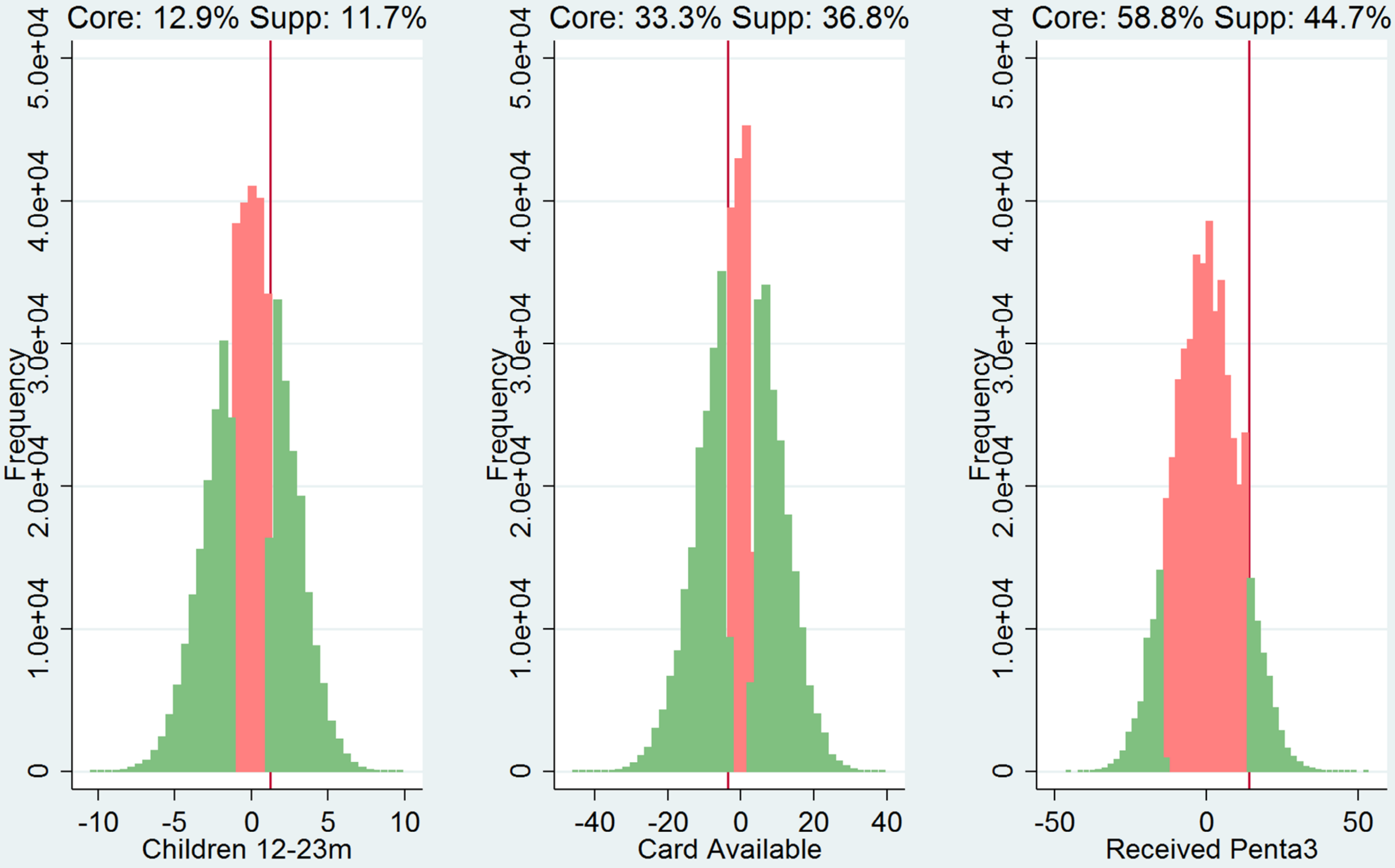

Histograms of all combinations of differences

Cross River

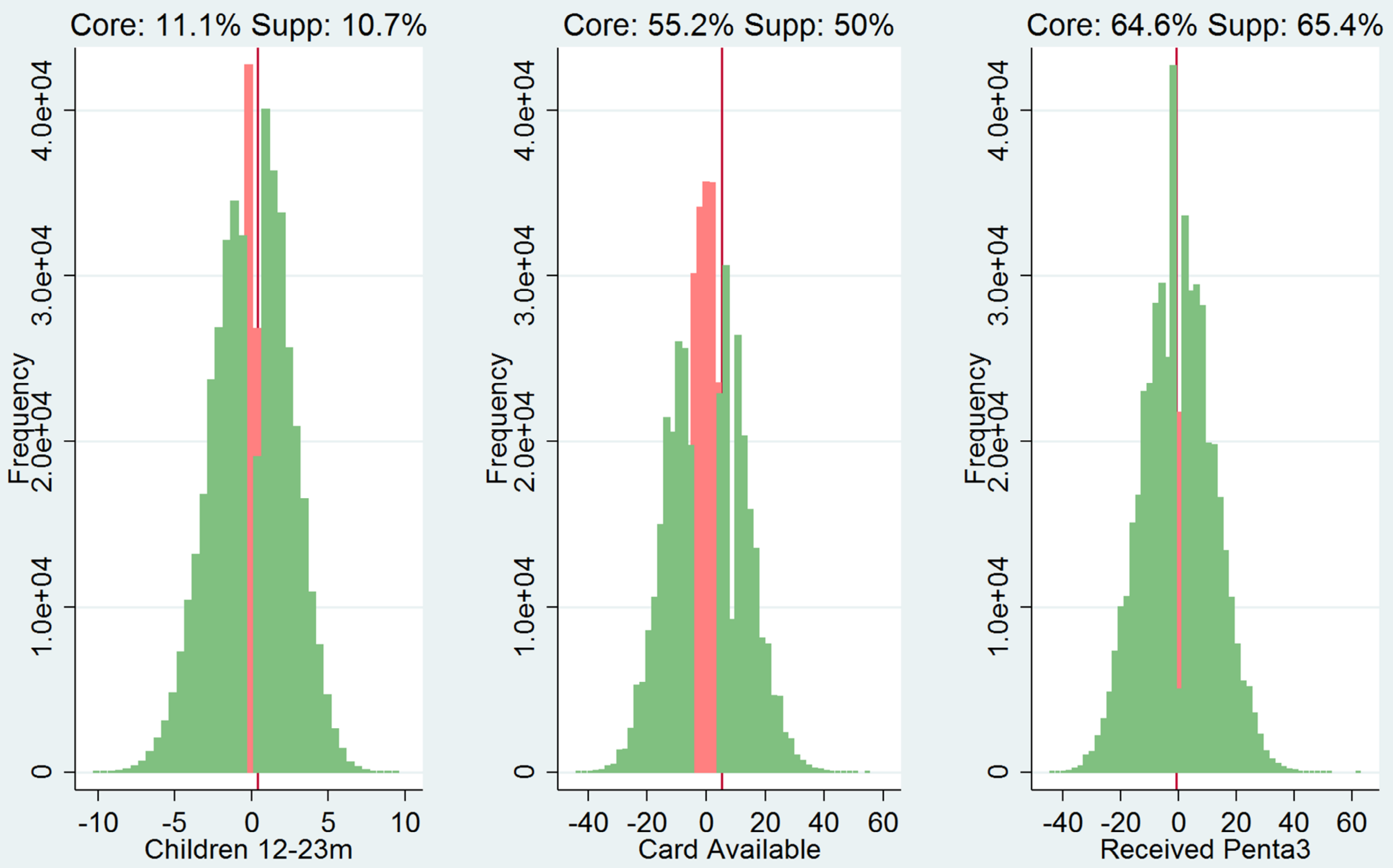

Histograms of all combinations of differences

Delta

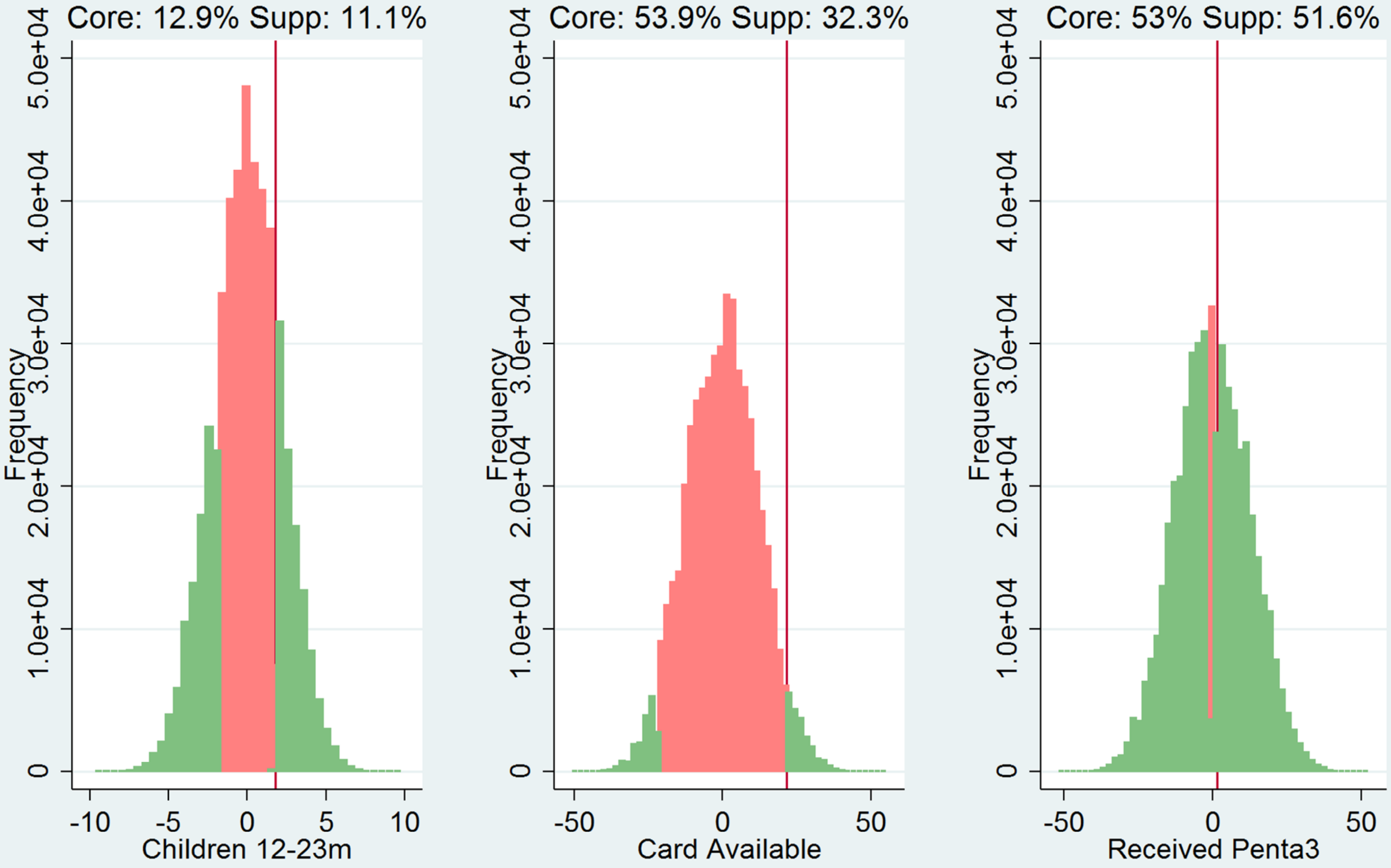

Histograms of all combinations of differences

Edo

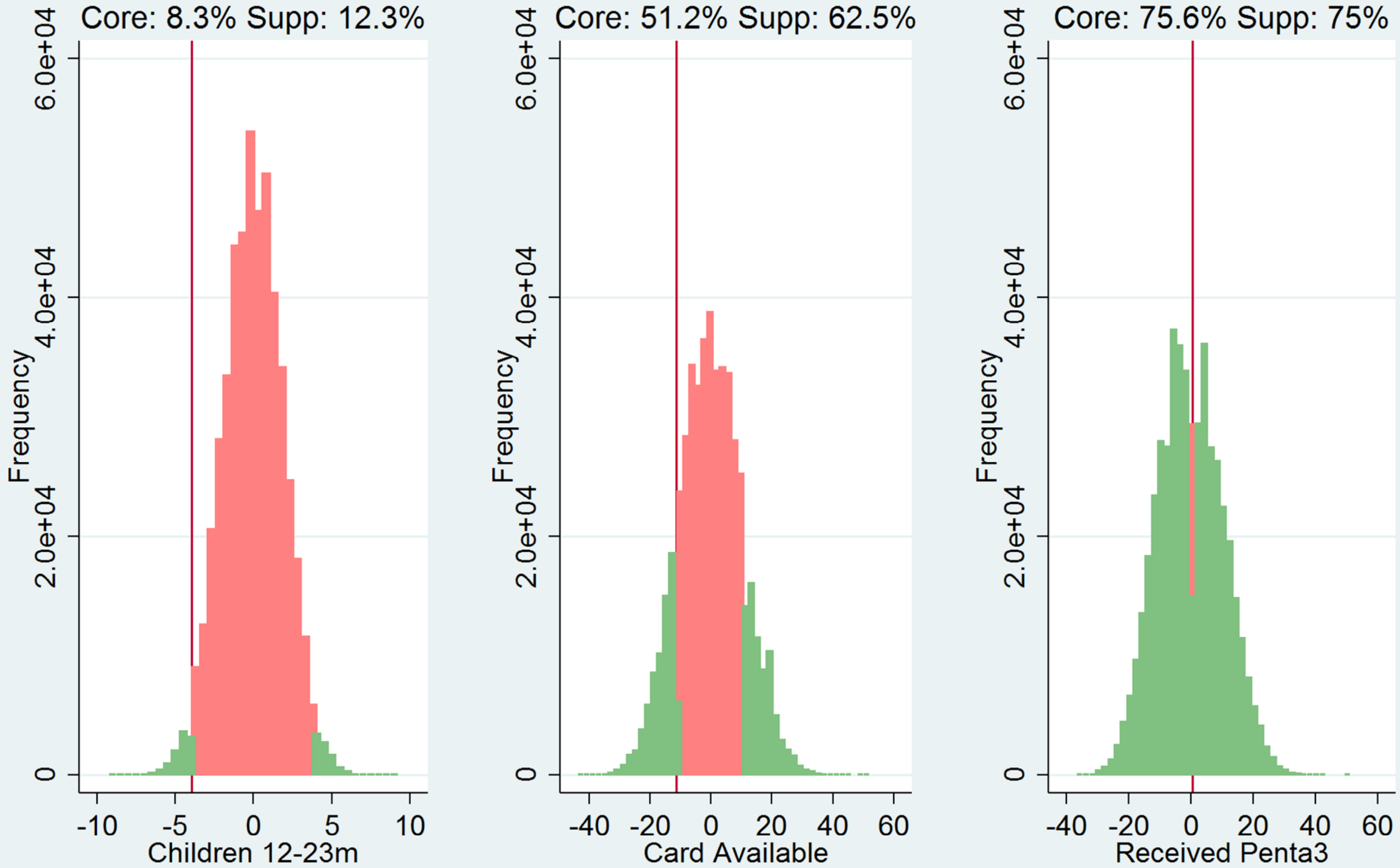

Histograms of all combinations of differences

Ekiti

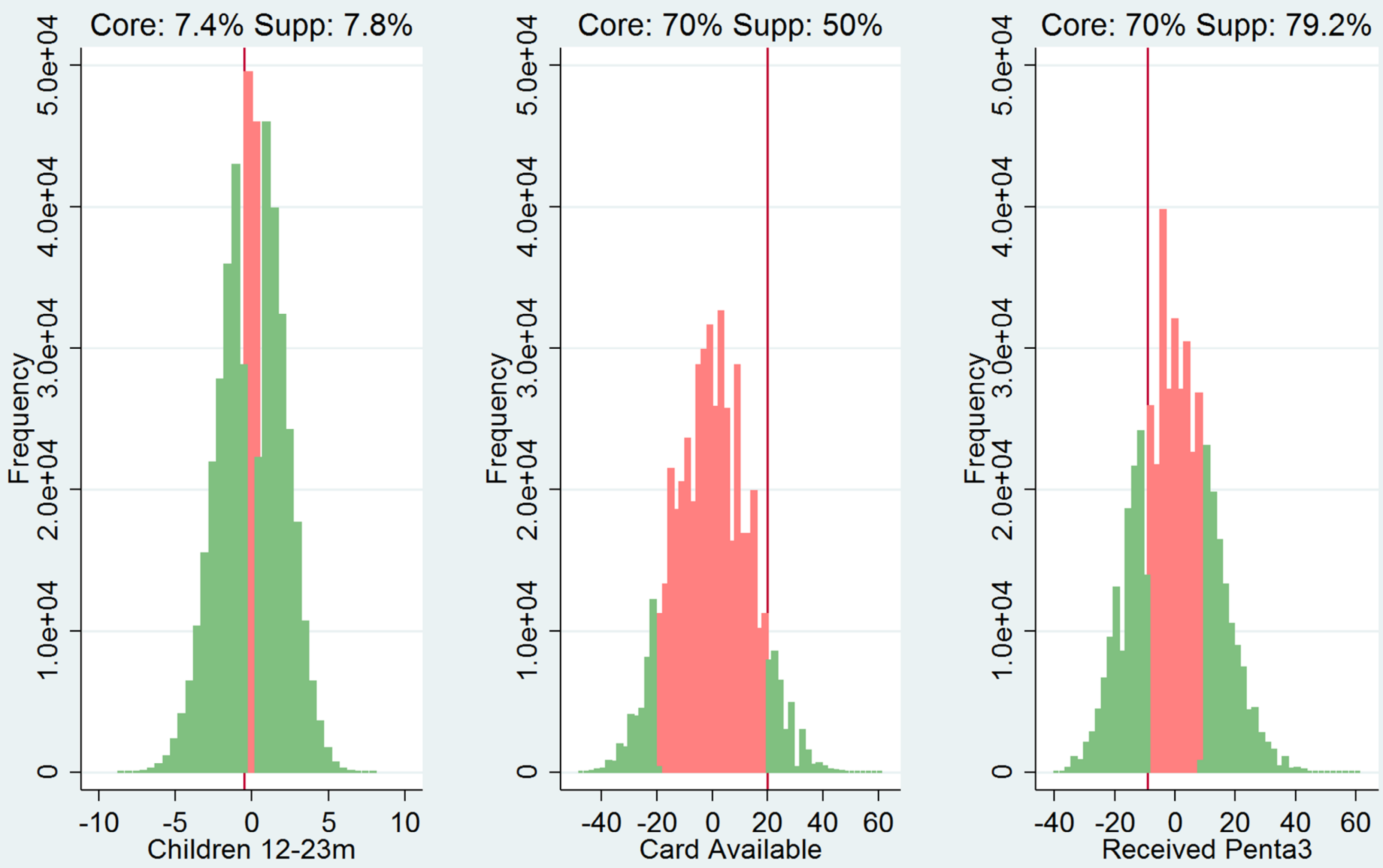

Histograms of all combinations of differences

Enugu

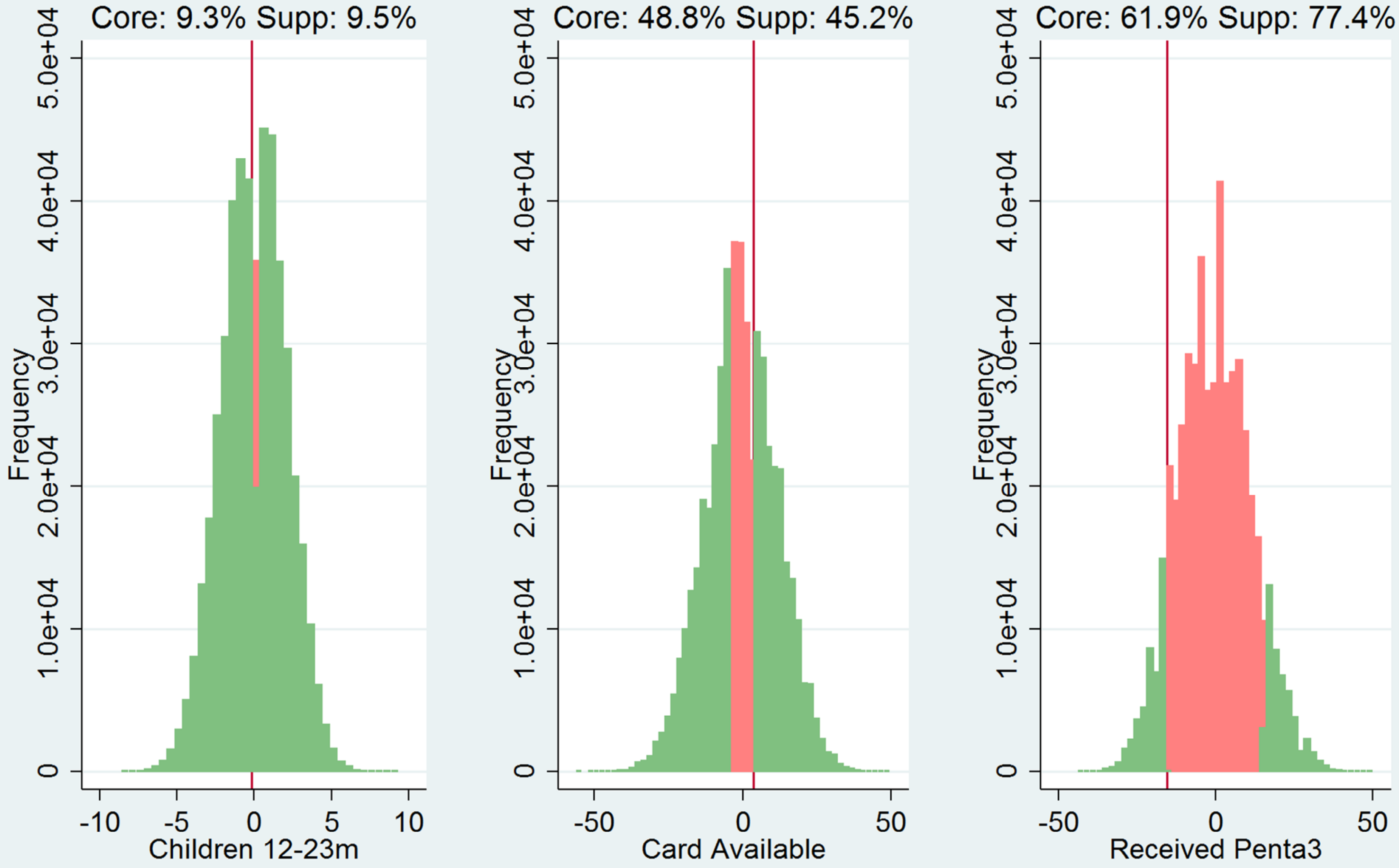

Histograms of all combinations of differences

FCT Abuja

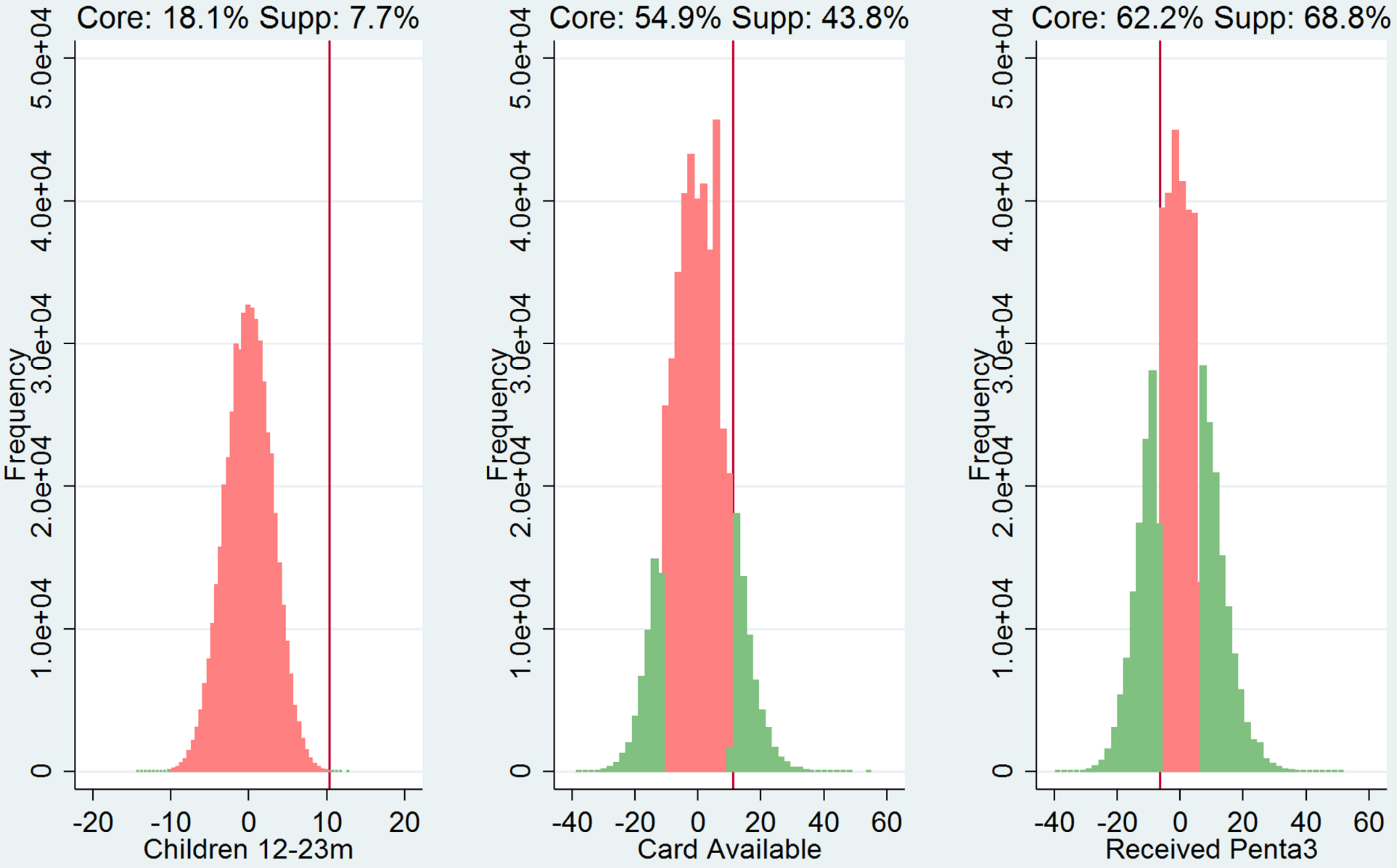

Histograms of all combinations of differences

Imo

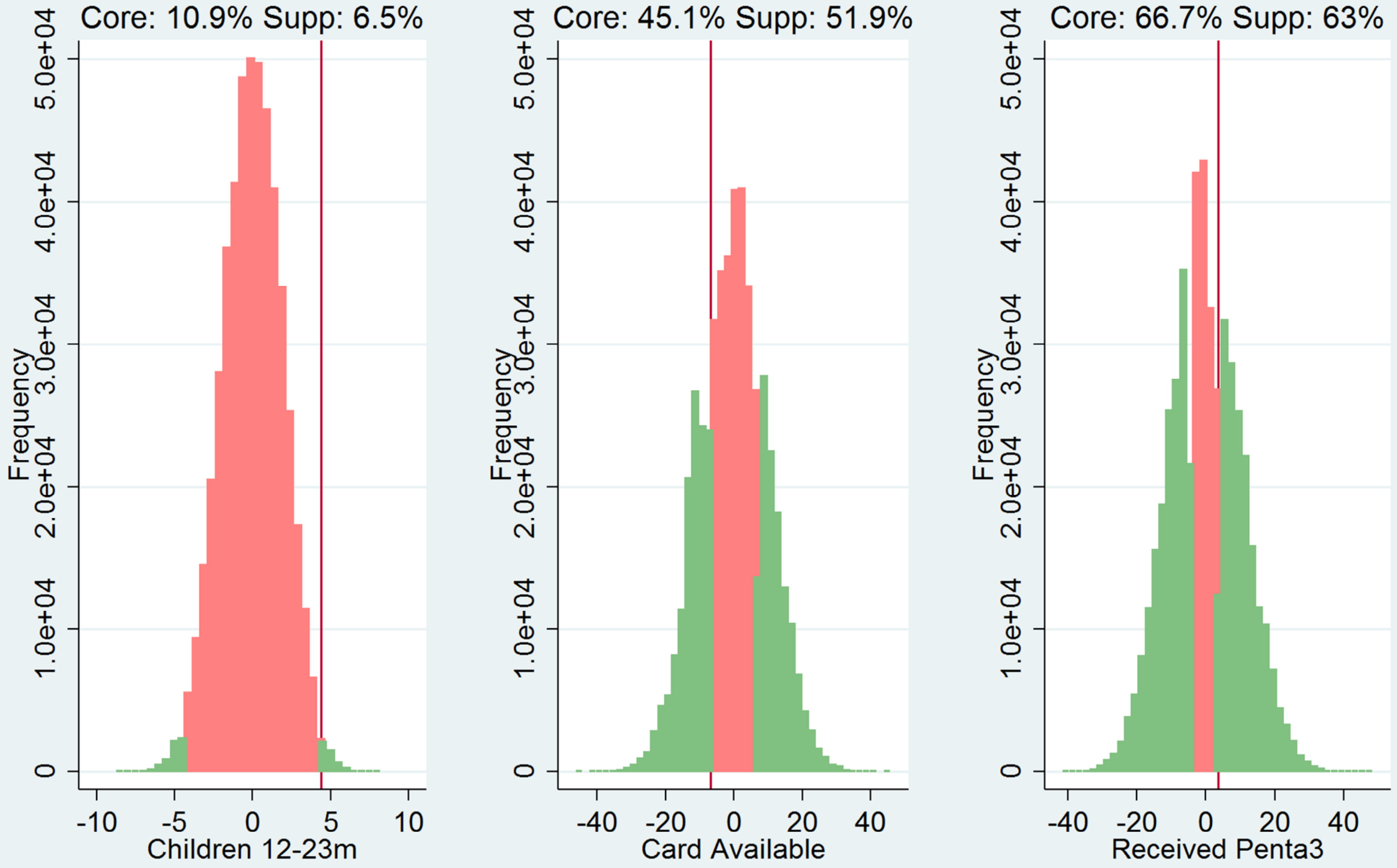

Histograms of all combinations of differences

Kogi

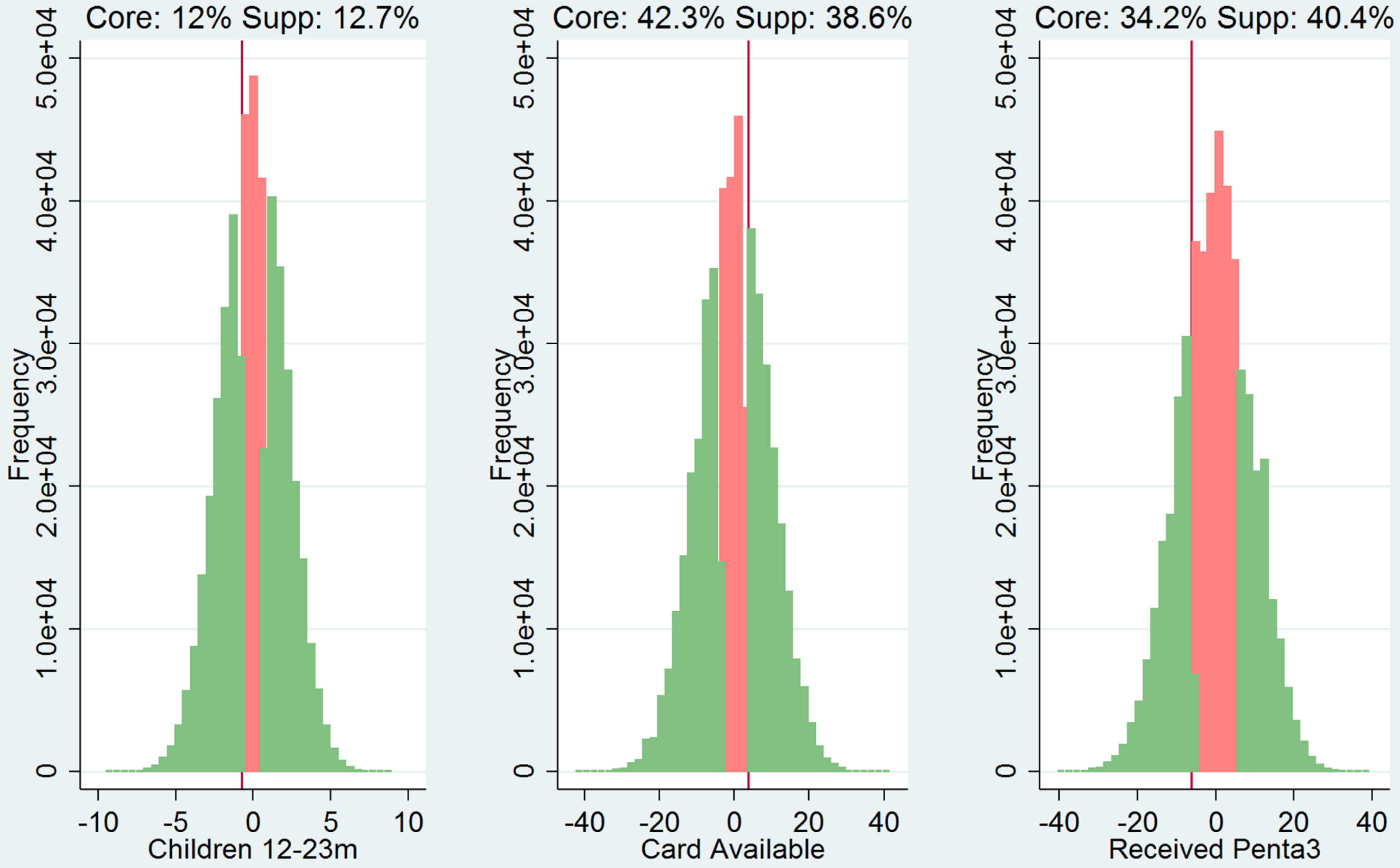

Histograms of all combinations of differences

Kwara

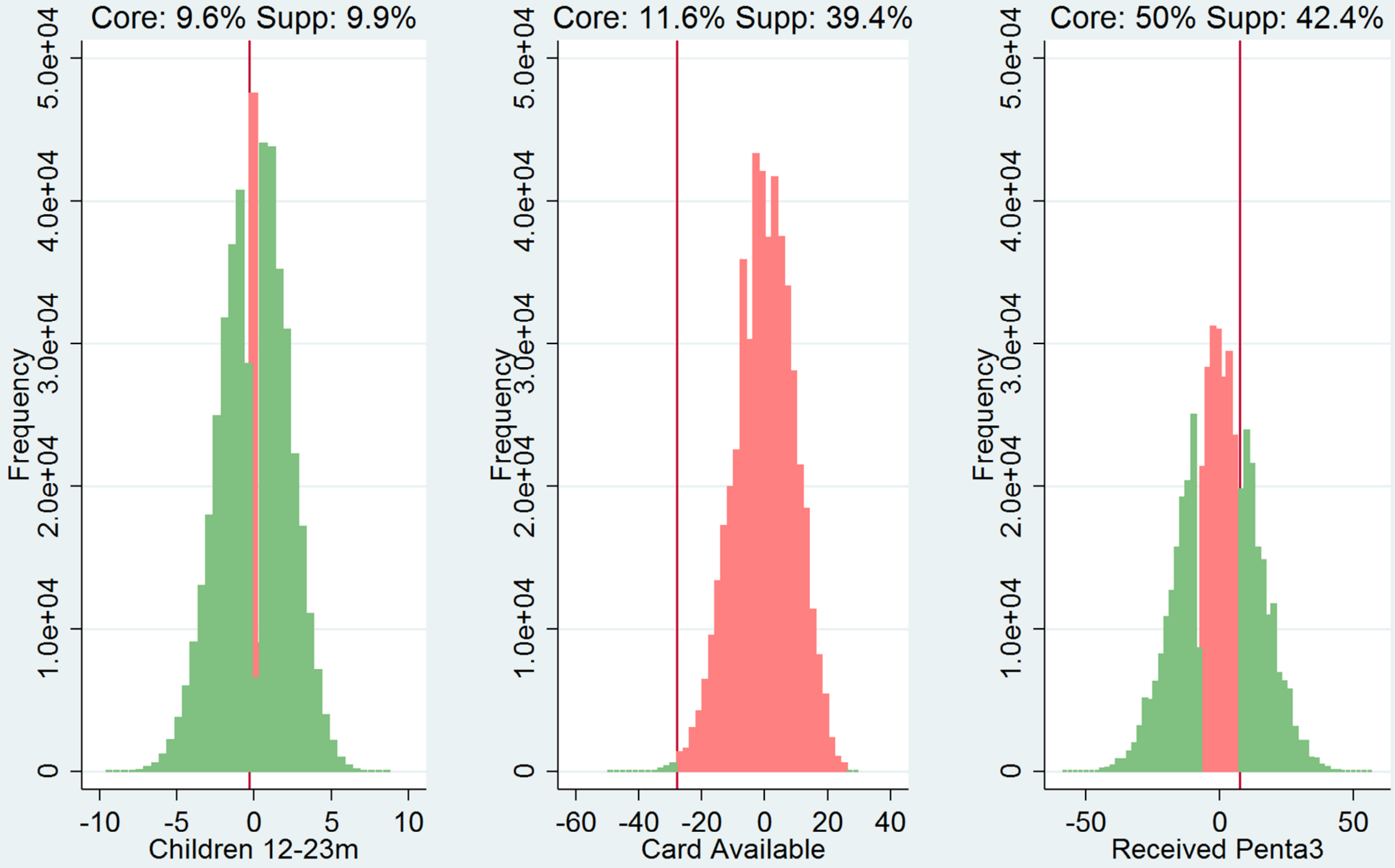

Histograms of all combinations of differences

Ogun

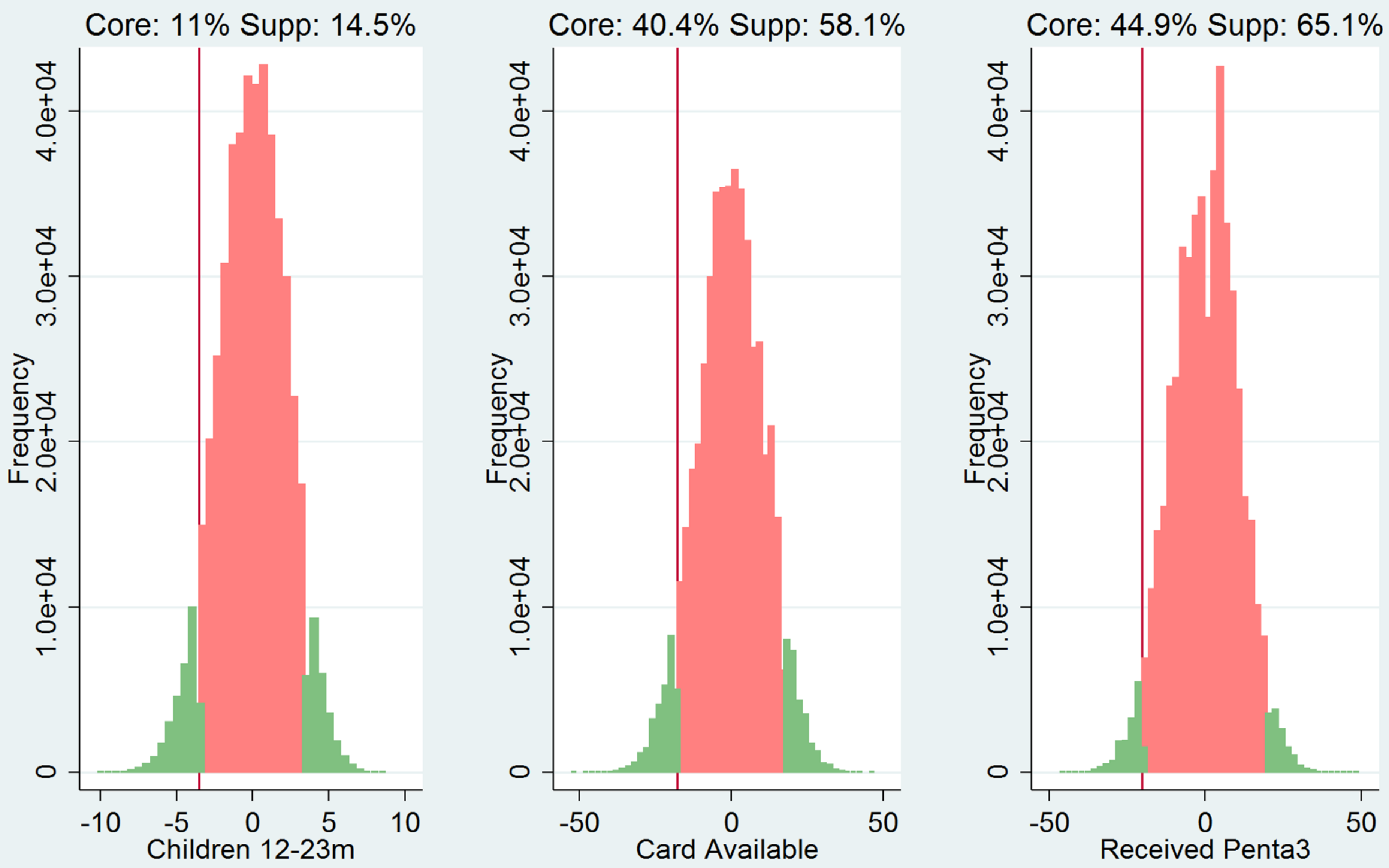

Histograms of all combinations of differences

Ondo

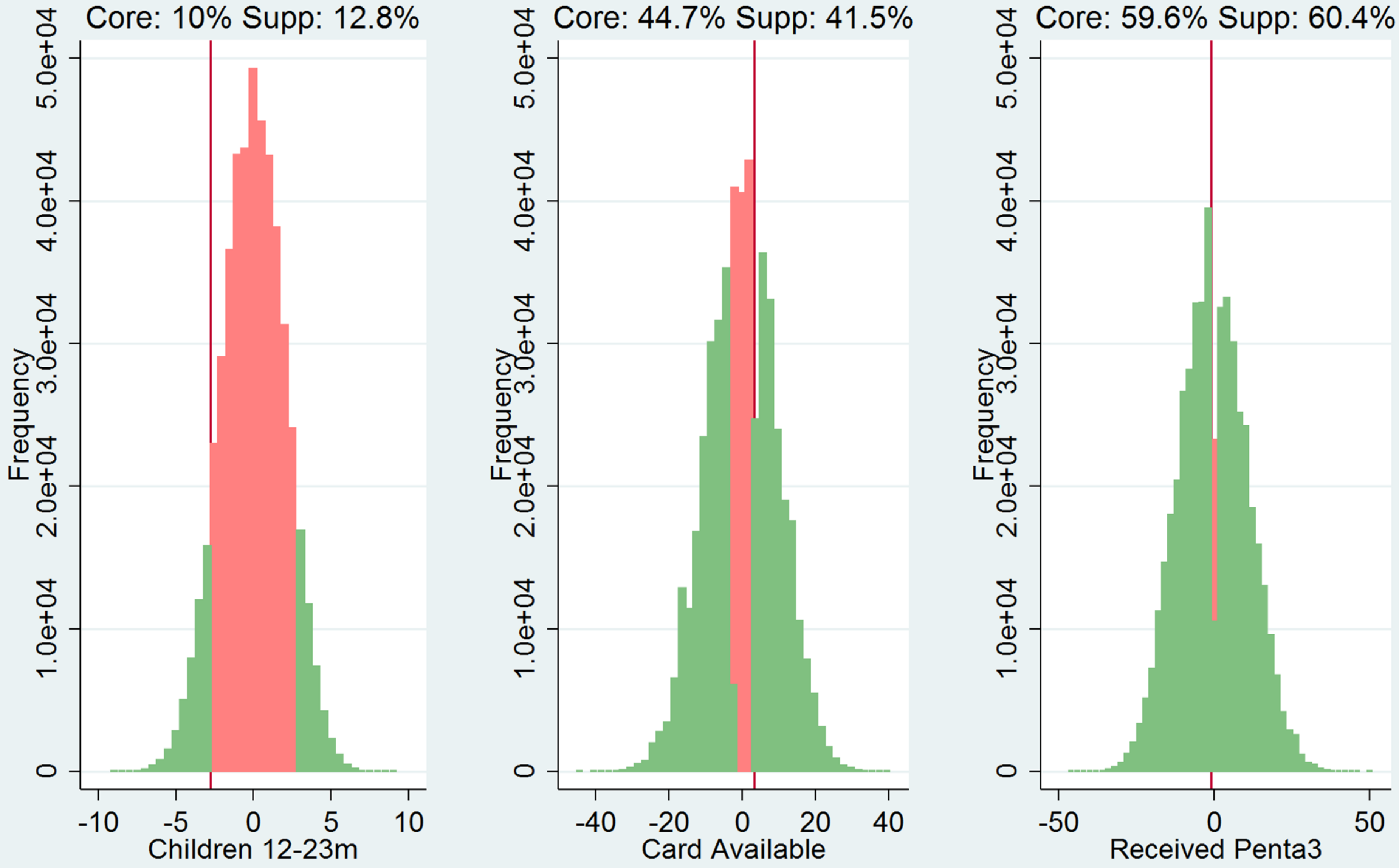

Histograms of all combinations of differences

Osun

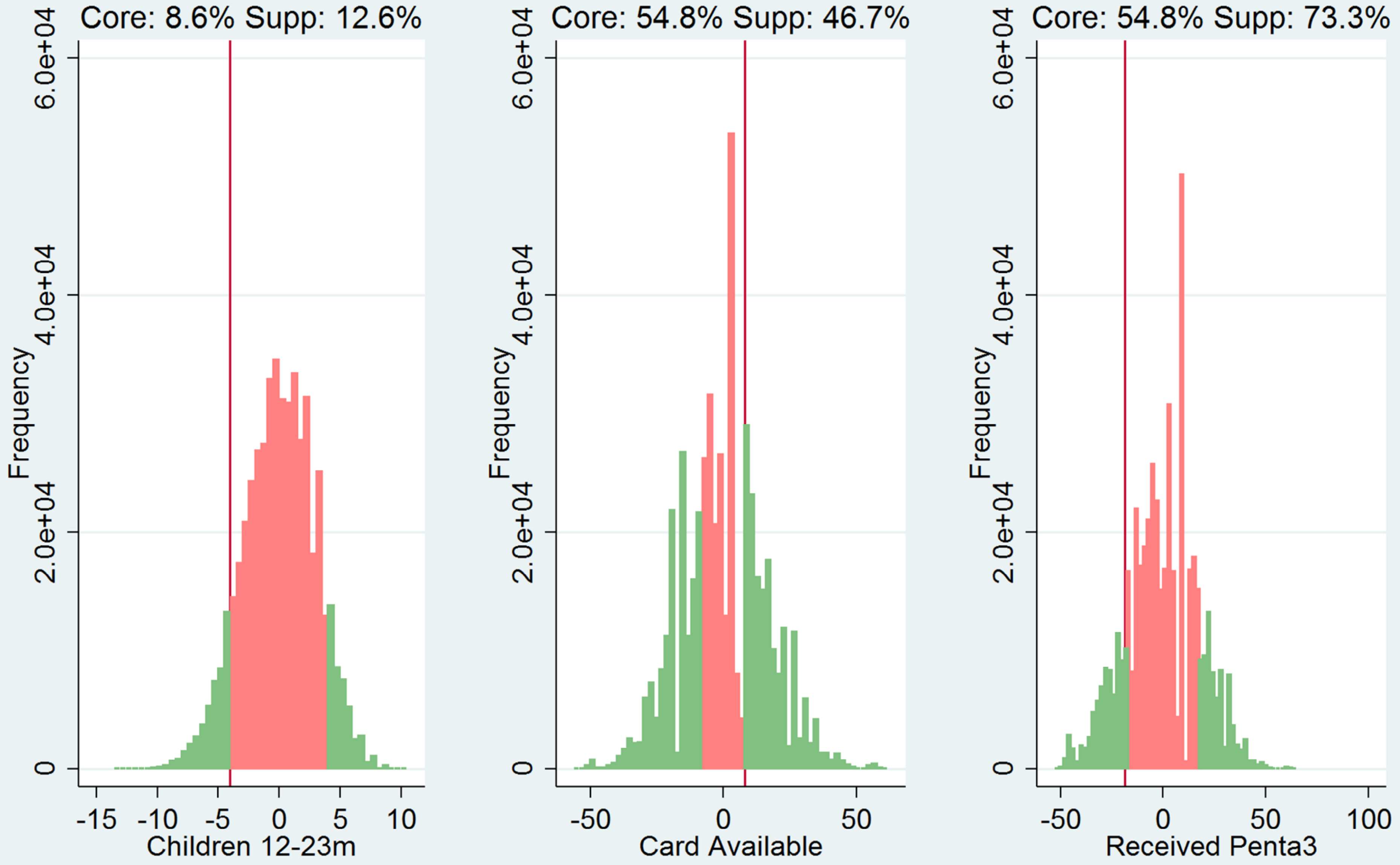

Histograms of all combinations of differences

Oyo

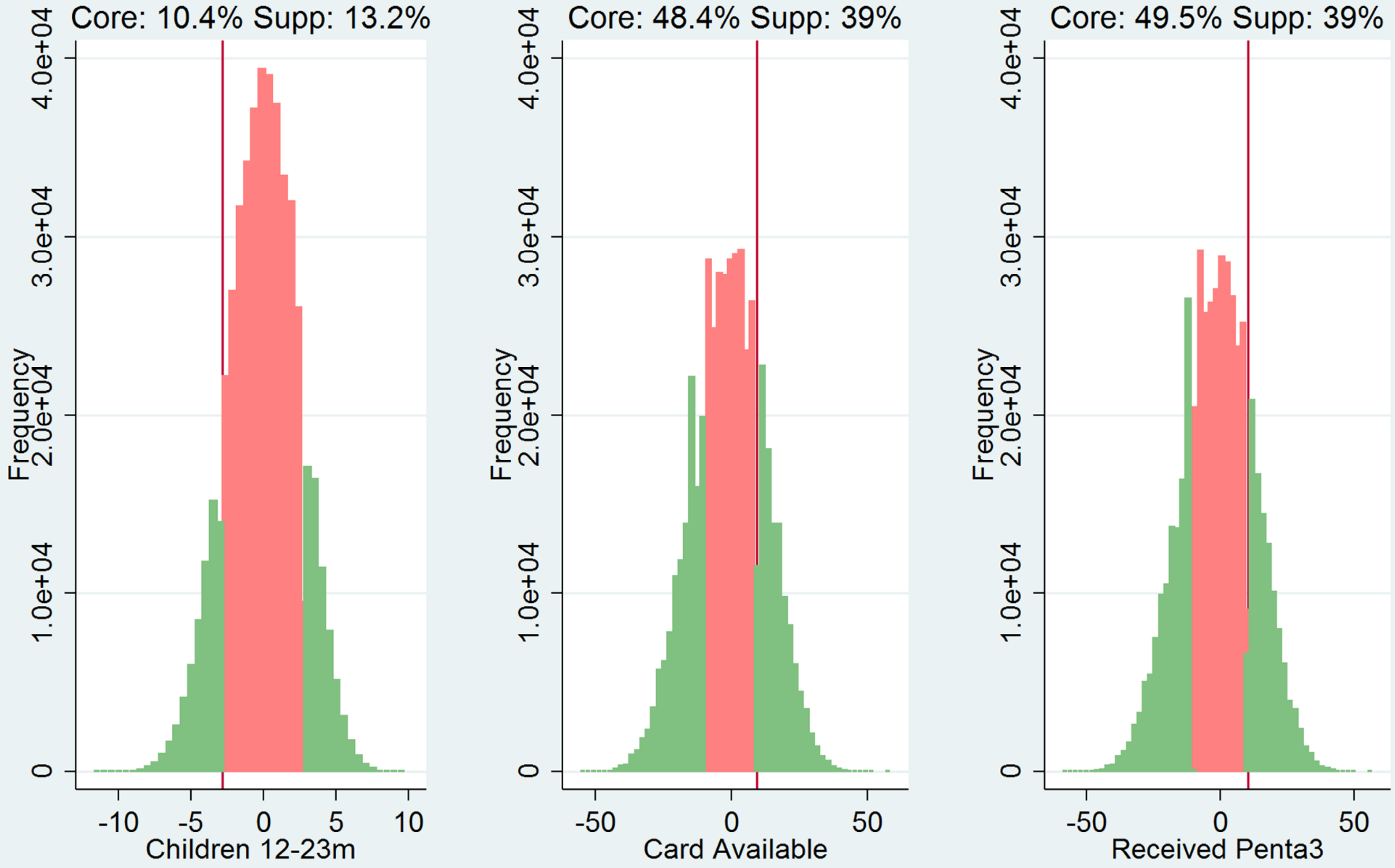

Histograms of all combinations of differences

Plateau

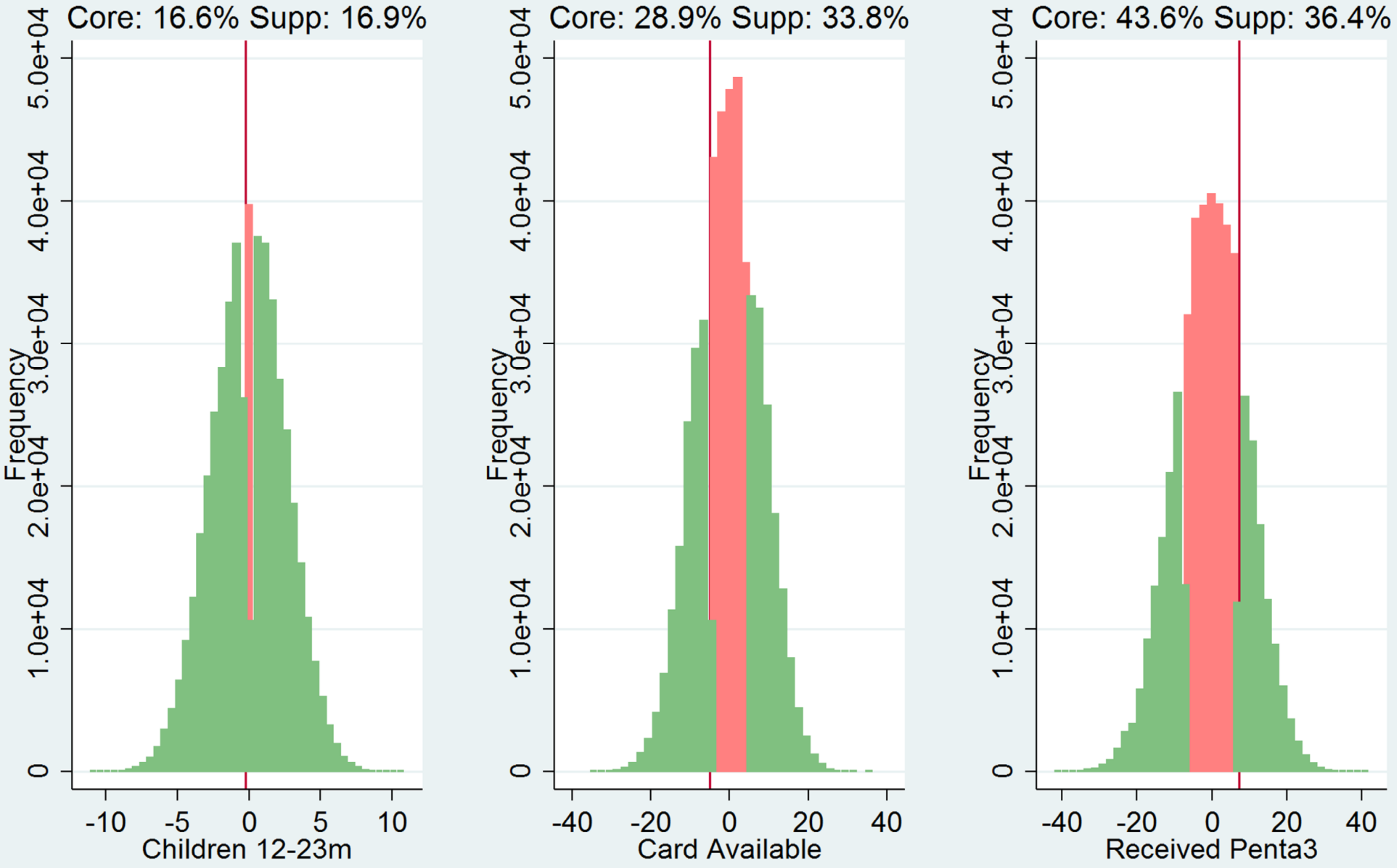

Histograms of all combinations of differences

Rivers

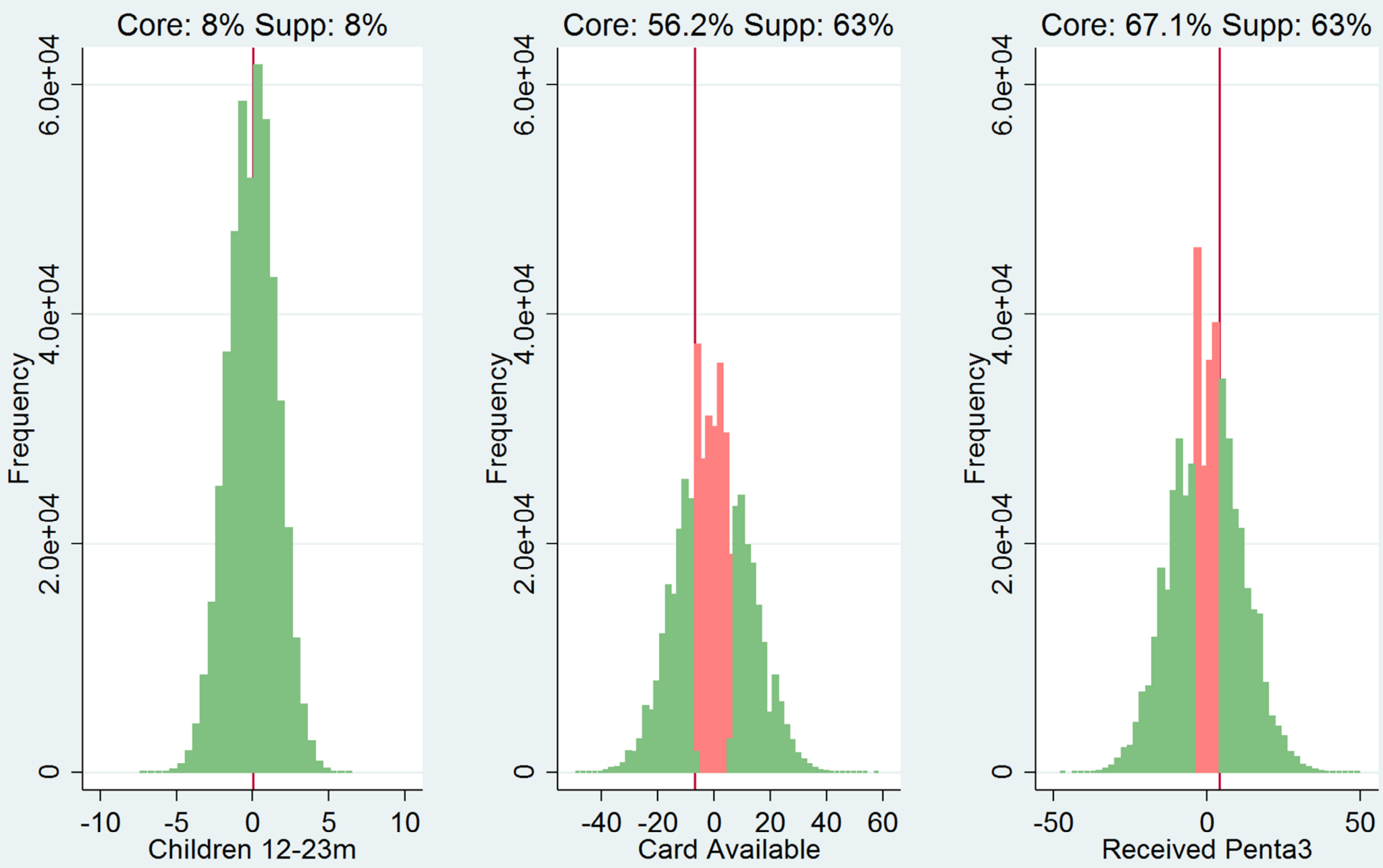

Histograms of all combinations of differences

Adjustment for Multiple Comparisons

- Recall that we want to limit the family-wise probability of Type I error to be ≤ 1%
- The process Holm-Bonferroni procedure first sorts all 60 tests, with the smallest unadjusted p-value at the top of the list
- Now calculate *adjusted p-values* thus: Look at the smallest unadjusted p-value; multiply its value by 60; if the result is ≤ 0.01 then the data are declared "not poolable" for that state and we move on to examine the next smallest p-value, using a multiplier of 59 rather than 60 (decreasing the multiplier by 1 for each prior adjusted result that is ≤ 0.01); if any adjusted p-value is > 0.01 then the data for all remaining tests are declared to be "poolable" and the comparison procedure is halted
- **In this dataset all adjusted p-values were > 0.01, so the data are declared to be "poolable" in every state**
- (Only two unadjusted p-values were < 0.01: one for FCT Abuja and one for Kwara, but all were much larger than 0.01 after adjustment)

Poolability Testing: Observed Differences & Unadjusted P-values

	Number of clusters		% HH with respondents 12-23m				Unweighted % 12-23m olds with cards with 1+ dates				Unweighted % of 12-23m olds with evidence of Penta3			
	MICS	NICS	MICS	NICS	Difference	Unadjusted p-value	MICS	NICS	Difference	Unadjusted p-value	MICS	NICS	Difference	Unadjusted p-value
	N	N	%	%	%		%	%	%		%	%	%	
**State**														
North Central														
FCT-Abuja	60	20	18	8	10	0.00041	55	44	11	0.22907	62	69	-7	0.48421
Benue	58	20	13	12	1	0.62208	33	37	-4	0.72281	59	45	14	0.19976
Kogi	59	30	12	13	-1	0.72722	42	39	4	0.69212	34	40	-6	0.52371
Kwara	58	20	10	10	0	0.88689	12	39	-28	0.00293	50	42	8	0.59002
Plateau	58	29	17	17	0	0.92240	29	34	-5	0.55719	44	36	7	0.45637
South East														
Abia	60	30	11	10	0	0.81107	39	42	-2	0.77211	43	67	-23	0.03452
Anambra	60	19	10	10	0	0.83346	41	57	-15	0.19746	72	87	-15	0.18802
Enugu	59	20	9	10	0	0.92874	49	45	4	0.75658	62	77	-16	0.18846
Imo	59	29	11	7	4	0.02196	45	52	-7	0.50876	67	63	4	0.71128
South South														
Akwa Ibom	60	20	14	10	4	0.13376	46	31	15	0.16347	68	62	6	0.56117
Bayelsa	59	20	12	13	-1	0.72561	36	33	3	0.77762	42	45	-4	0.73774
Cross River	58	19	11	11	0	0.86140	55	50	5	0.67084	65	65	-1	0.95652
Delta	58	19	13	11	2	0.41423	54	32	22	0.07994	53	52	1	0.92309
Edo	60	30	8	12	-4	0.03950	51	63	-11	0.29740	76	75	1	0.94121
Rivers	58	30	8	8	0	0.97375	56	63	-7	0.57953	67	63	4	0.70458
South West														
Ekiti	59	20	7	8	0	0.81303	70	50	20	0.15489	70	79	-9	0.49297
Ogun	60	20	11	15	-4	0.12093	40	58	-18	0.11581	45	65	-20	0.05908
Ondo	60	30	10	13	-3	0.18310	45	42	3	0.74514	60	60	-1	0.95349
Osun	59	9	9	13	-4	0.18002	55	47	8	0.63168	55	73	-19	0.32545
Oyo	60	29	10	13	-3	0.26213	48	39	9	0.48758	49	39	10	0.45792

Notes:

a. Estimates are from pooled MICS and NICS survey datasets

b. All proprtions are unweighted in the poolability analysis

Poolability Testing: Holm-Bonferroni Adjustment for Multiple Comparisons & Adjusted P-values

Holm-Bonferroni Adjustment for MICS / NICS Poolability Analysis, Nigeria Combined MICS/NICS, 2016-17

P-value rank	State	Which Test?	Unadjusted p-value	Holm-Bonferroni Multiplier	Adjusted p-value	Difference significant? (Adjusted p-value <= 0.01?)
1	FCT-Abuja	Child	0.00041	60	0.02472	No
2	Kwara	Card	0.00293	59	0.17311	No
3	Imo	Child	0.02196	58	≥ 1.0	No
4	Abia	Penta3	0.03452	57	≥ 1.0	No
5	Edo	Child	0.03950	56	≥ 1.0	No
6	Ogun	Penta3	0.05908	55	≥ 1.0	No
7	Delta	Card	0.07994	54	≥ 1.0	No
8	Ogun	Card	0.11581	53	≥ 1.0	No
9	Ogun	Child	0.12093	52	≥ 1.0	No
10	Akwa Ibom	Child	0.13376	51	≥ 1.0	No
11	Ekiti	Card	0.15489	50	≥ 1.0	No
12	Akwa Ibom	Card	0.16347	49	≥ 1.0	No
13	Osun	Child	0.18002	48	≥ 1.0	No
14	Ondo	Child	0.18310	47	≥ 1.0	No
15	Anambra	Penta3	0.18802	46	≥ 1.0	No
16	Enugu	Penta3	0.18846	45	≥ 1.0	No
17	Anambra	Card	0.19746	44	≥ 1.0	No
18	Benue	Penta3	0.19976	43	≥ 1.0	No
19	FCT-Abuja	Card	0.22907	42	≥ 1.0	No
20	Oyo	Child	0.26213	41	≥ 1.0	No
21	Edo	Card	0.29740	40	≥ 1.0	No
22	Osun	Penta3	0.32545	39	≥ 1.0	No
23	Delta	Child	0.41423	38	≥ 1.0	No
24	Plateau	Penta3	0.45637	37	≥ 1.0	No
25	Oyo	Penta3	0.45792	36	≥ 1.0	No
26	FCT-Abuja	Penta3	0.48421	35	≥ 1.0	No
27	Oyo	Card	0.48758	34	≥ 1.0	No
28	Ekiti	Penta3	0.49297	33	≥ 1.0	No
29	Imo	Card	0.50876	32	≥ 1.0	No
30	Kogi	Penta3	0.52371	31	≥ 1.0	No
31	Plateau	Card	0.55719	30	≥ 1.0	No
32	Akwa Ibom	Penta3	0.56117	29	≥ 1.0	No
33	Rivers	Card	0.57953	28	≥ 1.0	No
34	Kwara	Penta3	0.59002	27	≥ 1.0	No
35	Benue	Child	0.62208	26	≥ 1.0	No
36	Osun	Card	0.63168	25	≥ 1.0	No
37	Cross River	Card	0.67084	24	≥ 1.0	No
38	Kogi	Card	0.69212	23	≥ 1.0	No
39	Rivers	Penta3	0.70458	22	≥ 1.0	No
40	Imo	Penta3	0.71128	21	≥ 1.0	No
41	Benue	Card	0.72281	20	≥ 1.0	No
42	Bayelsa	Child	0.72561	19	≥ 1.0	No
43	Kogi	Child	0.72722	18	≥ 1.0	No
44	Bayelsa	Penta3	0.73774	17	≥ 1.0	No
45	Ondo	Card	0.74514	16	≥ 1.0	No
46	Enugu	Card	0.75658	15	≥ 1.0	No
47	Abia	Card	0.77211	14	≥ 1.0	No
48	Bayelsa	Card	0.77762	13	≥ 1.0	No
49	Abia	Child	0.81107	12	≥ 1.0	No
50	Ekiti	Child	0.81303	11	≥ 1.0	No
51	Anambra	Child	0.83346	10	≥ 1.0	No
52	Cross River	Child	0.86140	9	≥ 1.0	No
53	Kwara	Child	0.88689	8	≥ 1.0	No
54	Plateau	Child	0.92240	7	≥ 1.0	No
55	Delta	Penta3	0.92309	6	≥ 1.0	No
56	Enugu	Child	0.92874	5	≥ 1.0	No
57	Edo	Penta3	0.94121	4	≥ 1.0	No
58	Ondo	Penta3	0.95349	3	≥ 1.0	No
59	Cross River	Penta3	0.95652	2	≥ 1.0	No
60	Rivers	Child	0.97375	1	0.97375	No

Notes:

a. Estimates are from pooled MICS and NICS survey datasets

b. Adjusted p-value = (unadjusted p-value) X (Holm-Bonferroni Multiplier)

c. Adjusted p-values >= 1.0 are clearly not statistically significant

Pooling Conclusions

- **In this dataset all adjusted p-values were > 0.01, so the data are poolable in every state**
- One adjusted p-value falls between 0.01 and 0.05, so if we had used a family-wise probability of Type I error of 5% instead of 1%, we would have concluded that the data in FCT Abuja are not poolable
- The 1% value was selected long before the data were collected, so the decision was to pool data in all states with supplementary samples

Pooling Implications

- Pooling data from core clusters and supplementary clusters made it necessary to re-calculate survey weights and re-calculate wealth quintiles
- So there are several differences between the MICS public use dataset available from the UNICEF website and the MICS-NICS public use dataset available from the Nigeria National Bureau of Statistics website:
  - The MICS-NICS dataset includes respondents from supplementary clusters
  - Even for respondents from core clusters, the values of survey weight and wealth quintile may differ between the MICS-only and the MICS-NICS datasets

Pooling Implications

- After pooling the data and re-calculating weights and wealth quintiles, the survey data analysis proceeded as though the data were all collected as part of a single effort
- Stata v14 was used to conduct the analysis
- For outcomes that were generalized to the entire population of children aged 12-23m, the analysis was weighted and confidence intervals were calculated using Taylor linearization; the confidence intervals were calculated with programs that account for the complex sample design

Strengths

- The poolability test is a good idea for situations like this where core and supplementary data will be collected prospectively – knowing that the supplementary data will be discarded if it is found to have substantively different biases should incent the collectors of that data to adopt as many practices as possible from the core data collection effort
- The test respected the clustered nature of the data by scrambling labels of clusters instead of individuals
- Without any adjustment, we would expect 60 hypothesis tests to reject the null hypothesis of poolability about three times if we used the typical 5% probability of Type I error and no adjustment for multiple tests
- We selected a more rigorous standard of 1% and we adjusted for multiple comparisons to limit the probability of discarding any data

Limitations

- Clusters that did not yield respondents were ignored in the analysis; if the likelihood of not finding any children aged 12-23m in a cluster differed between the MICS and supplementary clusters, this test is not designed to show it
- The test accounted for correlation within clusters, but did not use the survey weights; in future work, it would be straightforward to make the differences weighted instead of unweighted
- From the perspective of face validity, looking at some of the histograms, there are several observed differences that fall very far out in the tail of the distribution of random differences; they look like they would be statistically significant, but under our decision rule, they are not

Limitations

- In each state we examined differences in three outcomes where we could imagine differential bias being apparent; it is not clear whether a method that considered the joint distribution of differences would have any advantages over this approach that considered the three outcomes independently

For Future Work

- In early discussions there was a proposal of using a cut-off that was an absolute percent difference based on a committee sense of *a priori face validity*; i.e., a rule like: *If the MICS – supplementary difference is > 15%, declare the data to be not poolable.*
- But then we looked at some differences among subsets from within the MICS 2011 Nigeria dataset, we found that there can be an appreciable number of randomly jumbled divisions where the observed difference is quite large, so we switched to the randomization test
- In the future, some work to balance the face-validity of the randomization test and an easy-to-understand threshold test might be helpful

For Future Work

- Others have been developing tests like these for complex survey data
- A new paper in August 2019 looks very relevant; it would be good to correspond with the author:
  - Daniell Toth, A Permutation Test on Complex Sample Data, *Journal of Survey Statistics and Methodology*, smz018, https://doi.org/10.1093/jssam/smz018

Other References

Aickin M, Gensler H. Adjusting for multiple testing when reporting research results: the Bonferroni vs Holm methods. *Am J Pub Health* 1996;86(5):726-728.

Edgington, Eugene, and Patrick Onghena. *Randomization tests*. Chapman and Hall/CRC, 2007.

Holm S. A simple sequentially rejective multiple test procedure. *Scand J Stat* 1979;*6*(2):65–70. Available at: http://www.jstor.org/stable/4615733. Accessed June 20, 2017.

Contact Dale Rhoda with questions: Dale.Rhoda@biostatglobal.com
