# Supplementary material for: Combining cluster surveys to estimate vaccination coverage: Experiences from Nigeria’s multiple indicator cluster survey / national immunization coverage survey (MICS/NICS), 2016–17
Source: Vaccine. 2020 Sep 3;38(39):6174–83. doi: 10.1016/j.vaccine.2020.05.058 (PMC7450266; doi:10.1016/j.vaccine.2020.05.058)
Supplement: Supplementary data 2 [file mmc2.pdf]

Supplement to
Combining Cluster Surveys to Estimate Vaccination Coverage: Experiences from Nigeria's Multiple Indicator Cluster Survey / National Immunization Coverage Survey (MICS/NICS), 2016-17
Vaccine (2020) doi:10.1016/j.vaccine.2020.05.058

A Graphical Summary of Factors Contributing to Wide Penta3 Confidence Interval Half-Widths in Nigeria's 2016-17 MICS/NICS

Dale Rhoda

BIOSTAT
GLOBAL CONSULTING

Electronic Supplement

- This document is an electronic supplement to a manuscript entitled

  Combining Cluster Surveys to Estimate Vaccination Coverage: Experiences from Nigeria's Multiple Indicator Cluster Survey / National Immunization Coverage Survey (MICS/NICS), 2016-17 *Vaccine* (2020) doi:10.1016/j.vaccine.2020.05.058

- This supplement describes factors that contributed to wider-than-expected confidence interval half-widths for state level Penta3 coverage. The coverage survey report is named

  Nigeria National Immunization Coverage Survey 2016/17 (available here).

Introduction

- It is sometimes the case that a survey team calculates a sample size with the goal of yielding a two-sided 95% confidence interval (CI) that is no wider than a pre-determined limit (i.e., prevalence estimated with a CI no wider than +/- 5%).
- After the data are collected and outputs are estimated, a CI for a proportion in a complex survey can be wider than expected for three main reasons:
    1. The outcome is closer to 50% than expected.
    2. Fewer respondents were interviewed than expected.
    3. The design effect (DEFF) was higher than expected.
- If the survey planners assume conservative values for these parameters, the CI should fall in the expected range. But if one or more of the observed parameters turns out to be more extreme than the values employed in the sample size calculation, the CI can be disappointingly wide.
- In the MICS-NICS survey, all three reasons contributed to wider CIs than expected in some states, but fewer respondents than selected seems to be the reason in the most states.

Introduction

- For estimating a proportion, like vaccination coverage, the most conservative assumption is that the outcome will be 50%. When all other factors are equal, an outcome of 50% yields the widest confidence interval.

Introduction

The number of respondents interviewed can be smaller than expected for two reasons:

1. Some states like Borno and Yobe were experiencing insecurity which prevented interviewers from visiting the targeted number of clusters. See the survey report for details.
2. The expected yield of children aged 12-23m per cluster may simply be too optimistic. This appears to be the case for states in the South here.

Introduction

- The DEFF is proportional to three factors:
  1. The average number of respondents per cluster
  2. The intracluster correlation coefficient (ICC)
  3. Variability in survey weights
- This document graphs the values that were employed in what we thought were conservative sample size calculations and also graphs the observed values for these important parameters.

Note on CI Type

- For simplicity of calculation, the process of selecting which states should receive supplementary clusters and how many each should receive used calculations for Wald-type symmetric confidence intervals, but in the survey report, CI bounds were calculated using:
  - Survey-adjusted Wilson intervals when observed coverage was between 0% and 100%, and
  - Survey-adjusted Clopper-Pearson intervals when observed coverage was exactly 0% or 100%.

- These two types of interval are preferable to Wald-type intervals because they have nice properties:
  - Their endpoints always fall between 0% and 100%.
  - They are more likely to include the true population coverage figure 95% of the time.
  - Among intervals with those two properties, Wilson intervals are narrower and Clopper-Pearson intervals are comparatively wider, so the Wilson intervals are preferred when they are calculable.

Note on CI Type

- So the reader should not be surprised to find some asymmetric confidence intervals in the NICS report for outcomes where observed coverage is near 0% or 100%.

- See Dean & Pagano, 2015 for more information.
  - Dean, Natalie, and Marcello Pagano. "Evaluating confidence interval methods for binomial proportions in clustered surveys." *Journal of Survey Statistics and Methodology* 3.4 (2015): 484-503.

Figure GS-1. Expected Penta3 CI half-width using only core MICS clusters and hopefully conservative assumptions about estimated coverage, sample size and design effect.

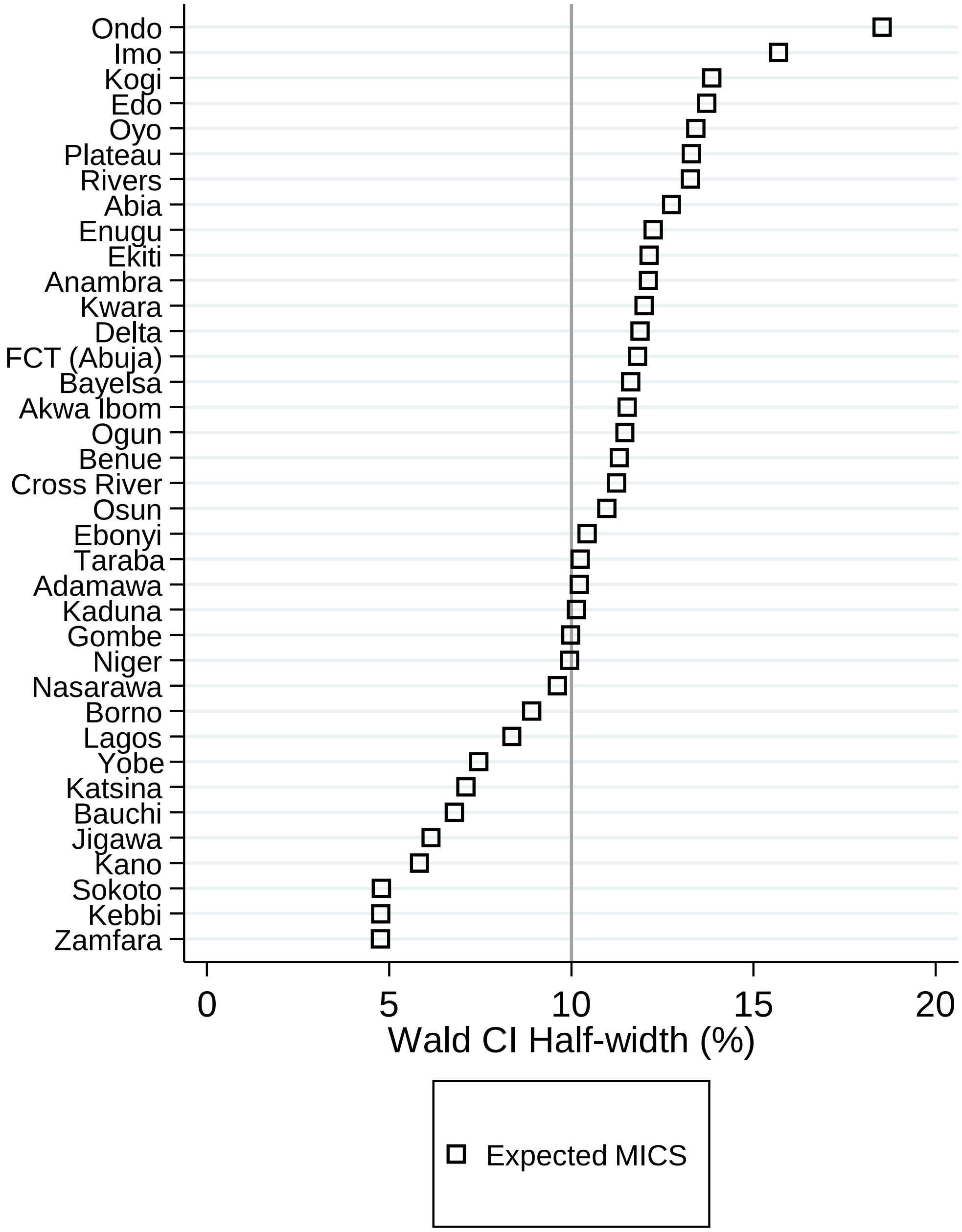

Note: Every figure in this document lists the states in this order, which is sorted from lowest to highest expected CI width if the survey had used core MICS clusters only.

Figure GS-2. By adding 10 or 20 or 30 supplementary clusters, we hope to drive the CI half-width down below 10% for all but a few states. Ondo, Imo and Kogi would have required more than 30 supplementary clusters to achieve 10%, but the number of supplementary clusters was capped at 30 so they would comprise no more than one-third of the sample in any state.

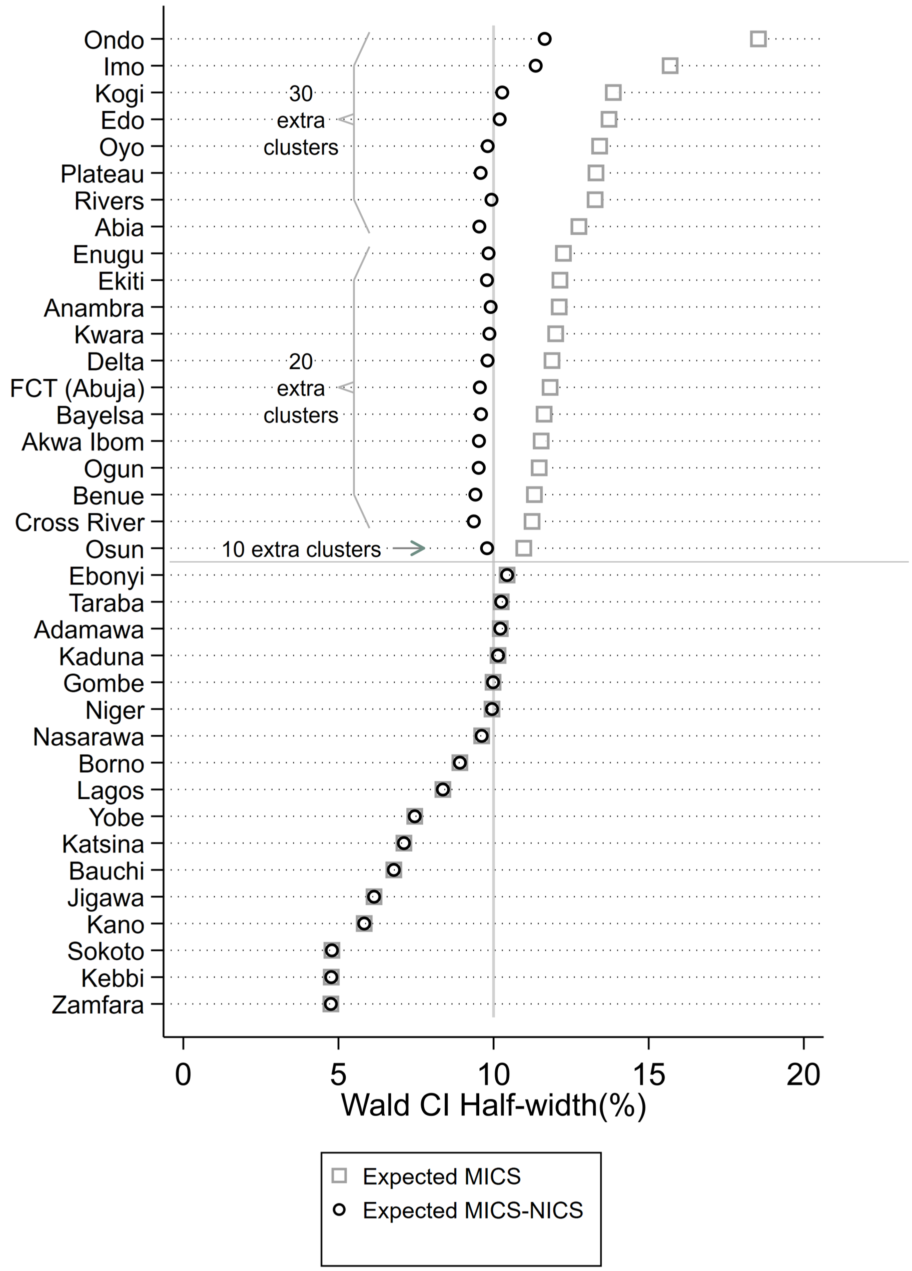

Note: All figures after this one includes the faint horizontal line between Osun and Ebonyi. The 20 states above that line were assigned some supplementary clusters.

Figure GS-3. Observed CI half-widths overlaid on the expected half-widths. The remainder of this document is a graphical exploration of possible reasons why 19 states have observed half-widths wider than 10%.

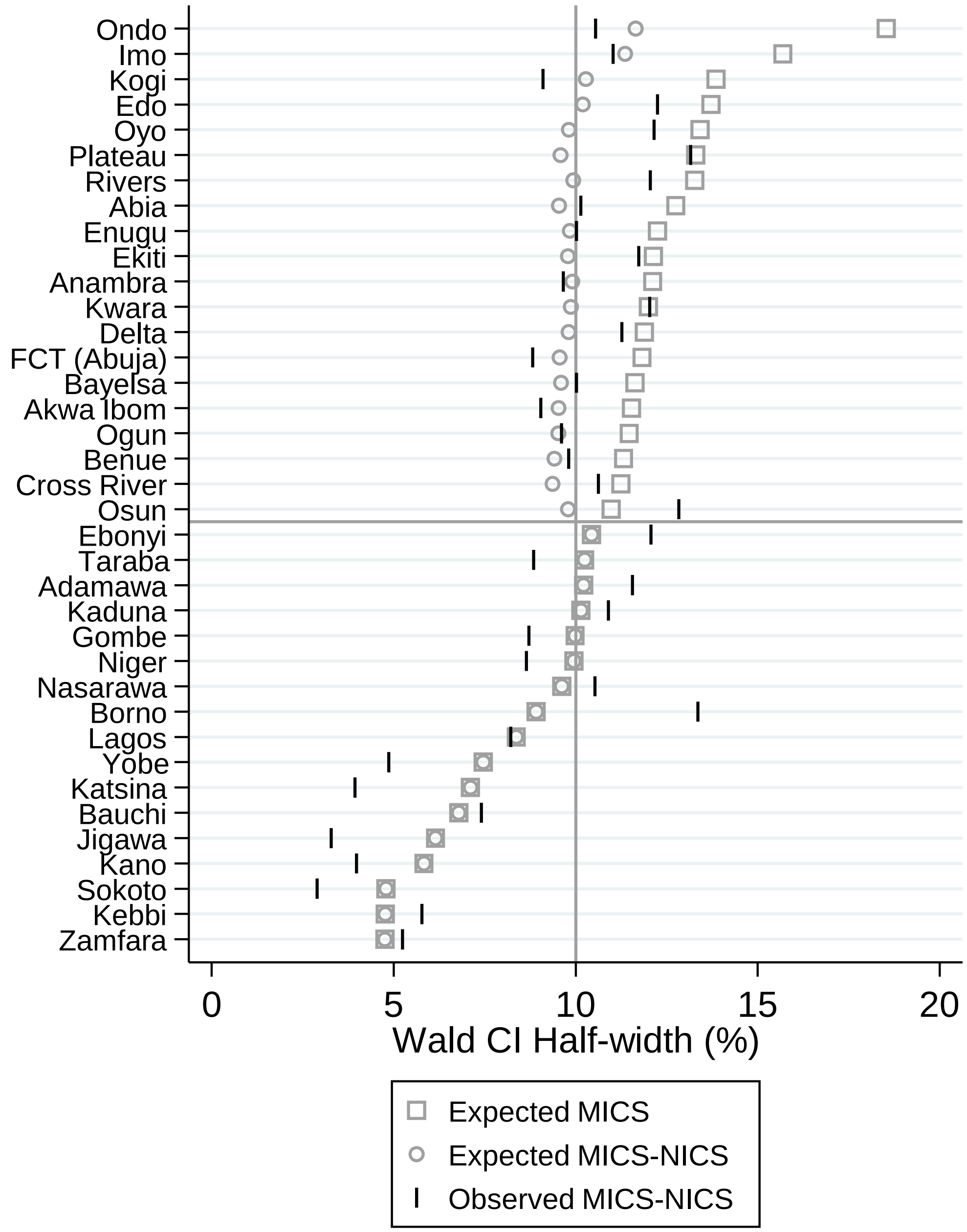

Figure GS-4. To minimize clutter and focus the reader's attention, this figure and many that follow use two panels: states with data in the left panel have an observed value that fails to meet an expectation or is more extreme than expected; states with data in the right panel met the conservative assumption. States with shaded bars had observed CI half-widths wider than 10%.

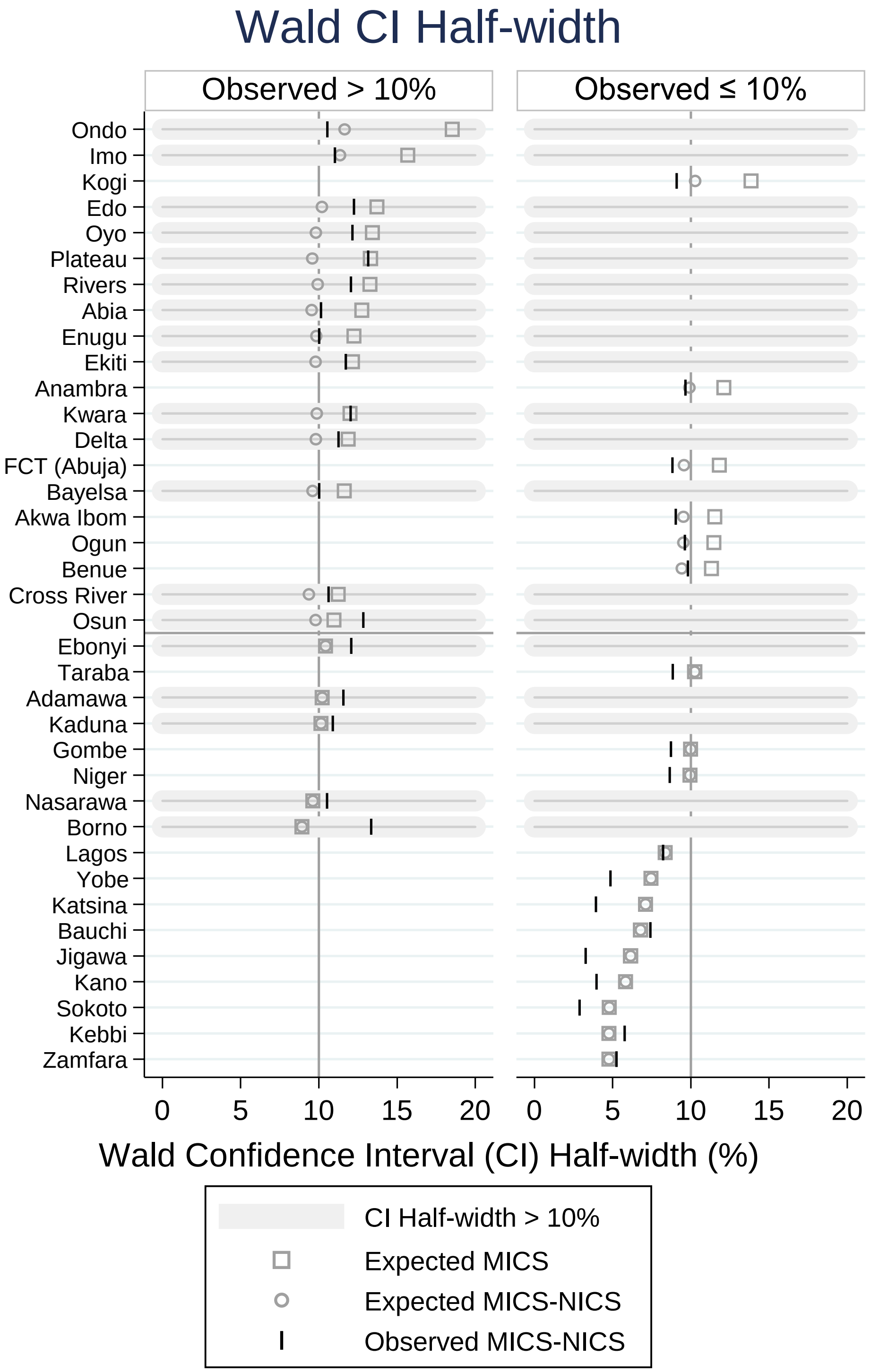

Figure GS-5. States in the left panel had observed values of Penta3 coverage that were closer to 50% than the values that had been assumed in the supplementary sample size calculations. States in the right panel had observed Penta3 coverage that was as far from 50% or farther than had been assumed in those calculations.

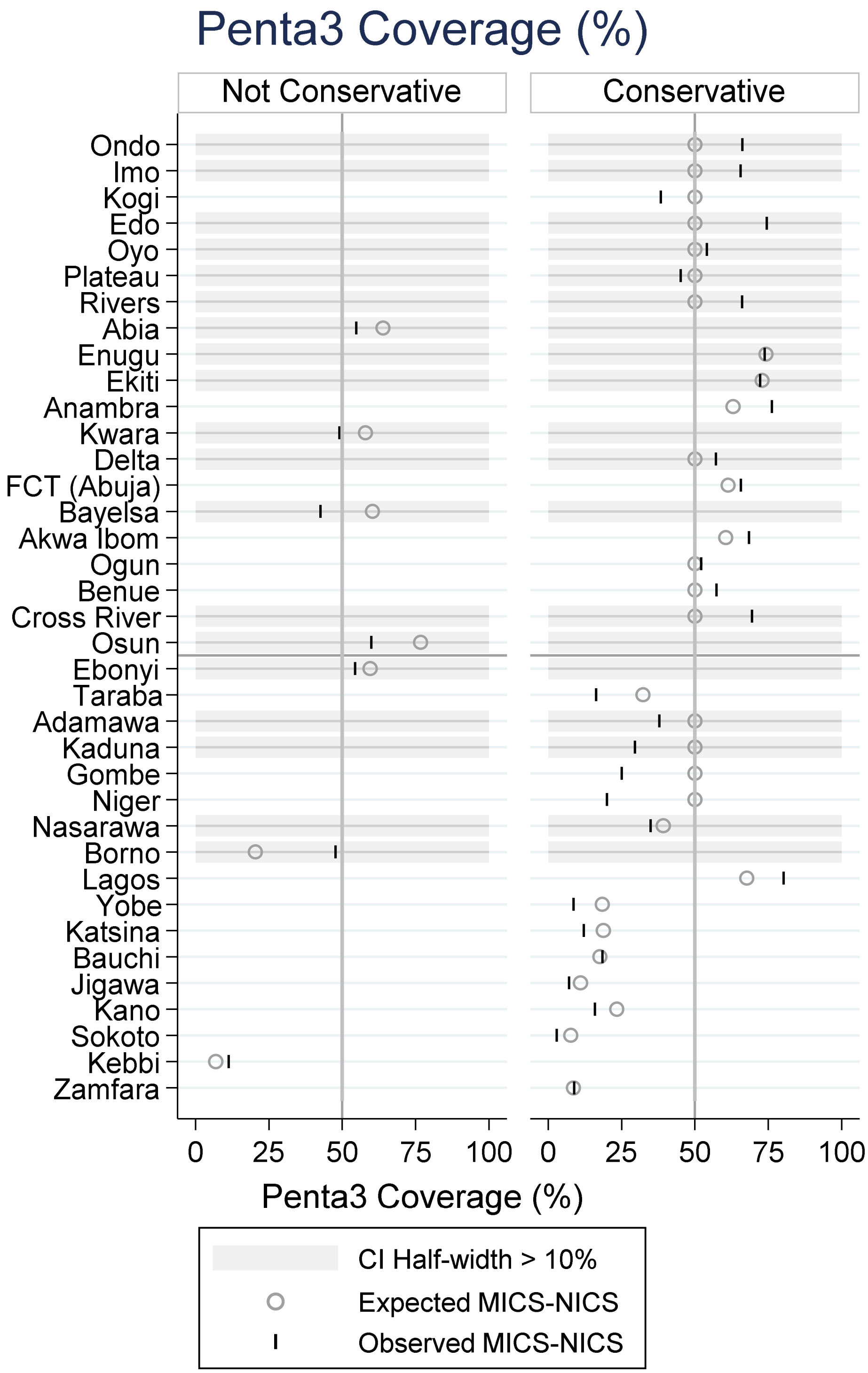

Figure GS-6. States in the left panel found children aged 12-23m in < 90% of the number of (core + supplementary) clusters that were targeted for that state. States in the right panel found children in at least 90% of the planned number of clusters. Note that failure to find children in 90% of the planned number of clusters could mean that children 12-23m were scarce, as in the South, or it could mean that the survey teams were not able to visit all the targeted clusters, as in Borno and Yobe states. See NICS report for a list of the number of clusters with interviews, by state. Finding fewer children than expected is a contributing factor in most states with wide CIs.

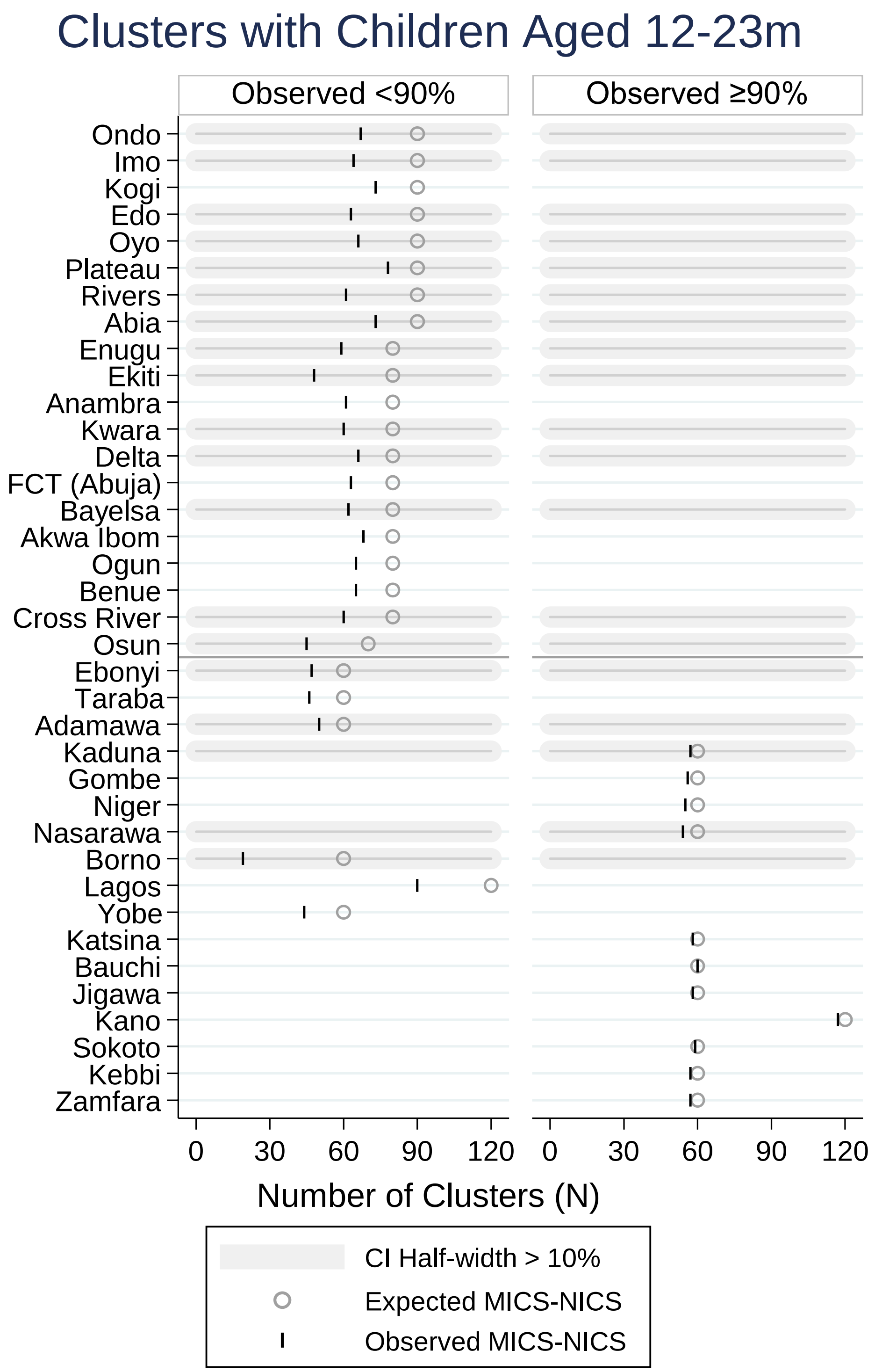

Figure GS-7. States in the left panel found <90% of the expected number of children aged 12-23m. States in the right panel found at least 90% of the number that had been projected. Finding fewer children than expected is a contributing factor in most states with wide CIs.

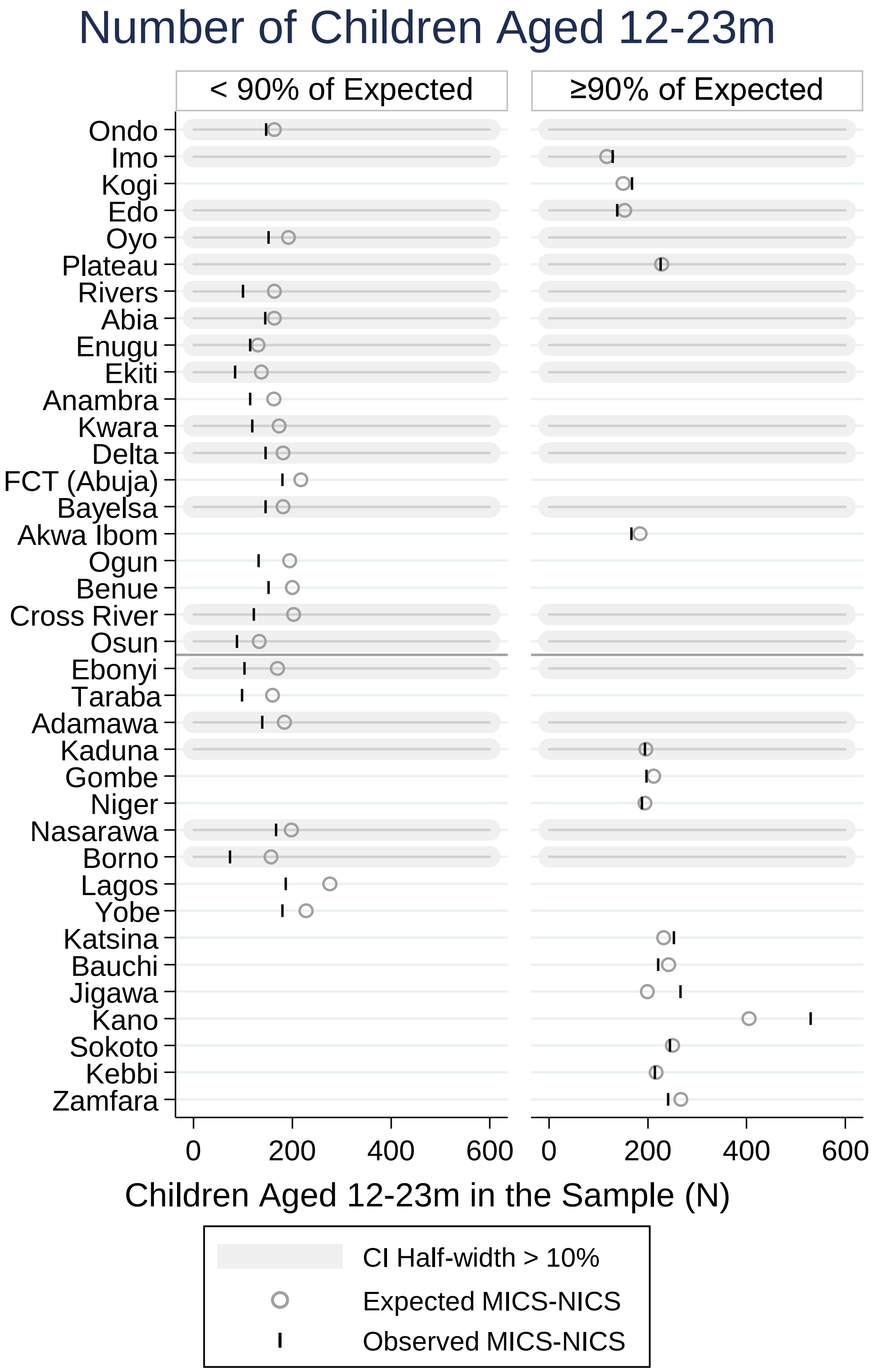

Figure GS-8. States in the left panel had an observed value of Penta3 effective sample size that was < 90% of the expected value. States in the right panel had at least 90% of the expected value. The effective sample size is the number of respondents divided by the design effect, so this figure combines observed quantities from several parameters.

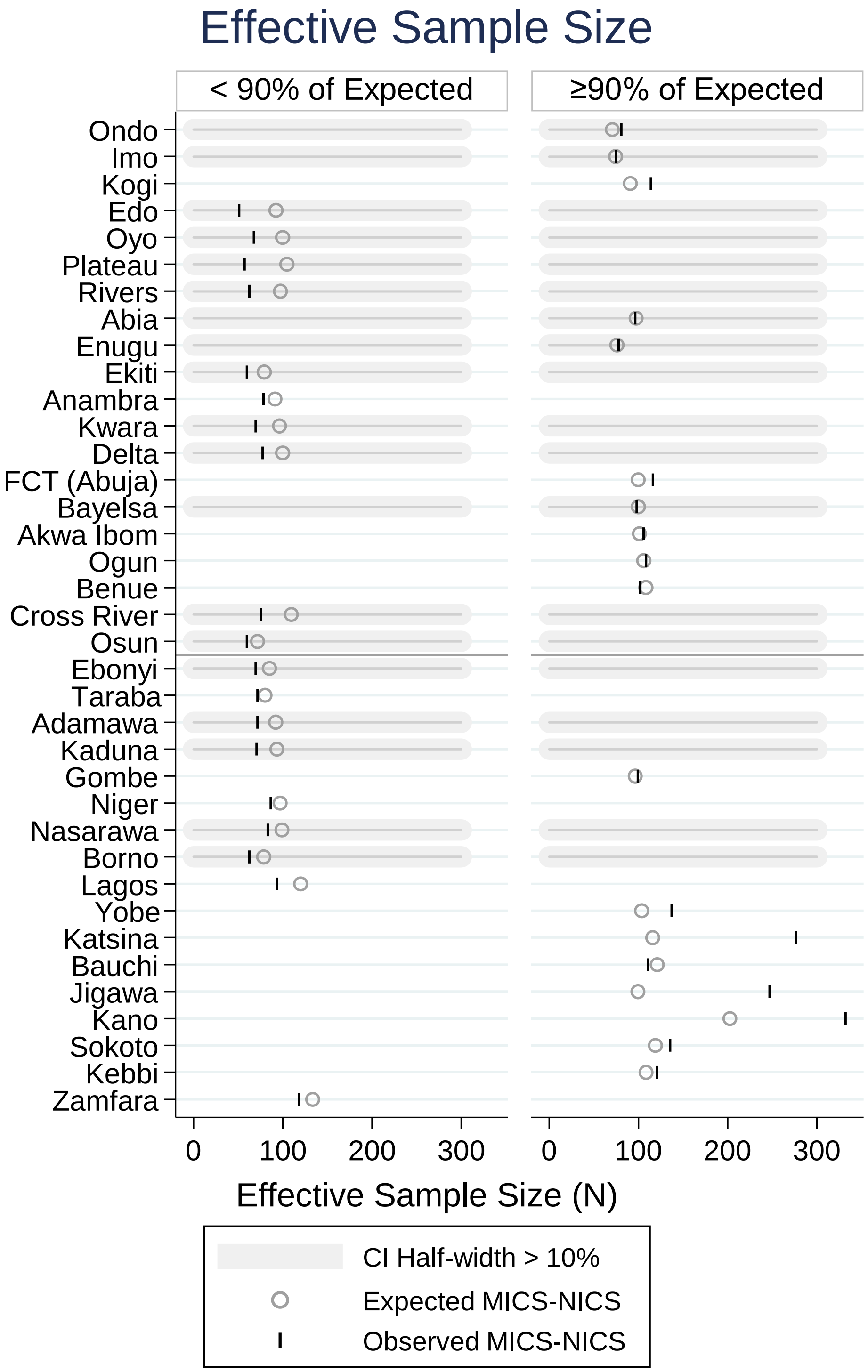

Figure GS-9. Kish (1987) separates the DEFF into two conceptual terms: one for the clustering effect and one that captures heterogeneity in weights. The second term is $(1 + CV^2_{Wt})$ where CV_{Wt} is the coefficient of variation of survey weights in the stratum. States in the left panel had an observed CV term between 1.1 and 1.4, meaning that by omitting this term from the estimated design effect, we may have under-estimated the DEFF by 10-40% in the sample size calculations. States in the right panel had CV terms between 1.0 and 1.1. [Kish, Leslie, "Weighting in Deft^2," *The Survey Statistician*, Jun-1987.]

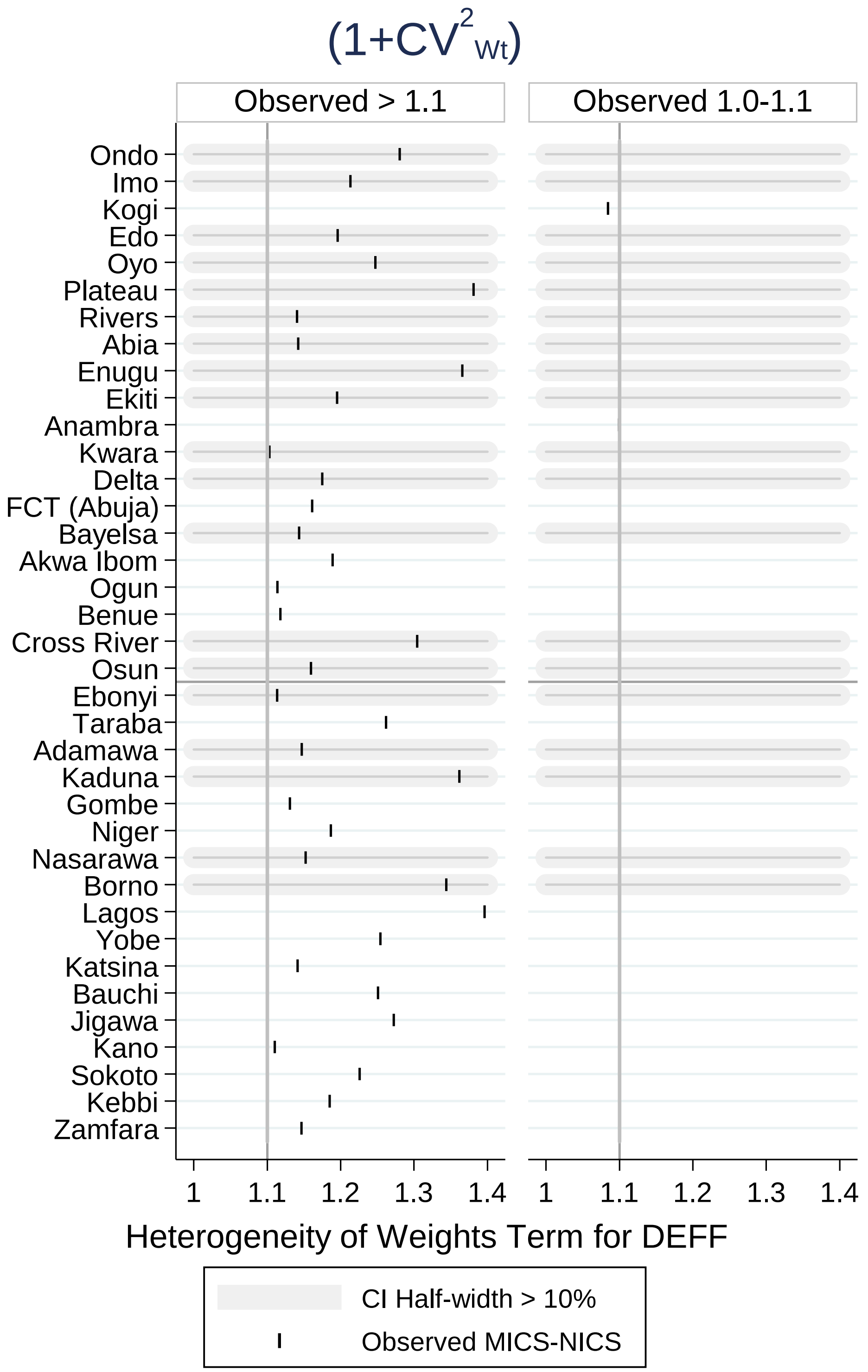

Figure GS-10. The clustering effect term of the design effect is [1 + (m-1) x ICC], where m is the average number of respondents per cluster and ICC is the intracluster correlation coefficient. This term increases linearly with ICC. The supplementary sample size calculations assumed an ICC of 1/3 for every state. States in the left panel had an observed value > 1/3 while states in the right panel had an observed value ≤ 1/3.

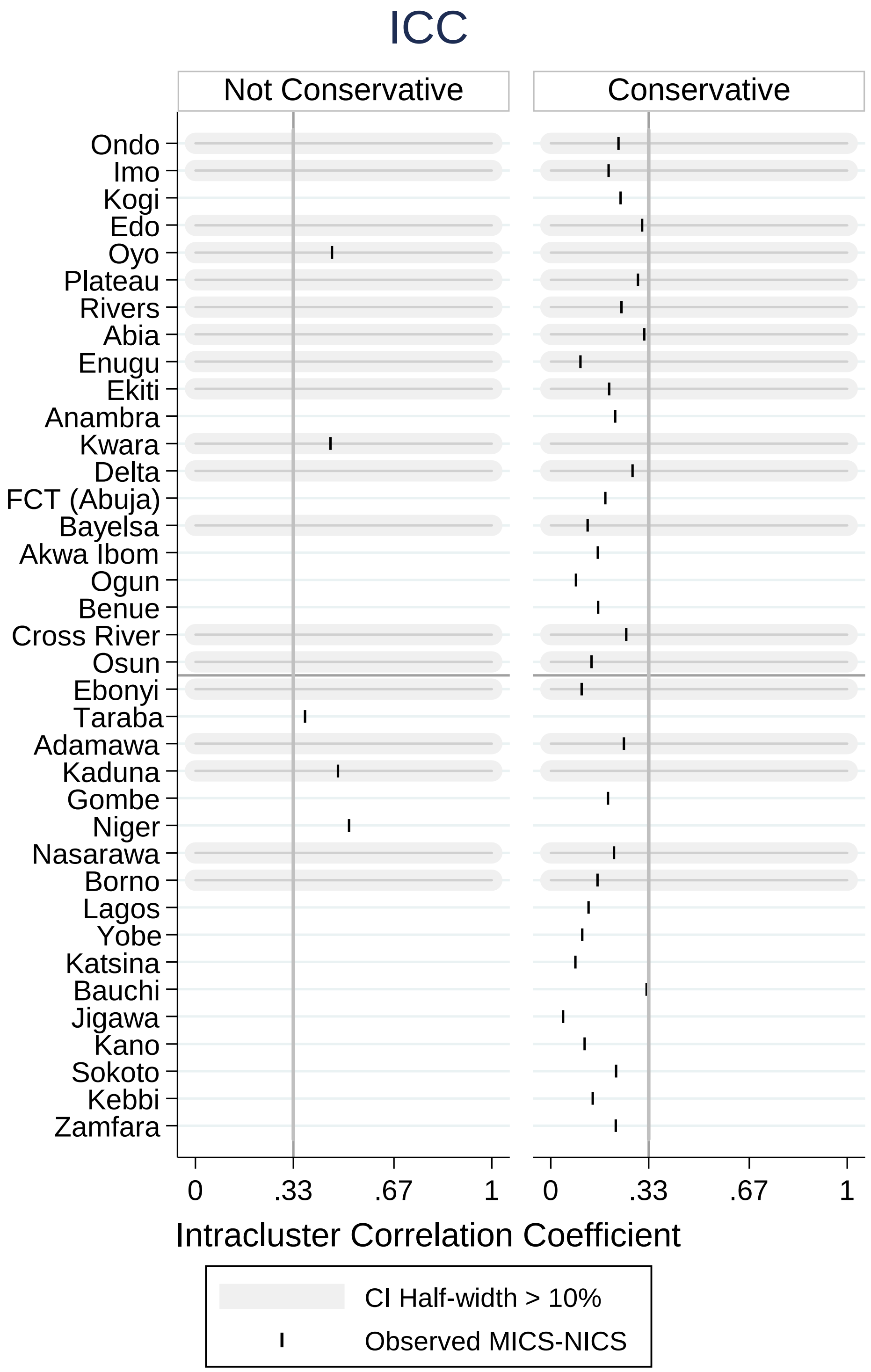

Figure GS-11. States in the left panel had an observed design effect greater than the value assumed in the supplement sample size calculations. Those in the right panel had an observed DEFF that was ≤ the value used in the calculations.

Although the sample size calculations did not account for variability in weights, the DEFF calculations turned out to be conservative for all but four states.

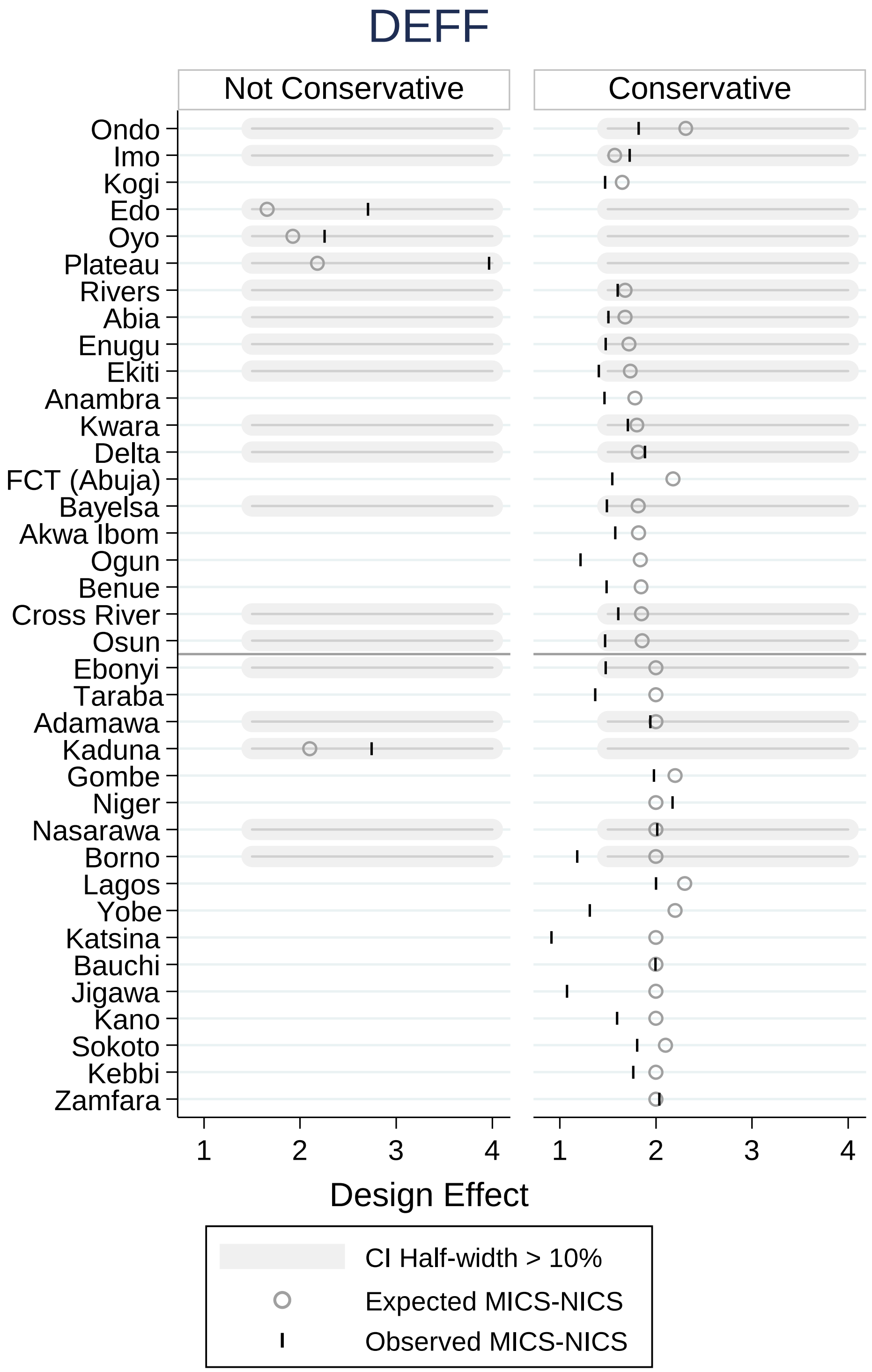

Conclusion

- Design effect contributed to the wide CIs in four states and coverage closer to 50% than expected contributed in six states, but finding fewer children aged 12-23m than expected was a contributor in 15 of the 19 states with wide CIs.
- The expected number of respondents per cluster was taken from the observed values in the 2011 Nigeria MICS. So simply put, in many states, the MICS-NICS survey delivered notably fewer children age 12-23m per cluster than did the 2011 MICS.
- Some states whose confidence interval half-widths were < 10% also had one or two parameters that were more extreme than expected but other parameters were more conservative than expected; for those states, the parameters with conservative observed values kept the CI narrow.
- Finally, note that this page is followed by a wide figure, GS-12, that uses eight side-by-side panels to summarize which parameters contributed to confidence intervals being wider than expected. It combines the left-side panels from figures GS-4 through GS-11.

Figure GS-12. Why Did Some States Have Penta3 CI Half-Width >10% ?

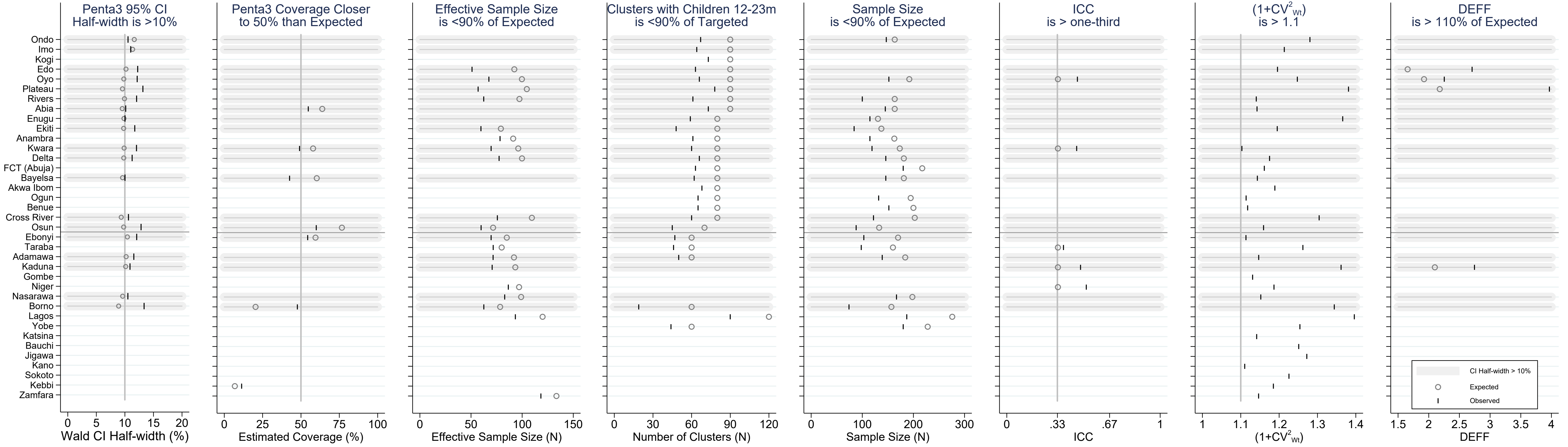

Contact Dale Rhoda with questions:

Dale.Rhoda@biostatglobal.com
