# Supplementary material for: Combining cluster surveys to estimate vaccination coverage: Experiences from Nigeria’s multiple indicator cluster survey / national immunization coverage survey (MICS/NICS), 2016–17
Source: Vaccine. 2020 Sep 3;38(39):6174–83. doi: 10.1016/j.vaccine.2020.05.058 (PMC7450266; doi:10.1016/j.vaccine.2020.05.058)
Supplement: Supplementary data 3 [file mmc3.pdf]

Supplement to

Combining Cluster Surveys to Estimate Vaccination Coverage: Experiences from Nigeria's Multiple Indicator Cluster Survey / National Immunization Coverage Survey (MICS/NICS), 2016-17

Vaccine (2020) doi:10.1016/j.vaccine.2020.05.058

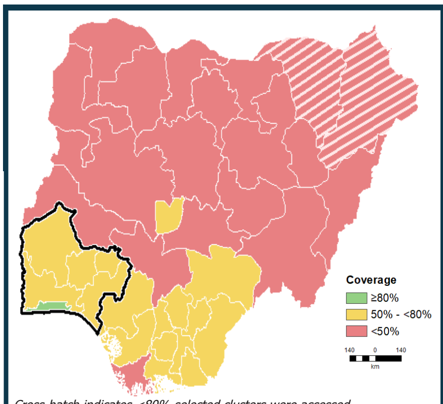

Cross-hatch indicates <80% selected clusters were accessed.
Results are representative of areas where survey could be conducted.

NATIONAL IMMUNISATION COVERAGE SURVEY

2016 2017

EXAMPLE Zone

NATIONAL PROGRESS TOWARD IMMUNISATION GOALS

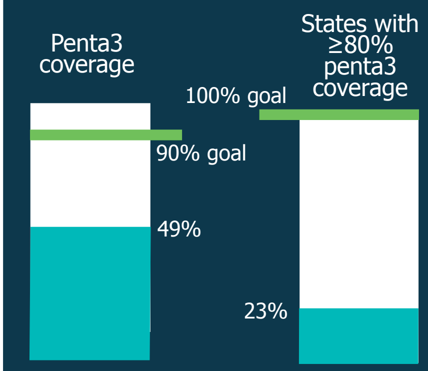

Immunisation coverage in Nigeria is far below national goals, putting a substantial number of children at risk of death and disability from vaccine preventable diseases.

Immunisation coverage varies across Nigeria but improvements are needed in *every* state.

WHY MEASURE PENTA3?

The 3rd dose of pentavalent vaccine (penta3) is an indicator of the immunisation system's ability to *reach* and *retain* children with multiple vaccinations.

MICS/NICS 2016/2017

ROUTINE IMMUNISATION IN ZONE X

2 in 3 children receive the third dose of pentavalent vaccine

30% of children start but do not complete the 3-dose series of pentavalent vaccine

52% of children had a child health card at the time of the survey

IMMUNISATION COVERAGE INDICATORS

Among children aged 12-23 months of age:	Example Zone % [95% CI]	Country % [95% CI]	Africa[3] %
First dose of pentavalent vaccine (penta1)[1]	85 [81, 90]	70 [67, 73]	83
Third dose of pentavalent vaccine (penta3)[1]	66 [61, 71]	49 [45, 55]	74
Dropout between penta1 and penta3 dose[2]	19	31	11
First dose of measles vaccine (MCV1)	74 [69, 79]	50 [44, 54]	72
Availability of child health card	52 [50, 55]	35 [31, 39]	-

[1]Crude coverage based on child health cards or parental recall. Values reported as weighted percentages (%) with two-sided 95% confidence intervals. [2]Dropout defined as percentage of children who received penta1 but did not complete 3-dose penta series. [3]WHO/UNICEF estimates, 2016.

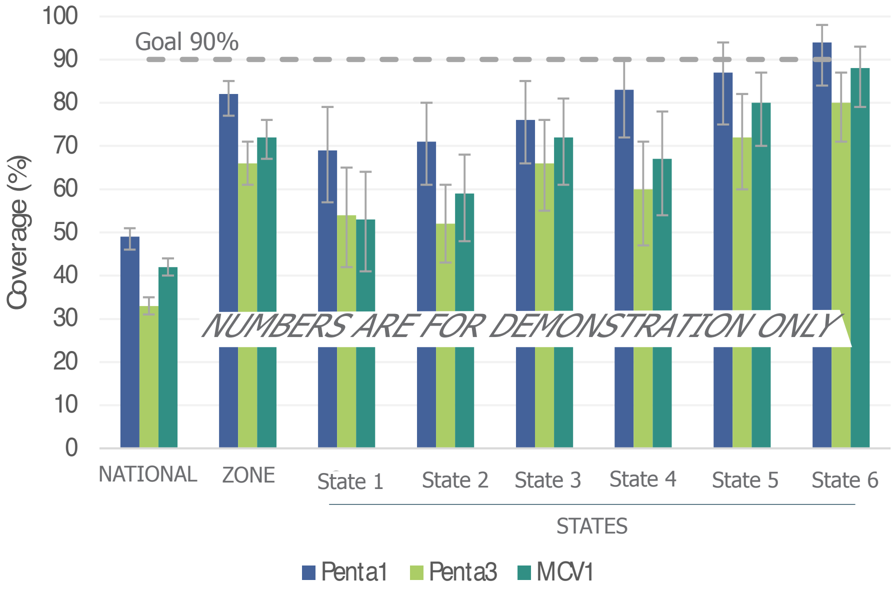

Crude coverage reported as weighted percentages (%) with two-sided 95% confidence intervals (capped lines). 90% national-coverage goal for penta1, penta3, and first dose of measles-containing vaccine (MCV1).

ZONE X: IMMUNISATION COVERAGE BRIEF

COMPLETENESS OF ROUTINE IMMUNISATION

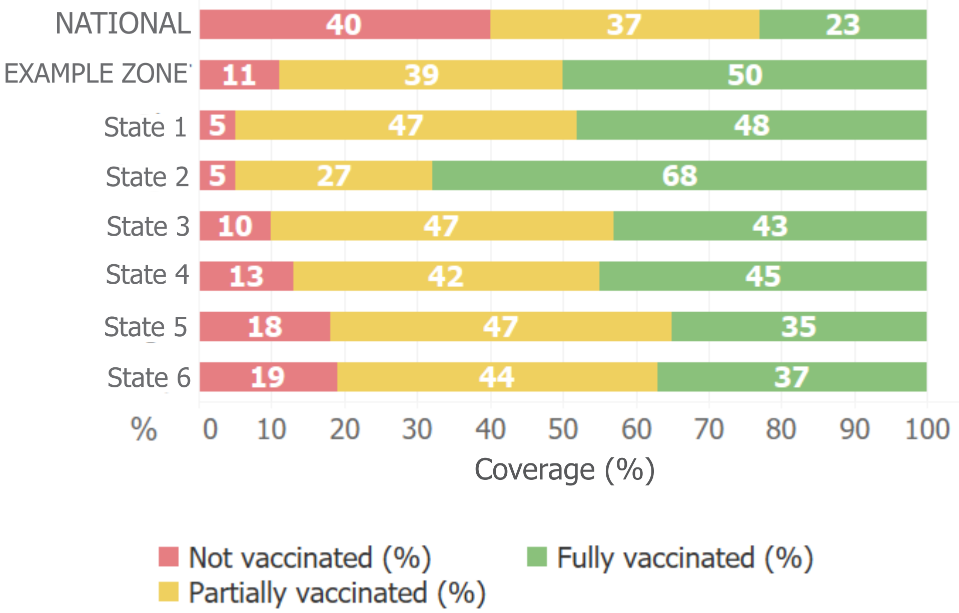

KEY FINDINGS

The benefits of vaccines are optimized when children receive all recommended vaccine doses. Only 50% of children receive all doses in zone X.

39% of children get at least one but not all vaccines in Zone X. Additional effort is needed to encourage these children to return to get all remaining vaccinations.

Lack of awareness is the main reported reason children are not fully vaccinated.

REASONS CHILDREN ARE NOT FULLY VACCINATED IN ZONE Z

28% thought child was fully immunised

44%
Lack of awareness

10% reported the vaccine was not available

17%
Service delivery issues

10% postponed immunisation

25%
Lack of time or other family issues

10% reported fear of side reactions

12%
Mistrust or fears

Note: Respondents could identify more than one reason for incomplete immunisation.

ZONE X: GAPS IN ROUTINE IMMUNISATION

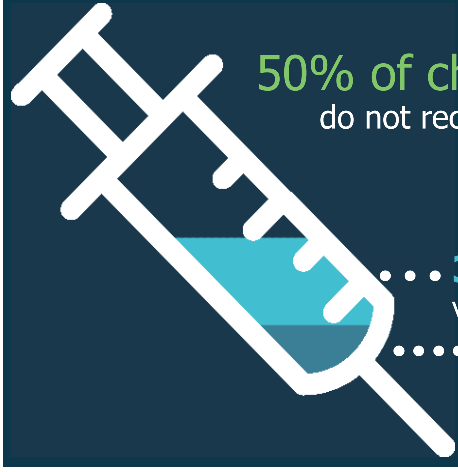

50% of children aged 12-23 months do not receive all routine immunisations in Zone X

39% receive some but not all vaccines from routine immunisation

11% do not receive any vaccines from routine immunisation

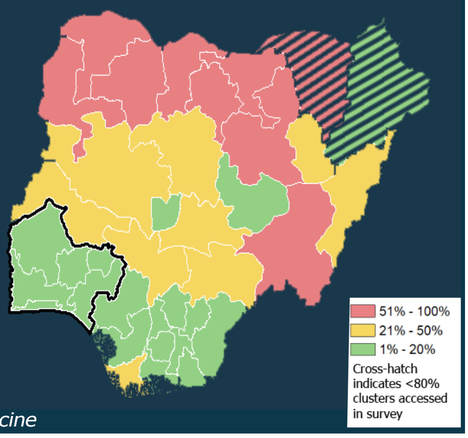

[1]Includes BCG, OPV1-3, penta1-3 & measles vaccine

DISPARITIES IN IMMUNISATION COVERAGE IN ZONE X

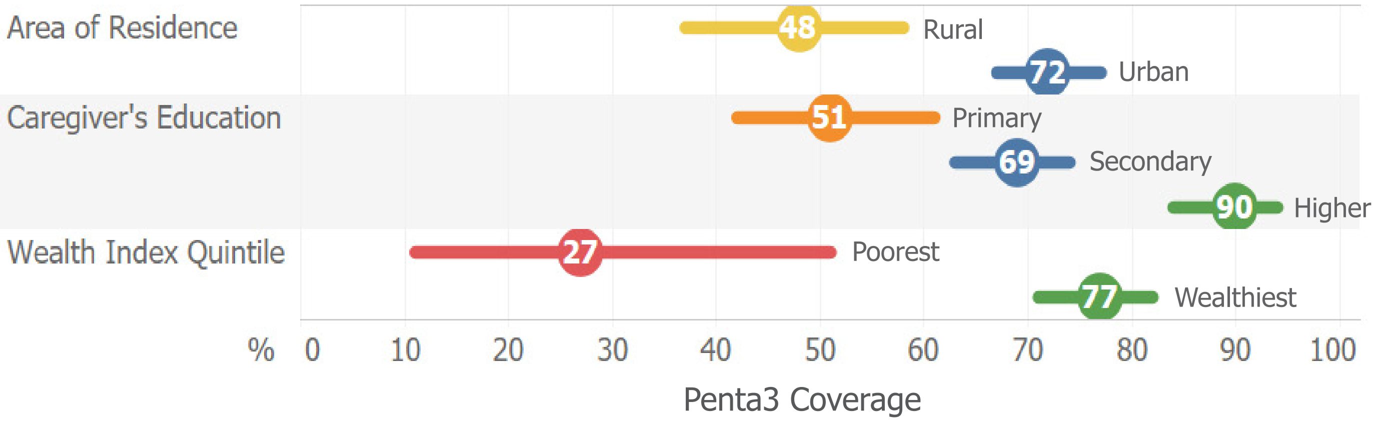

Note: Penta3 crude coverage (%) represented by circles. Bars represent upper and lower bounds of two-sided 95% confidence interval. Wealth index quintile shows the poorest 20% and richest 20% of population.

Some groups are at higher risk of incomplete vaccination:

Half of children in rural areas...

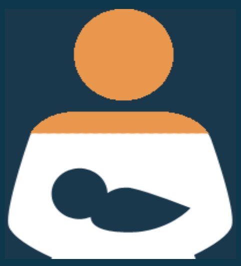

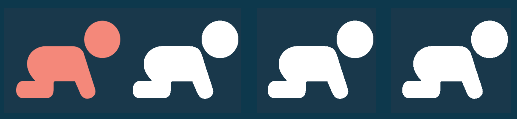

1 in 4 of the poorest children...

...receive all 3 pentavalent doses.

AVAILABILITY OF CHILD HEALTH CARDS BY STATE

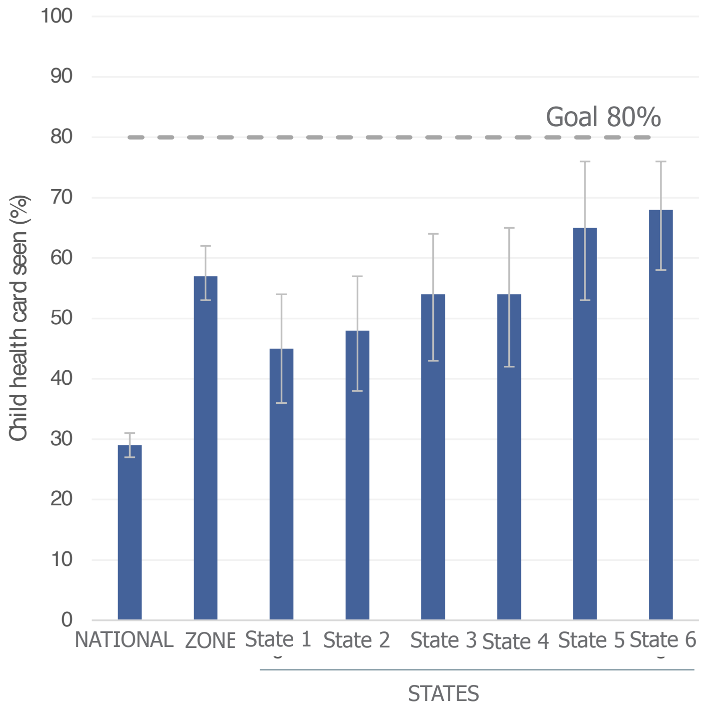

Every vaccination should be recorded on a child health card that is safely kept by the caregiver.

Child health cards help...

...caregivers know which vaccines their child has received and the date to return for the next vaccination.

...health workers make good decisions about which vaccines are overdue or not yet received.

Just over half of children in Zone X have a child health card, meaning health workers do not have the information they need to make good decisions on vaccination for many children.

CHILD HEALTH CARDS: CHECK. KNOW. PROTECT.

PUTTING NICS IN CONTEXT: OTHER PENTA3 COVERAGE ESTIMATES IN ZONE X

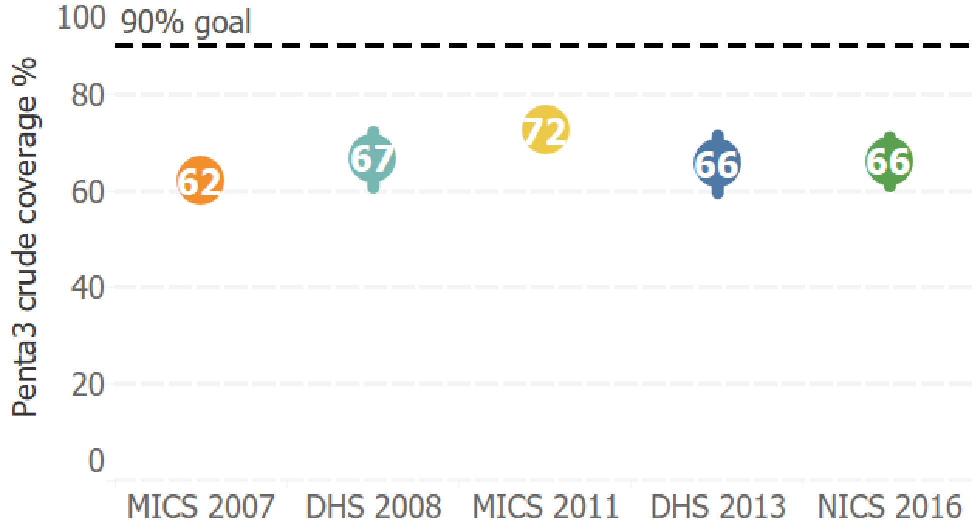

Crude penta3 coverege in North East from the Multilple Indicator Cluster Surveys (MICS) in 2007 and 2011, the Demographic and Health Surveys (DHS) in 2008 and 2013, and NICS 2016-17. 95% confidence intervals are presented for DHS and NICS surveys but may not be visible due to interval size. 95% confidence intervals are not available for MICS.

KEY FINDINGS

Immunisation coverage in Zone X has been stagnant over the past decade.

Every state in Zone X needs to improve access to routine immunisation to achieve the national goal of 90% coverage for all three doses of pentavalent vaccine.

Percentage (%) of children aged 12-23 months who received:

	BCG	Hep B at birth	Polio at birth	Polio[1] 1	Polio[1] 2	Polio[1] 3	Penta 1	Penta 2	Penta 3	Yellow fever	Measles MCV1[1]	Full[2]	Card seen	Children age 12-23 mo.
**NATIONAL**	**54**	**30**	**47**	**50**	**43**	**40**	**49**	**40**	**33**	**39**	**42**	**23**	**29**	**6,141**
Zone X	***86***	***60***	***78***	***80***	***73***	***65***	***82***	***73***	***66***	***68***	***72***	***50***	***57***	***722***
State 1	87	56	84	77	72	69	87	80	72	70	80	48	66	31
State 2	93	71	89	91	86	86	94	86	80	85	88	68	68	255
State 3	80	57	76	[illegible]	[illegible]	[illegible]	[illegible]	[illegible]	[illegible]	[illegible]	[illegible]	35	45	75
State 4	83	44	67	77	71	76	76	71	66	69	72	45	48	103
State 5	88	60	79	77	68	52	83	74	60	66	67	43	54	95
State 6	77	57	70	73	64	59	69	65	54	49	53	37	54	159

NUMBERS ARE FOR DEMONSTRATION ONLY

[1]*Does not include vaccines administered through campaigns.* [2]*BCG, OPV1-3, penta1-3 & measles from health system.*

ABOUT THE SURVEY

What is NICS?
An immunisation coverage survey conducted in Nigeria to assess coverage of vaccine antigens provided through the health system among children aged 12-23 months. Planning for NICS began in early 2015. Field work was conducted August 2016 - January 2017.

Which cohort of children was evaluated in NICS?
NICS was conducted among children who were aged 12-23 months by August 2016 - January 2017. These children should have received their vaccines between August 2014 and December 2016.

How were the field workers trained?
Field staff were trained for one month.

Who conducted the survey?
The survey was commissioned by the National Primary Healthcare Development Agency (NPHCDA) and conducted by the National Bureau of Statistics (NBS) in cooperation with state Bureau of Statistics in Kano and Lagos. Experienced field workers, primarily females, were selected from each state to facilitate communication with mothers of children.

Where was NICS conducted?
NICS was conducted in all 36 states and FCT, Abuja. Approximately 40,000 households were selected and 6,268 children were enrolled. The results provide valid national and state level immunisation coverage for children aged 12-23 months, while in Kano and Lagos, estimates are provided at the state and senatorial district level. In Yobe and Borno, <80% of selected clusters could be accessed and results are representative of areas where the survey could be conducted.

NATIONAL BUREAU OF STATISTICS

World Health Organization

unicef

BILL & MELINDA GATES foundation
